# Comparative effectiveness of various exercise modalities on arterial stiffness and endothelial function in older adults: a systematic review and network meta-analysis of randomised controlled trials

**DOI:** 10.3389/fcvm.2026.1815768

**Published:** 2026-07-13

**Authors:** Hao Wen, YaoSen Liu, Wenrui Huang, Zhihu Ma, Haibo Zhao

**Affiliations:** 1 School of Physical Education, Ningxia Normal University, Guyuan, China; 2 Department of Gynecology, The Fourth Clinical Medical College of Guangzhou University of Chinese Medicine, Shenzhen, Guangdong, China; Department of Gynecology, Shenzhen Traditional Chinese Medicine Hospital, Shenzhen, Guangdong, China; 3 School of Physical Education and Health, Ningxia Medical University, Yinchuan, China

**Keywords:** arterial stiffness, endothelial function, exercise modalities, network meta-analysis, vascular ageing

## Abstract

**Objective:**

We aimed to evaluate the comparative modulatory efficacy of various exercise modalities on endothelial function and arterial stiffness in older adults via a systematic review and network meta-analysis.

**Methods:**

We systematically searched eight electronic databases to identify randomised controlled trials (RCTs) evaluating seven distinct exercise modalities (aerobic, walking, resistance, combined, mind-body, stretching, and whole-body vibration training) in older adults aged 60 years or older. Eligibility criteria stipulated supervised, standardised training programmes with a minimum duration of 4 weeks and continuous monitoring of exercise intensity. To comprehensively evaluate the comparative efficacy of these modalities on arterial stiffness and endothelial function, a frequentist network meta-analysis was performed to mathematically integrate direct and indirect trial evidence. The certainty and quality of evidence were rigorously assessed via the Cochrane RoB 2.0 and CINeMA frameworks.

**Results:**

In primary analyses, whole-body vibration and resistance training ranked highest for enhancing endothelial function (FMD), whereas aerobic training and walking were associated with greater improvements in attenuating arterial stiffness (baPWV); However, extensive confidence interval overlap precluded definitive claims of clinical superiority. Regarding secondary outcomes, stretching exercise showed a higher probability of reducing systolic blood pressure (SBP) based on mean ranking estimates, while combined training ranked among the leading modalities for lowering diastolic blood pressure (DBP), though these findings warrant cautious interpretation as head-to-head differences generally lacked statistical significance. The observed reductions in blood pressure, particularly systolic decreases exceeding 10 mmHg, carry substantial clinical relevance, given that a reduction of this magnitude is typically associated with an approximate 20% down-risk in major adverse cardiovascular events within older populations. According to the CINeMA framework, the certainty of evidence was graded as moderate-to-high for FMD outcomes, but low-to-moderate for arterial stiffness comparisons.

**Conclusion:**

Accumulating evidence suggests that exercise-induced vascular adaptations in older adults are likely modality-specific. Whole-body vibration and resistance training appear to confer substantial optimisation upon endothelial function, whereas aerobic training and walking demonstrate potential advantages in attenuating arterial stiffness. Rather than supporting an immediate paradigm shift, these findings suggest that integrating exercise monitoring tailored to specific phenotypic characteristics may refine clinical prescriptions, though such implementation should be interpreted with caution given inherent study heterogeneity, reliance on indirect network comparisons, and potential risks of bias.

**Systematic Review Registration:**

https://www.crd.york.ac.uk/PROSPERO/view/CRD420251236872, identifier CRD420251236872.

## 1 Introduction

Vascular ageing represents the age-associated structural and functional deterioration of the vasculature (1). This process is driven primarily by oxidative stress, chronic inflammation, and extracellular matrix remodelling (2, 3). These molecular mechanisms initiate pathological shifts that increase arterial stiffness, which sequentially elevates systolic blood pressure and widens pulse pressure. Ultimately, this haemodynamic cascade predisposes patients to left ventricular hypertrophy and diastolic heart failure (4). Longitudinal evidence reveals that the prevalence of high-risk aortic stiffness exceeds 60% among individuals aged 70 years and older (4). Although ubiquitous, the clinical impact of vascular ageing is frequently underestimated, with this progressive compromise markedly heightening all-cause mortality and consequently contributing to a substantial long-term socioeconomic burden on par with other major chronic pathologies (5). Current international guidelines recommend renin–angiotensin–aldosterone system (RAAS) inhibitors as first-line therapy (6). Nevertheless, even among patients attaining target blood pressure, up to 40% continue to exhibit elevated central arterial stiffness, representing a substantial residual vascular risk (7). Although effective, pharmacological management in older adults is frequently limited by polypharmacy, which increases vulnerability to adverse drug events, falls, and suboptimal adherence (8). This clinical limitation, combined with the high rate of residual structural risk, creates a critical therapeutic gap for safe, well-tolerated, non-pharmacological strategies capable of conferring comprehensive vascular protection. To fill this gap, exercise training emerges as a necessary direct countermeasure, uniquely delivering biomechanical shear stress and metabolic transduction that reverse the structural matrix remodelling and endothelial decay intrinsic to vascular ageing, whereas conventional drugs predominantly target neurohormonal pathways merely to mitigate pressure loading.

Vascular ageing underpins the escalating burden of cardiovascular disease in older adults. To reverse this degenerative process, global authorities including the World Health Organization (WHO) and the American College of Sports Medicine (ACSM) advocate exercise as a cornerstone non-pharmacological strategy (9, 10). Recent randomised controlled trials (RCTs) indicate that distinct exercise modalities operate through divergent mechanobiological and neurophysiological pathways (11). Specifically, active modalities like aerobic and resistance training modulate vascular phenotypes through shear stress-mediated endothelial remodeling or metabolic pathways (12, 13), while alternative strategies including mind-body exercise and whole-body vibration engage passive mechanical or autonomic pathways (14, 15). Despite these disparate mechanisms, the relative efficacy of these interventions on vascular structure and function remains uncompared (16). This evidence gap precludes the formulation of precision-based exercise prescriptions tailored to older adults (17).

Previous systematic reviews rely almost exclusively on traditional pairwise meta-analyses, which merely confirm the efficacy of single modalities (such as aerobic or resistance training) against sedentary controls (9). However, due to a profound dearth of direct, head-to-head trials comparing multiple active interventions simultaneously, these conventional pooled estimates cannot determine the relative therapeutic superiority among competing exercise types. Consequently, available evidence remains clinically insufficient to guide precision-based exercise prescriptions (18). Despite an increasing number of discrete RCTs (19), a definitive consensus on which specific modality optimally attenuates arterial stiffness and enhances endothelial function in older adults remains elusive (20). To resolve this dilemma, we conducted a network meta-analysis to mathematically integrate direct and indirect evidence, bypassing the lack of head-to-head trials to quantitatively rank the relative effectiveness of seven distinct modalities. This hierarchical ranking provides unique added value by transitioning uniform physical activity advice into precise, phenotype-specific cardiovascular prescriptions.

## 2 Methods

The protocol for this systematic review and network meta-analysis was prospectively registered with PROSPERO (registration number CRD420251236872). This study was conducted and reported in adherence to the Preferred Reporting Items for Systematic Reviews and Meta-Analyses (PRISMA) 2020 statement and its specific extension for network meta-analyses (PRISMA-NMA) (21, 22).

### 2.1 Search strategy

We systematically searched PubMed, Web of Science, the Cochrane Central Register of Controlled Trials (CENTRAL), Embase, and four Chinese databases (VIP, Wanfang Data, SinoMed, and CNKI) from their respective inception dates through to Oct 21, 2025, to identify RCTs assessing the comparative efficacy of various exercise modalities on vascular health in older adults. Two reviewers independently performed the literature search and study selection in duplicate, with any discrepancies resolved via consultation or adjudication by a third reviewer. This electronic search was supplemented by forward and backward citation tracking of eligible articles and pertinent systematic reviews, alongside manual screening of ClinicalTrials.gov and major international sports medicine and cardiovascular conference proceedings to exhaustively capture unindexed academic outputs and the latest data from nascent trial pipelines. Full details of the search strategies for each database are provided in Supplementary Appendix 1.

### 2.2 Eligibility criteria

Study selection was governed by the PICOS framework; RCTs published in English or Chinese were eligible for inclusion provided they fulfilled predefined eligibility criteria.

#### Participants (P)

participants aged 60 years or older, or cohorts explicitly defined as elderly or postmenopausal women. The targeted study population encompassed both healthy older adults and those presenting with stable chronic comorbidities. To rigorously control for baseline clinical heterogeneity and comprehensively evaluate the impact of divergent vascular health status, the eligible study population was prospectively stratified into two distinct clinical phenotypes:

(1) The Physiological Vascular Ageing Cohort: Enrolled individuals were older adults or postmenopausal women free of active metabolic disorders, without concurrent exposure to vasoactive medications known to modulate endothelial function, and devoid of established cardiovascular or peripheral arterial disease.
(2) The Pathological Endothelial Dysfunction Cohort: Enrolled individuals were those with a confirmed baseline diagnosis of cardiometabolic comorbidities, including stage 1 or 2 hypertension, type 2 diabetes mellitus, hyperlipidaemia, or peripheral arterial disease.

To guarantee clinical stability within the comorbidity cohort, participants were required to have received an optimized and stable pharmacological regimen for at least 3 months prior to enrollment, during which no new acute cardiovascular events occurred and no modifications were made to their therapeutic regimens. Furthermore, all participants across both cohorts were required to be sedentary or physically inactive at baseline, operationally defined as the absence of regular physical exercise during the preceding 3–6 months. Based on this categorical stratification, prespecified subgroup analyses (Supplementary Appendix 11) were performed across five key indicators, including flow-mediated dilation (FMD), carotid-femoral pulse wave velocity (cfPWV), brachial-ankle pulse wave velocity (baPWV), systolic blood pressure (SBP), and diastolic blood pressure (DBP), in order to statistically evaluate the potential effect modification by population heterogeneity on the therapeutic efficacy of exercise.

#### Interventions (I)

Interventions were categorized into seven distinct modalities based on their primary training stimulus and mechanical delivery, adapted from the American College of Sports Medicine (ACSM) guidelines for physical conditioning (23) and multi-component exercise frameworks for older cohorts (24), as detailed below:

1. Mind–Body Exercise (MBE): This modality requires the concurrent incorporation of three core components: (1) active cognitive regulation (e.g., mindfulness training or focused attentional guidance); (2) regulated deep breathing; and (2) low-to-moderate intensity somatic postural control. Interventions solely reliant on mechanical muscular loading or high-intensity cardiopulmonary energy expenditure fall outside this scope (25).
2. Stretching Exercise (SE): This training comprises active or passive joint mobilization with the exclusive objective of systematically elongating skeletal muscle-tendon units. It involves no application of load to overcome external resistance and imposes no sustained cardiopulmonary metabolic demand (26).
3. Resistance Training (RT): This modality is characterized by the execution of isotonic, isometric, or isokinetic muscular contractions against external mechanical loads (27).
4. Whole-Body Vibration (WBV): Participants are positioned on a mechanical vibration platform to directly receive mechanical oscillatory waves characterized by specific frequencies and amplitudes, executed via either static postural maintenance or dynamic loading (28, 29).
5. Aerobic training (AT): This involves sustained, rhythmic exercise utilizing large muscle groups designed to induce progressive cardiopulmonary stress (with flat-surface walking lacking a flight phase being excluded), with the exercise intensity explicitly exceeding specific cardiopulmonary metabolic thresholds (23).
6. Walking Training (WT): Kinematically defined as active, self-propelled, forward bipedal locomotion executed within a gravitational field (30).
7. Combined Training (CT): This represents the structured integration of two or more distinct exercise modalities within either the same overarching intervention program or a single exercise session, with the combination of AT and RT being the most frequent configuration (31).

#### Comparators (C)

Eligible comparators comprised sedentary controls, attention controls (e.g., health education lectures), or any of the aforementioned exercise modalities. Studies were excluded if they involved passive interventions, unsupervised lifestyle advice, or inseparable co-interventions, such as dietary or pharmacological modifications, that precluded the isolation of exercise-specific effects from potential confounding variables.

#### Outcomes (O)

Studies were eligible for inclusion if they reported at least one primary outcome. Primary outcomes comprised gold-standard markers of macrovascular endothelial function and regional arterial stiffness, specifically (1) flow-mediated dilatation (FMD), representing endothelium-dependent, nitric oxide (NO)-mediated conduit artery vasodilation (32), (2) carotid–femoral pulse wave velocity (cfPWV), the reference standard for central aortic stiffness (33), and (3) brachial–ankle pulse wave velocity (baPWV), a validated composite measure of central and peripheral arterial stiffness (34). Secondary outcomes evaluated systemic wave reflection, peripheral hemodynamics, localized arterial mechanics, and somatic status. These parameters included (1) systemic wave reflection measured via the augmentation index (AIx) and heart rate-standardized augmentation index at 75 beats per minute (AIx@75) (35), (2) systemic hemodynamics encompassing systolic and diastolic blood pressure (SBP and DBP), (3) localized vascular markers comprising the carotid *β*-stiffness index (*β*-index) (36) and ankle–brachial index (ABI) (34), as well as (4) body mass index (BMI) as a somatic covariate.

#### Study design (S)

Eligibility was restricted to peer-reviewed RCTs; crossover and non-inferiority trials were excluded. We further excluded animal studies, reviews, conference abstracts, dissertations, and duplicate publications. To ensure a focused assessment of chronic training adaptations and maintain rigorous clinical safety standards, trials investigating acute exercise interventions (single-bout effects) or those involving participants with unstable cardiovascular disease were considered ineligible for inclusion.

### 2.3 Screening process

**Figure 1:**
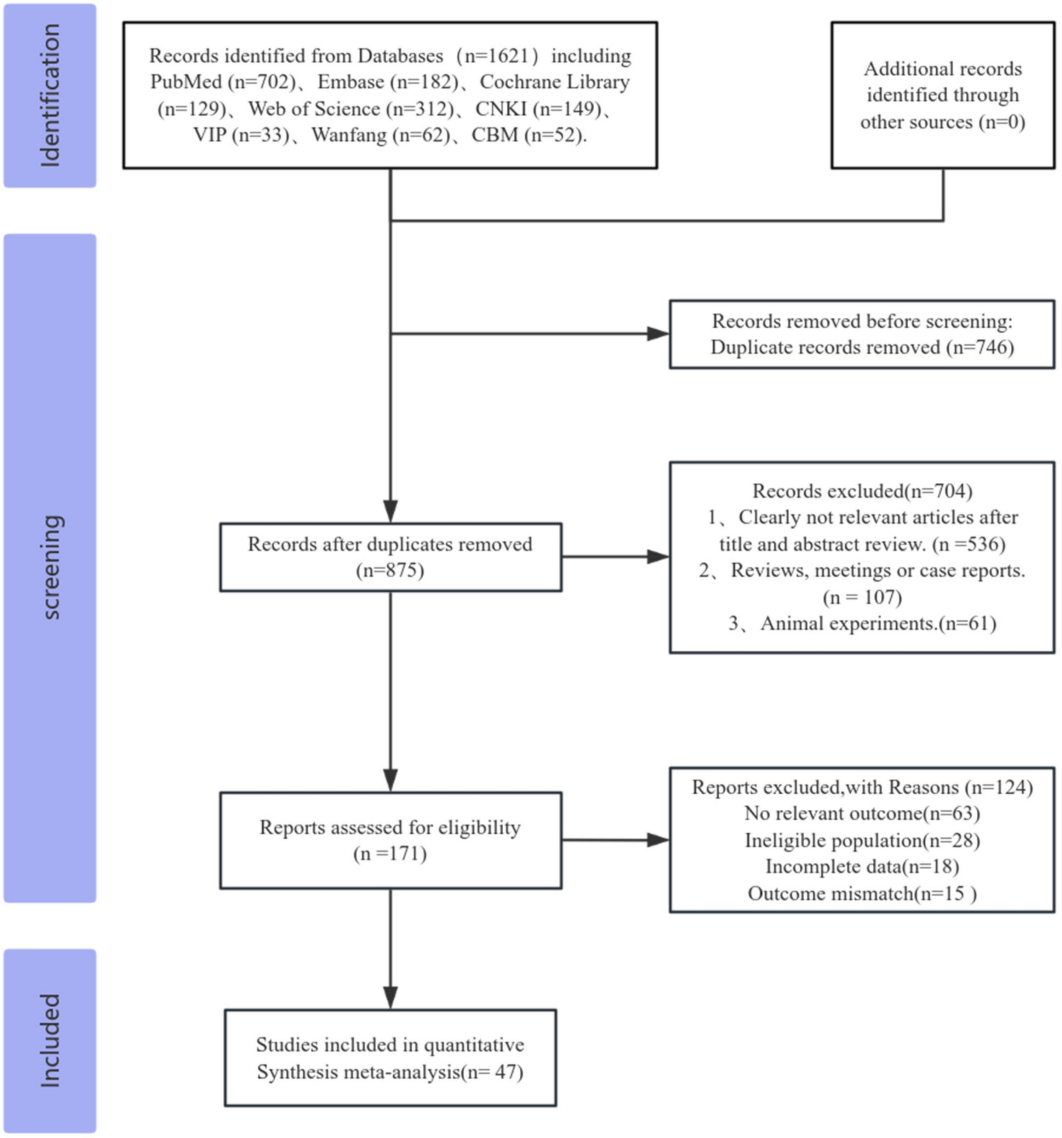
Flowchart of literature search and selection process.

Retrieved records were imported into EndNote for deduplication, with literature screening executed in strict accordance with the PRISMA guidelines. Two reviewers independently evaluated the studies across three successive phases: (1) title screening, (2) abstract screening, and (3) full-text evaluation against the predefined eligibility criteria. Any discrepancies at any stage were resolved via consensus-reaching discussions or adjudicated by a third senior investigator. Specific reasons for study exclusion at the full-text level are explicitly documented in the accompanying PRISMA flow diagram (Figure 1).

### 2.4 Data extraction and management

To facilitate a robust comparison of the efficacy of distinct exercise modalities and to validate the transitivity assumption fundamental to network meta-analysis, data were extracted across the following predefined domains: (1) Study characteristics: Including the first author, publication year, country of origin, trial design (parallel-group or crossover), total sample size, and follow-up retention rates. (2) Participant characteristics: Clinically relevant covariates encompassed age, sex distribution, BMI, baseline blood pressure, smoking status, and menopausal status. (3) Intervention details: Exercise protocols were parameterized according to the FITT (frequency, intensity, time, and type) framework. Variables extracted for quantitative synthesis were restricted to training frequency (sessions per week), session duration (minutes per session), overall intervention length (weeks), and classified exercise modalities, alongside descriptive markers of prescription intensity.

(4)Control groups: Comparator arms were meticulously classified into sedentary controls, usual care, or health education, with the inactive sedentary cohort established as the common baseline reference node. Network meta-analysis was implemented within a multivariate random-effects framework that simultaneously synthesized direct head-to-head trials and indirect evidence, thereby generating pooled mean differences or standardised mean differences for all pairwise therapeutic comparisons across the unified network geometry.(5)Outcomes: The primary indices for arterial stiffness were cfPWV and baPWV. To ensure analytical consistency, raw velocity data were harmonised into the standardised unit of meters per second (m/s). The ABI and AIx were extracted as supplementary markers. Vascular endothelial function was primarily evaluated via FMD, while SBP and DBP were recorded as hemodynamic parameters.

### 2.5 Quality assessment of evidence

Risk of bias in the included trials was appraised using the Cochrane Risk of Bias tool for randomised trials (RoB 2.0), which evaluates five distinct domains: the randomisation process, deviations from intended interventions, missing outcome data, measurement of the outcome, and selection of the reported result (37). The overall risk of bias was classified as low (score 1) only if all domains were deemed to be at low risk. Conversely, an overall high risk of bias (score 3) was assigned if at least one domain was flagged as high risk; All other instances were categorised as having “some concerns” (score 2). Data extraction, encompassing study characteristics, intervention details structured via the FITT framework, and vascular outcomes, was performed independently by two reviewers, with any discrepancies adjudicated by a third senior researcher. Finally, the certainty of evidence was evaluated according to the CINeMA framework across six critical domains: within-study bias, reporting bias, indirectness, imprecision, heterogeneity, and incoherence (38, 39).

### 2.6 Data synthesis and analysis

A network meta-analysis of randomised controlled trials was conducted using Stata (version 17.0; StataCorp, College Station, TX, USA). Within a frequentist framework, the multivariate network meta-analysis was implemented via the mvmeta command (40, 41), thereby avoiding subjective prior distributions and ensuring high computational stability for continuous metrics. Relative treatment effects for continuous outcomes, specifically flow-mediated dilation (FMD), carotid–femoral pulse wave velocity (cfPWV), brachial–ankle pulse wave velocity (baPWV), systolic blood pressure (SBP), and diastolic blood pressure (DBP), were estimated as mean differences (MDs) or standardised mean differences (SMDs) with corresponding 95% confidence intervals (CIs). Potential small-study effects and publication bias were scrutinised by constructing funnel plots for each pairwise comparison, predicated on estimates derived from direct evidence.

Random-effects network meta-analysis was performed using the restricted maximum likelihood (REML) method to account for between-study heterogeneity and to derive pooled effect sizes (42). The REML approach was preferred over standard maximum likelihood estimators owing to its capacity to yield less biased and more conservative variance components in small-to-moderate networks. Between-study heterogeneity was quantified via *τ*^2^, with its magnitude categorised according to established methodological literature as follows: low (<0.04), low–moderate (0.04–0.16), moderate–high (0.16–0.36), and high (>0.36) (43–45). The model assumed a common *τ*^2^ across all treatment comparisons within the network to maintain model parsimony and prevent non-convergence in sparse loops by enforcing a uniform variance magnitude. Furthermore, to mathematically adjust for the statistical dependency inherent in multi-arm trials sharing a single control group, the correlation coefficient in the between-study covariance matrix was pre-specified at 0.5 to avoid artificial sample size inflation and control for type I error rates (43, 44, 46).

The consistency assumption within the network was appraised at both local and global levels. Initially, local consistency was assessed using a node-splitting approach to evaluate the coherence between direct and indirect evidence within each closed loop.

Subsequently, global consistency was examined via a design-by-treatment interaction model to detect potential inconsistency across the entire network (45). For continuous outcomes with missing data, standard deviations were imputed from other randomised controlled trials with comparable participant demographics and intervention characteristics to actively combat selection bias and preserve statistical power, rather than executing listwise deletion. This procedure was performed in strict accordance with the recommendations of the Cochrane Handbook for Systematic Reviews of Interventions.

Network geometry was visualised via network maps generated in Stata. The comparative hierarchy of exercise interventions was established based on the surface under the cumulative ranking curve (SUCRA). Within this framework, a higher SUCRA value indicates a greater probability that a specific intervention represents the optimal modality for improving arterial stiffness or endothelial function (47).

## 3 Results

### 3.1 Literature search and selection

A comprehensive systematic search of the specified databases yielded 1,621 records. Following deduplication in EndNote (*n* = 746), 875 unique citations were retained for initial screening. Systematic scrutiny of titles and abstracts resulted in the exclusion of 704 records that failed to meet the predefined eligibility criteria; these comprised 536 clearly irrelevant studies, 107 reviews, conference abstracts, or meta-analyses, and 61 animal-based studies. The remaining 171 articles underwent rigorous full-text assessment. During this phase, 124 studies were further excluded for the following reasons: absence of relevant outcomes regarding arterial stiffness or endothelial function (*n* = 63), ineligible participant populations (*n* = 28), data insufficiency or unavailability of the full text (*n* = 18), and mismatched outcome measures (*n* = 15). Ultimately, 47 RCTs fulfilled all eligibility criteria and were included in this quantitative network meta-analysis. By adopting a broad search strategy that included both English (*n* = 46) and Mandarin (*n* = 1) sources, we ensured a comprehensive evidence base, thereby minimizing potential language-related selection bias.

### 3.2 Characteristics of the included studies

Overall, 47 RCTs encompassing 1,995 participants across 13 countries were identified for inclusion. Trial-specific sample sizes ranged from 20 to 114 participants, with intervention durations spanning 8–52 weeks. The pooled study population had a mean age of 65.80 years (SD 4.77), with a predominance of individuals aged 60–70 years.Regarding gender distribution, 636 (31.88%) participants were men and 1,359 (68.12%) were women (Supplementary Appendix 2, Table S2.1). At baseline, participant characteristics, including a mean BMI of 25.51 kg/m^2^ (SD 3.15), were well balanced across all study arms. Baseline vascular profiles revealed a mean flow-mediated dilation (FMD) of 5.06% (SD 2.04), a baPWV of 15.59 m/s (SD 2.13), and a cfPWV of 10.23 m/s (SD 1.82). Interventions consisted of seven distinct exercise modalities: MBE, SE, RT, WBV, AT, WT, and CT. The CON primarily maintained sedentary lifestyles. Most exercise protocols were prescribed at a frequency of 3–5 sessions per week, with session durations typically ranging from 30 to 60 min. Comprehensive characteristics of all included trials are provided in Supplementary Appendix 2.

### 3.3 Methodological quality and risk of bias

Methodological Quality and Risk of Bias: Detailed risk-of-bias assessments for each trial are provided in Supplementary Appendix 4. The methodological quality of the 47 RCTs was rigorously appraised using the RoB 2 tool, with results summarised as follows:

Regarding the randomisation process, 37 trials (79%) were judged to be at low risk of bias, while nine (19%) raised “some concerns.” In the domain of deviations from intended interventions, only three trials (6%) achieved a low-risk rating, with 44 (94%) classified as having “some concerns.” Notably, all 47 trials (100%) were rated as low risk for both missing outcome data and measurement of the outcome. For selection of the reported result, 41 trials (87%) were at low risk, while the remaining six (13%) were flagged for “some concerns.”In the overall bias assessment, three trials (6%) exhibited low risk across all domains, 42 (89%) were categorised as having “some concerns,” and two (5%) were judged to be at high risk. Primary methodological limitations were characterised by suboptimal reporting of blinding procedures (pertaining to participants, personnel, and outcome assessors) and incomplete documentation of attrition or dropout rates.

Consistency, Heterogeneity, and Certainty of Evidence: Nonetheless, assessments of coherence between direct and indirect evidence demonstrated no significant statistical inconsistency across the majority of comparisons; this finding was further corroborated by the node-splitting approach, which indicated robust local consistency. Although no substantial global heterogeneity was identified, analysis of *τ*^2^ values suggested that the magnitude of heterogeneity was moderate-to-high across most networks (Supplementary Appendix 5). According to the CINeMA framework, high confidence was observed in only a minority of pairwise comparisons, whereas the majority were appraised as having low-to-moderate certainty (Supplementary Appendix 9). Critically, the transitivity assumption was satisfied across all networks, thereby ensuring the validity of indirect comparisons. Finally, visual inspection of comparison-adjusted funnel plots yielded no evidence of overt publication bias (Supplementary Appendix 10).

### 3.4 Network meta-analysis of flow-mediated dilation

**Figure 2:**
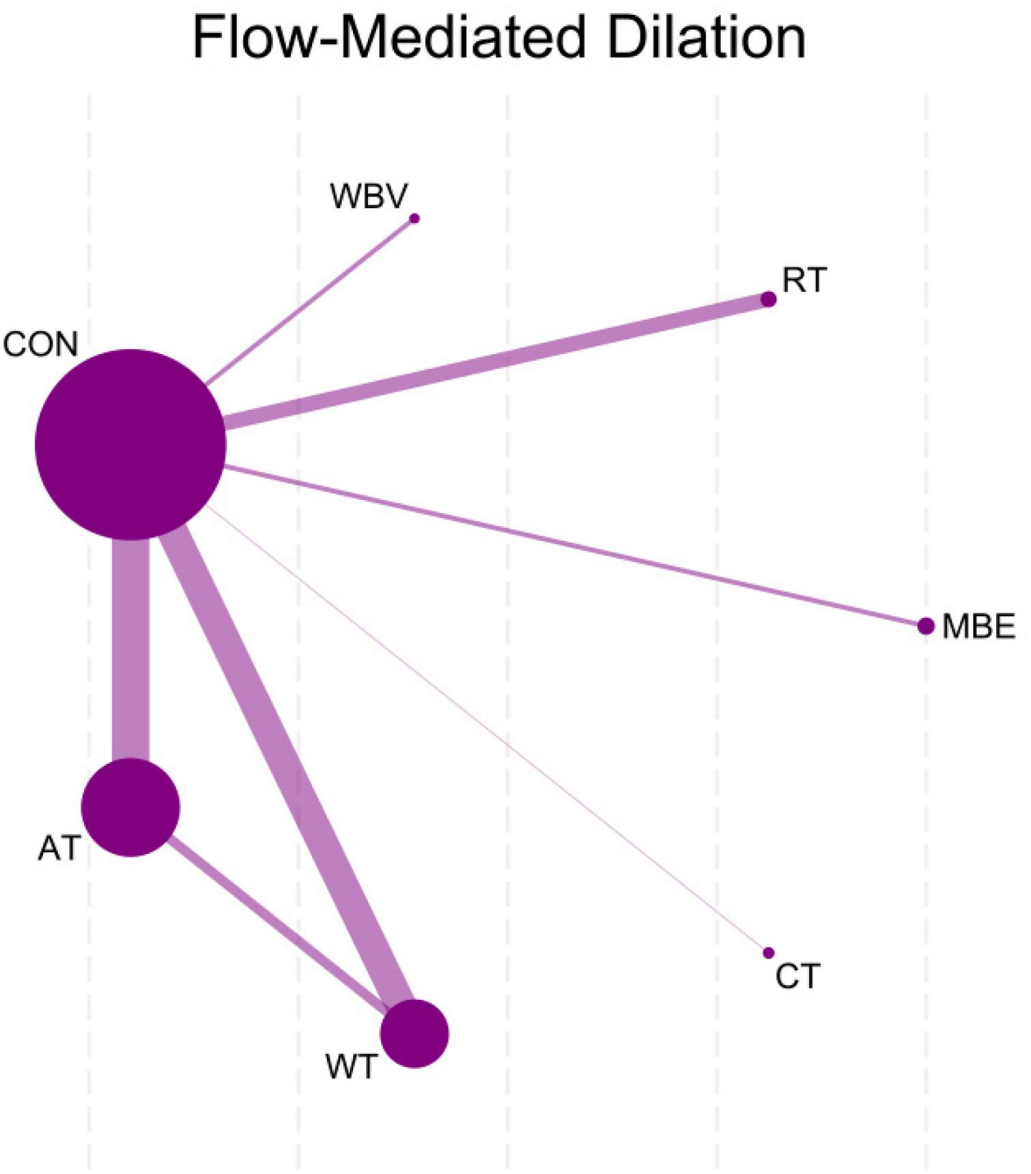
Network geometry for FMD. The diameter of each node is proportional to the total number of participants randomised to each intervention modality; The thickness of the connecting lines (edges) corresponds to the number of direct head-to-head trials between interventions.The structural matrix of the evidence base was mapped via network geometry (Figure 2A), which demonstrated comprehensive network connectivity predominantly anchored by the CON node. The presence of a closed loop among CON, AT, and WT structurally enabled both direct and indirect comparisons, thereby enhancing the statistical stability of the subsequent global consistency model. AT, aerobic training; CON, non-exercise control; CT, combined training; MBE, mind–body exercise; RT, resistance training; WBV, whole-body vibration; WT, walking training.

**Figure 3:**
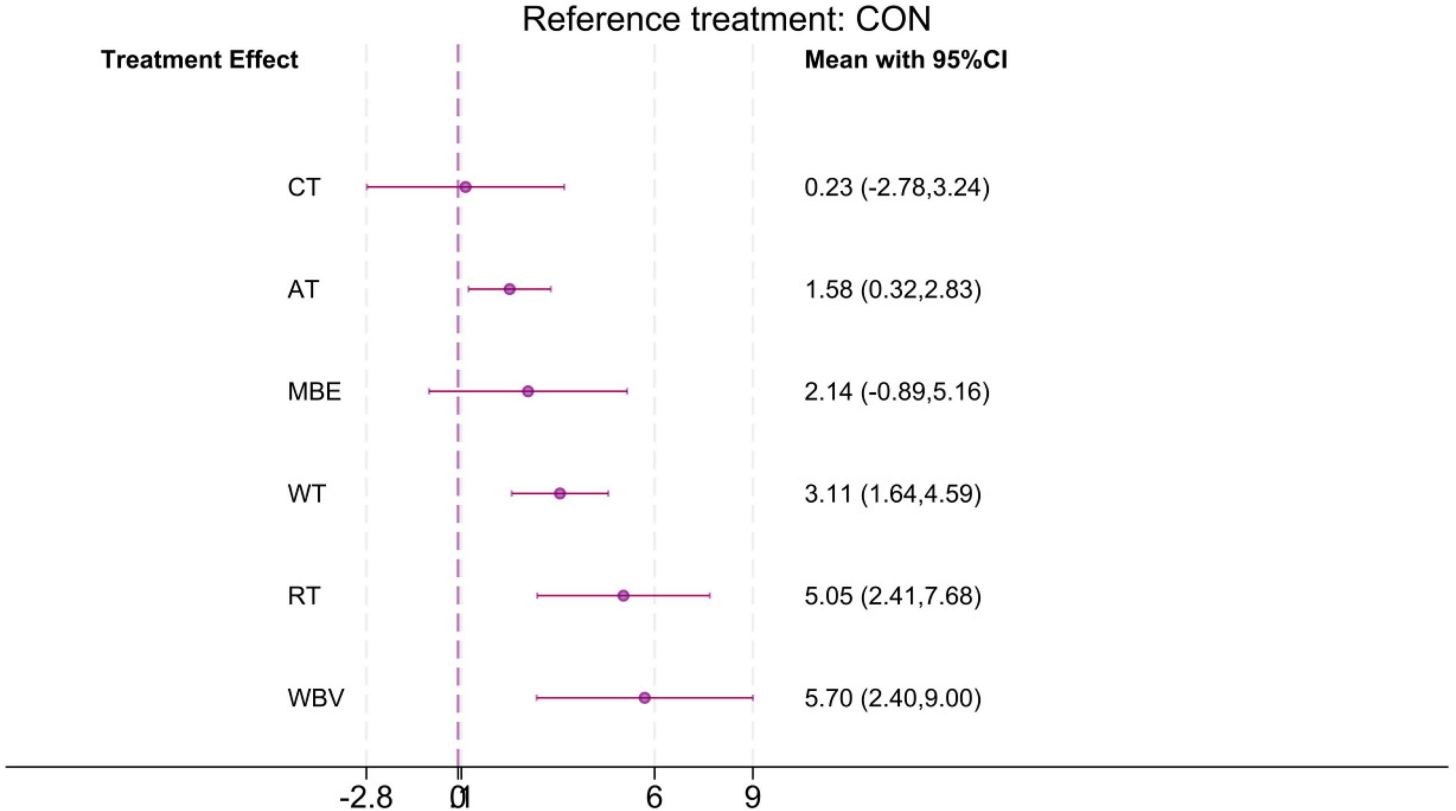
Forest plot of the network meta-analysis illustrating the relative effectiveness of six exercise intervention modalities vs. CON for the improvement of FMD. The plot presents the pooled mean differences (MDs) with 95% confidence intervals (CIs) for each intervention modality relative to the CON.

This network meta-analysis, encompassing 47 RCTs and eight distinct intervention modalities involving 1,995 participants, evaluated improvements in endothelial function and arterial stiffness in older adults. Regarding FMD (Figure 2), forest plot analysis (Figure 3) demonstrated that although all exercise regimens exhibited a trend towards amelioration compared with CON, only four attained statistical significance. Specifically, WBV (MD＝5.7,95% CI 2.4–9.0,SUCRA 91%) exhibited the greatest numerical mean difference for increasing FMD, followed sequentially by RT and WT (Figure 3). Crucially, cardiovascular epidemiological meta-analyses suggest that every 1% chronic improvement in brachial FMD is coupled with a significant 8% to 13% reduction in the risk of future cardiovascular events, indicating that the magnitude of amelioration identified across these leading modalities translates into a highly substantive reduction in clinical cardiovascular risk (48). League table estimates corroborated the primary ranking results, showing consistent effect directions across most head-to-head comparisons (Supplementary Appendix 8, Table S8.1). Notably, both WBV and RT showed greater FMD improvements than CT and AT. No significant differences were identified in the remaining comparisons, suggesting that efficacy differentials between certain exercise modalities may be marginal. According to the CINeMA framework, the overall certainty of evidence for FMD-related outcomes was predominantly appraised as moderate-to-high (Supplementary Appendix 9, Figure 9.2).

To further scrutinise the potential heterogeneity driven by baseline vascular health conditions, subgroup analyses were performed based on participant cohorts (Supplementary Appendix 11, Figure S11.1). Within the physiological vascular ageing cohort, the data indicated that statistical differences in FMD relative to CON were exclusively sustained following WBV and RT (Supplementary Figure S11.1A). In contrast, significant improvements vs. CON were uniquely observed after WT and AT within the cohort presenting with pathological endothelial dysfunction (Supplementary Figure S11.1B).

### 3.5 Network meta-analysis of carotid-femoral pulse wave velocity

**Figure 4:**
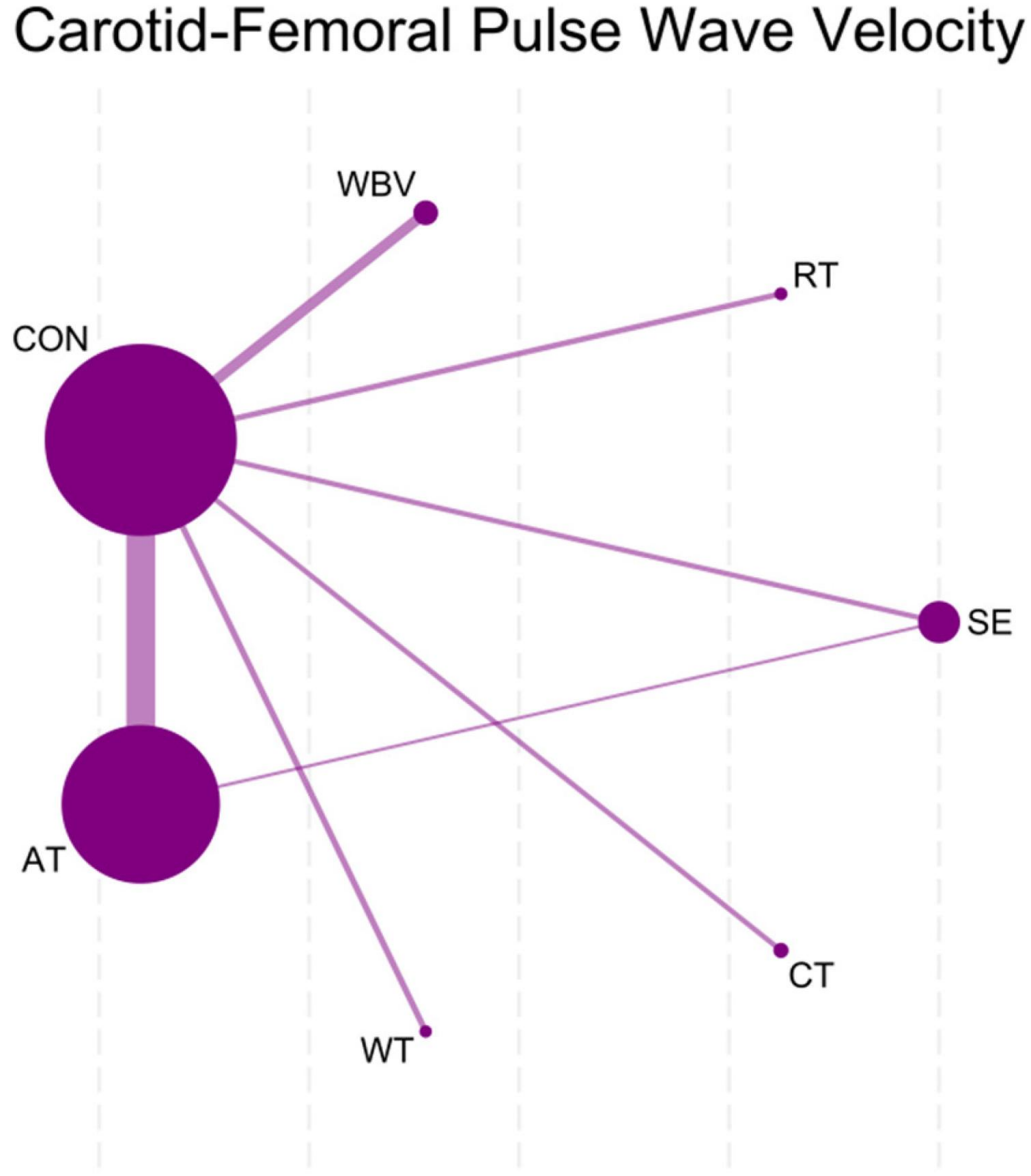
Network geometry for cfPWV. the diameter of each node is proportional to the total number of participants randomised to each intervention modality; the thickness of the connecting lines (edges) corresponds to the number of direct head-to-head trials between interventions. AT, aerobic training; CON, non-exercise control; CT, combined training; RT, resistance training; SE, stretching exercise; WBV, whole-body vibration; WT, walking training.

**Figure 5:**
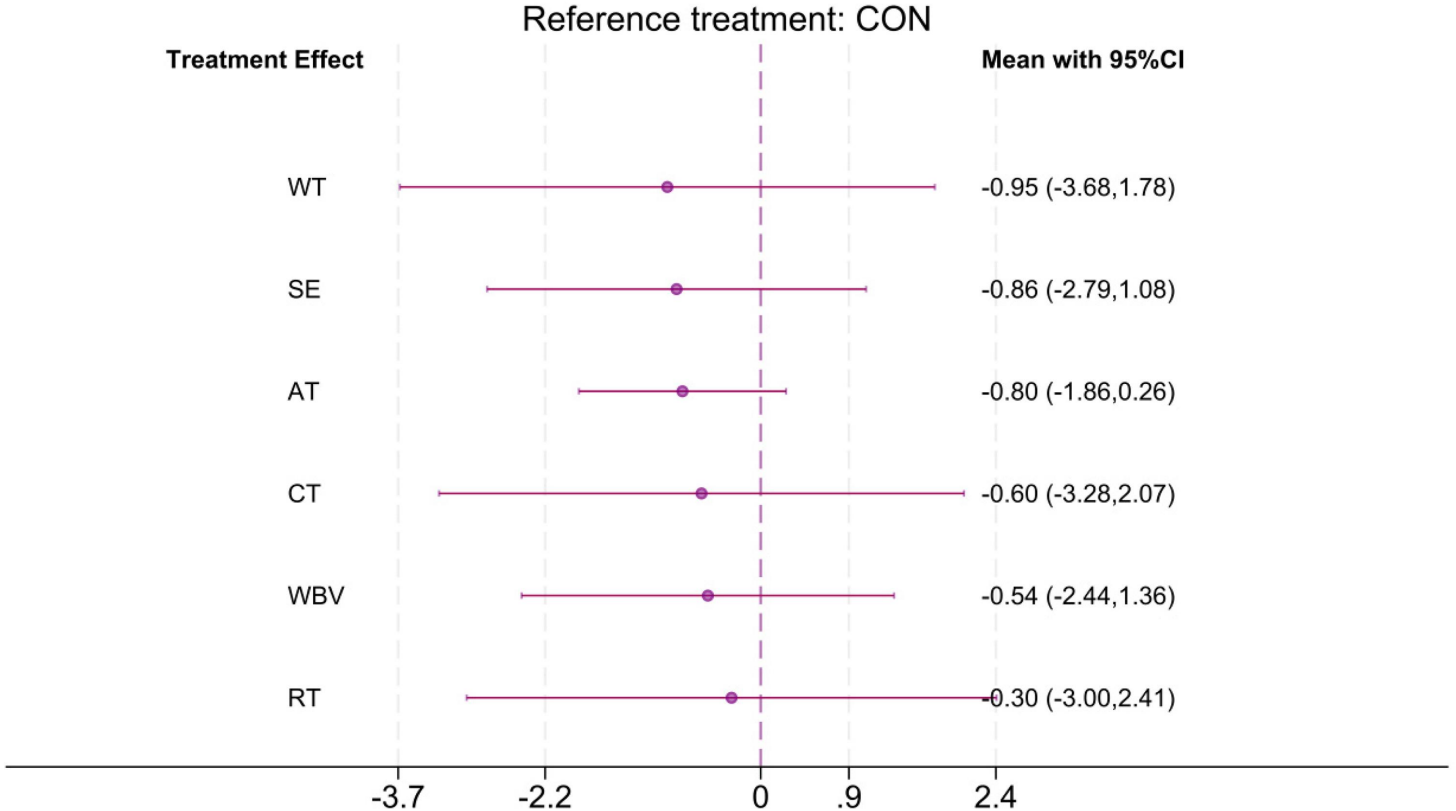
Forest plot of the network meta-analysis illustrating the relative effectiveness of six intervention modalities vs. CON for the reduction of cfPWV. The plot presents the SMDs with 95% CIs for each intervention modality relative to the CON.

Regarding the alteration in cfPWV (Figure 4), the primary network analysis suggested an overall downward trend across all exercise modalities compared with the control group (CON).However, none of these global reductions achieved statistical significance (Figure 5). This lack of definitive superiority was further corroborated by the pairwise comparisons in the league table (Supplementary Appendix 8 Table S8.2), indicating that within the unselected overall cohort, no single exercise modality demonstrated a statistically verifiable advantage over another in mitigating arterial stiffness.When evaluating probability-based rankings via the surface under the cumulative ranking curve (SUCRA) (Supplementary Appendix 7, Table S7.2), SE, AT, and WT shared the identical and highest probability cumulative score (SUCRA: 60.5%). Crucially, because these cumulative ranking curves are derived from non-significant network estimates, these metrics must be interpreted strictly as numerical probabilities rather than evidence of definitive clinical superiority or established therapeutic hierarchy.

To further scrutinise whether baseline vascular health status modifies these responses, subgroup analyses were executed based on cohorts with distinct physiological profiles (Supplementary Appendix 11, Figure S11.2). In the physiological vascular ageing cohort, a statistically significant reduction in cfPWV relative to CON was exclusively sustained following AT (Supplementary Figure S11.2A), a magnitude that represents a clinically meaningful deceleration of arterial structural stiffening. In contrast, within the cohort presenting with pathological endothelial dysfunction, significant improvements vs. CON were uniquely observed after SE and WT (Supplementary Figure S11.2B). These distinct sub-population findings suggest that while universal exercise recommendations lack statistical support for cfPWV reduction, precision-oriented exercise strategies tailored to baseline vascular pathology may yield verifiable clinical benefits.

### 3.6 Network meta-analysis of brachial-ankle pulse wave velocity

**Figure 6:**
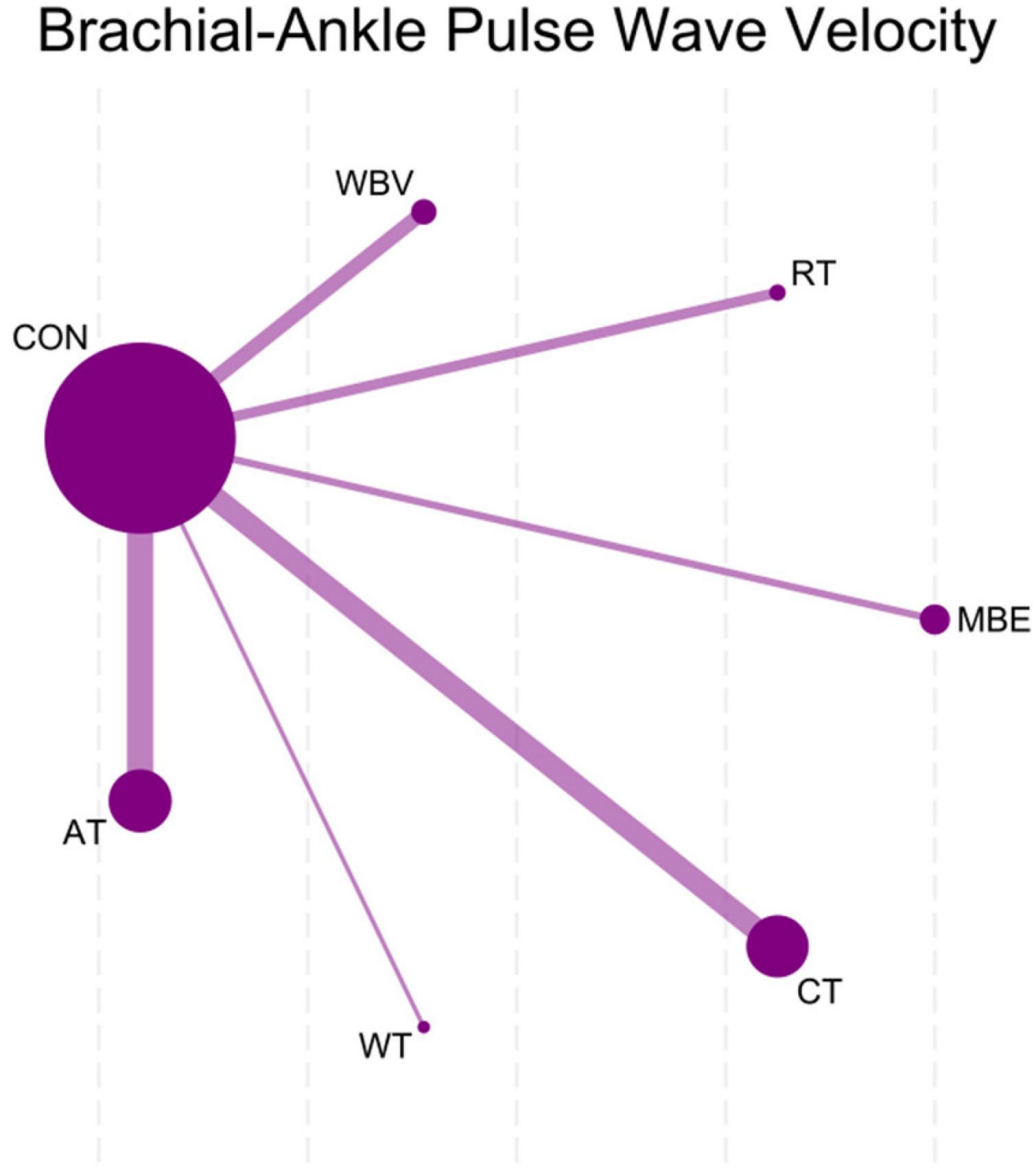
Network geometry for baPWV. the diameter of each node is proportional to the total number of participants randomised to each intervention modality; the thickness of the connecting lines (edges) corresponds to the number of direct head-to-head trials between interventions. AT, aerobic training; CON, non-exercise control; CT, combined training; MBE, mind–body exercise; RT, resistance training; WBV, whole-body vibration; WT, walking training.

**Figure 7:**
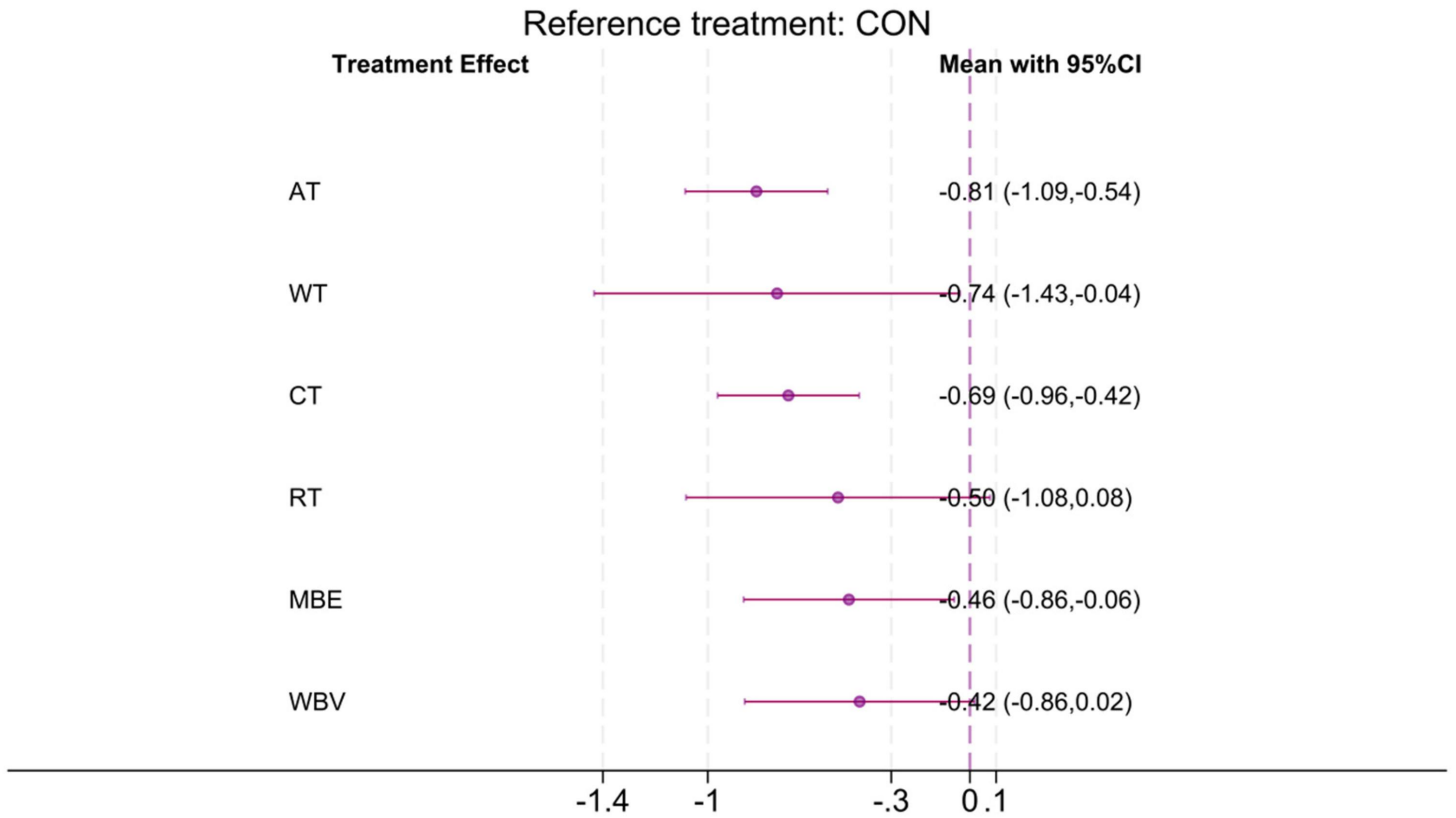
Forest plot of the network meta-analysis illustrating the relative effectiveness of six exercise intervention modalities vs. CON for the reduction of baPWV. The plot presents the SMDs with 95% CIs for each intervention modality relative to the CON.

Regarding the reduction in baPWV (Figure 6), forest plot analysis (Figure 7) demonstrated that although all exercise regimens exhibited a trend towards amelioration compared with CON, four attained statistical significance.Specifically, AT(SMD=–0.81,95% CI–1.09 to −0.54,SUCRA 83.3%) induced the largest standardized effect size in reducing baPWV, closely followed sequentially by WT, CT, and MBE (Figure 7). According to evidence from prospective epidemiological cohort studies, a sustained reduction of this magnitude translates into a 14.6% to 26.3% decrease in the risk of future total cardiovascular events, highlighting the profound translational impact of these interventions (49).This hierarchical pattern of clinical efficacy was broadly aligned with their respective cumulative ranking probabilities (Supplementary Appendix 7, Table S7.3). However, these SUCRA rankings reflect cumulative probability distributions rather than definitive clinical superiority. League table estimates corroborated the primary ranking results, showing consistent effect directions, though no statistically significant differences were identified across any head-to-head comparisons (Supplementary Appendix 8, Table S8.3), suggesting that efficacy differentials between these active exercise modalities may be marginal. According to the CINeMA framework, the overall certainty of evidence for baPWV-related outcomes was predominantly appraised as moderate (Supplementary Appendix 9, Fig 9.6).

To further scrutinise the potential heterogeneity driven by baseline vascular health conditions, subgroup analyses were performed for baPWV based on participant cohorts (Supplementary Appendix 11, Figure S11.3). Within the physiological vascular ageing cohort, the data indicated that statistical reductions in baPWV relative to CON were exclusively sustained following CT and AT (Supplementary Figure S11.3A).In contrast, significant reductions vs. CON were uniquely observed after WT, CT, and AT within the cohort presenting with pathological endothelial dysfunction (Supplementary Figure S11.3B).

### 3.7 Network meta-analysis of systolic blood pressure

**Figure 8:**
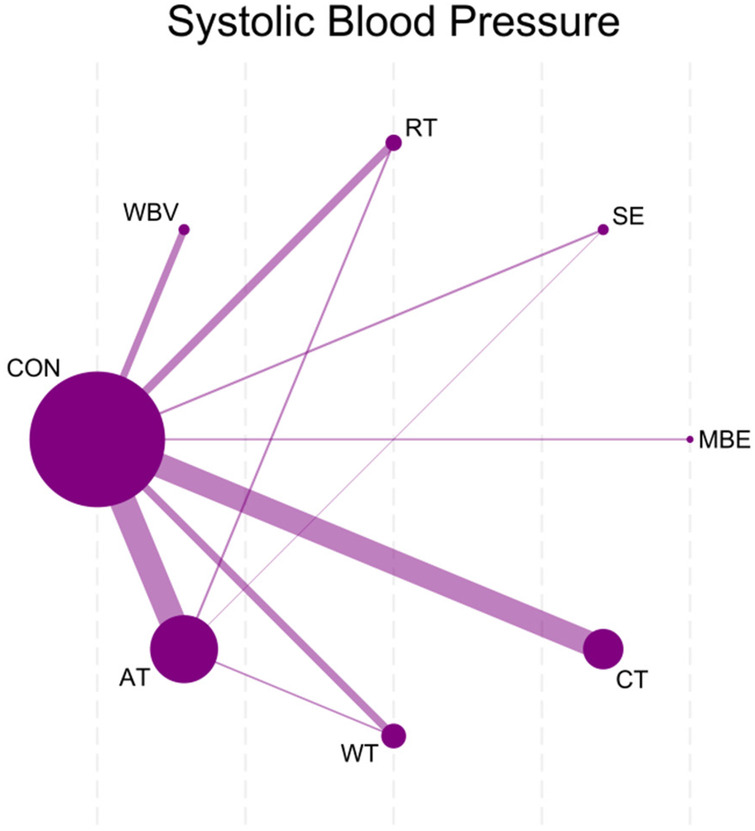
Network geometry for SBP. The diameter of each node is proportional to the total number of participants randomised to each intervention modality; the thickness of the connecting lines (edges) corresponds to the number of direct head-to-head trials between interventions. AT, aerobic training; CON, non-exercise control; CT, combined training; MBE, mind–body exercise; RT, resistance training; SE, stretching exercise; WBV, whole-body vibration; WT, walking training.

**Figure 9:**
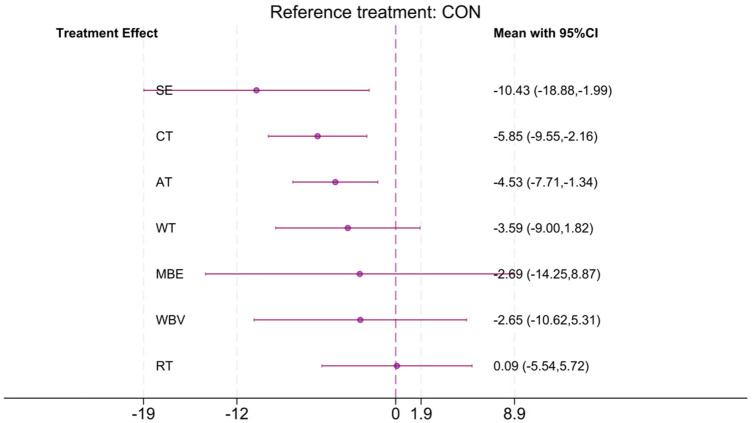
Forest plot of the network meta-analysis illustrating the relative effectiveness of seven exercise intervention modalities vs. CON for the reduction of SBP. The plot presents the pooled MDs with 95% CIs for each intervention modality relative to the CON group.

Regarding SBP (Figure 8), forest plot analysis (Figure 9) demonstrated that although most exercise regimens exhibited a non-significant trend towards blood pressure reduction compared with CON, only three attained statistical significance. Specifically, SE (MD = –10.43; 95% CI −18.88 to −1.99; SUCRA = 91.8%) demonstrated a statistically significant reduction in SBP, followed by CT and AT. According to established epidemiological and collaborative individual participant-level meta-analyses, a 10 mmHg decrease in SBP translates directly to a 20% relative risk reduction in major cardiovascular events, a 17% reduction in coronary heart disease, a 27% reduction in stroke, and a 13% reduction in all-cause mortality (50).In terms of cumulative ranking probabilities, SUCRA values (Supplementary Appendix 7, Table 7.7) indicated that SE had the highest probability of being ranked best (SUCRA = 91.8%), followed sequentially by CT (72.4%) and AT (60.2%). However, these SUCRA values reflect cumulative probability distributions rather than definitive clinical superiority, particularly among the remaining modalities (WT, MBE, WBV, and RT) where rankings are heavily driven by non-significant differences. League table estimates mirrored these primary findings, demonstrating consistent effect directions across most head-to-head comparisons (Supplementary Appendix 8, Table S8.7). Notably, while SE showed statistically greater SBP reductions than several other active interventions, no significant differences were identified in the remaining comparisons. This lack of statistical differentiation reinforces that efficacy differentials between certain exercise modalities may be marginal and largely overlapping. According to the CINeMA framework, the overall certainty of evidence for SBP-related outcomes was predominantly appraised as moderate-to-high (Supplementary Appendix 9, Figure 9.8).

To further scrutinise the potential heterogeneity driven by baseline vascular health conditions, subgroup analyses for SBP were performed based on participant cohorts (Supplementary Appendix 11, Figure S11.4). Within the physiological vascular ageing cohort, the data indicated that statistical differences in SBP relative to CON were exclusively sustained following CT, AT, and RT (Supplementary Figure S11.4A). Conversely, no significant improvements vs. CON were observed after any exercise intervention within the cohort presenting with pathological endothelial dysfunction (Supplementary Figure S11.4B), suggesting that the therapeutic efficacy of exercise on blood pressure may be attenuated or influenced by inter-individual variability in populations with established vascular pathology.

### 3.8 Network meta-analysis of diastolic blood pressure

**Figure 10:**
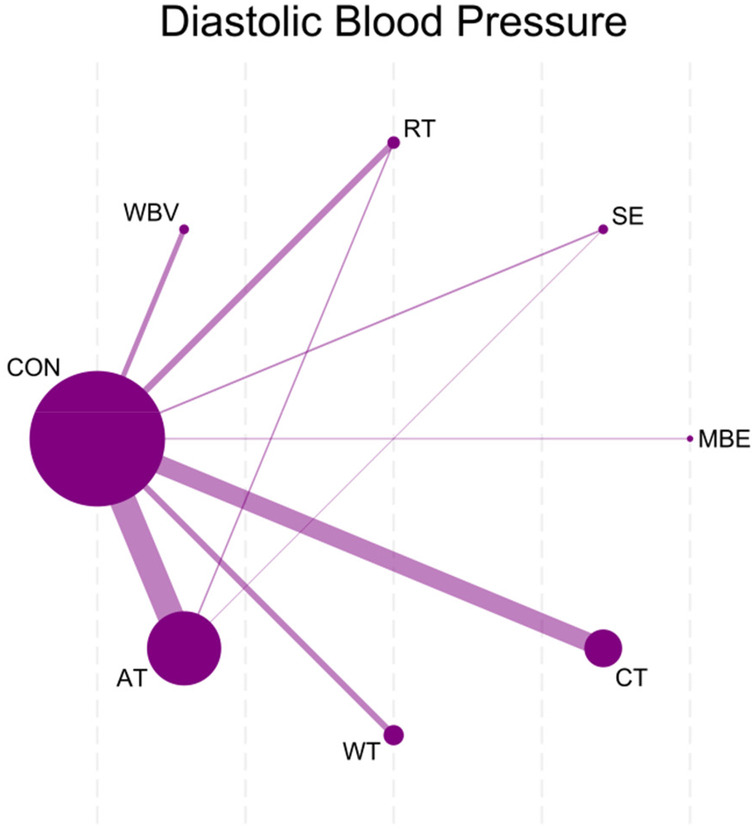
Network geometry for DBP. The diameter of each node is proportional to the total number of participants randomised to each intervention modality; the thickness of the connecting lines (edges) corresponds to the number of direct head-to-head trials between interventions. AT, aerobic training; CON, non-exercise control; CT, combined training; MBE, mind–body exercise; RT, resistance training; SE, stretching exercise; WBV, whole-body vibration; WT, walking training.

**Figure 11:**
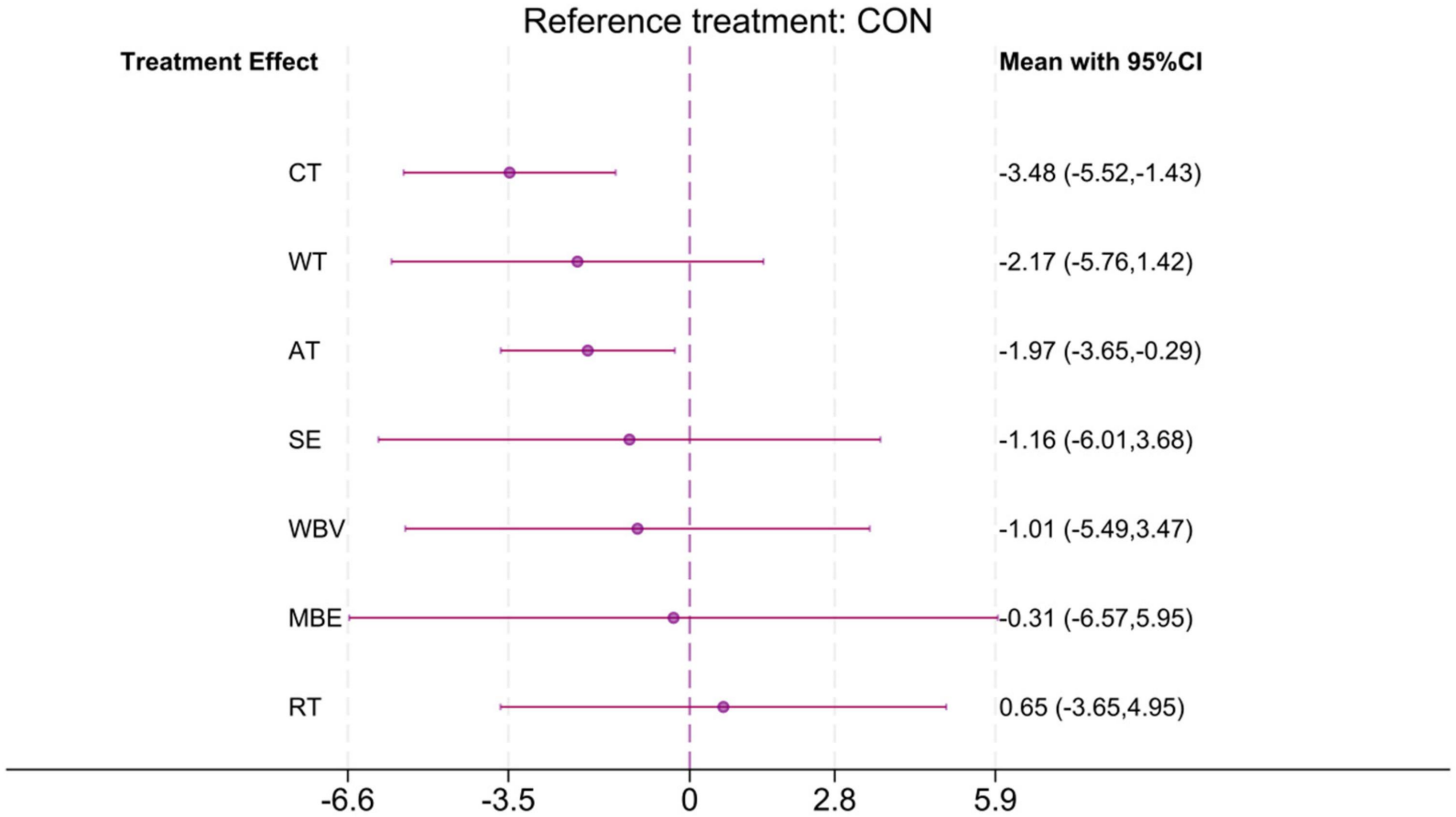
Forest plot of the network meta-analysis illustrating the relative effectiveness of seven exercise intervention modalities vs. non-exercise controls for the reduction of DBP. The plot presents the pooled MDs with 95% CIs for each intervention modality relative to the CON.

With respect to the reduction in DBP (Figure 10), forest plot analysis (Figure 11) demonstrated that while most intervention modalities exhibited a non-significant trend towards blood pressure reduction compared with CON, only two reached statistical significance. Specifically, CT (MD = –3.48; 95% CI −5.52 to −1.43; SUCRA = 86.1%), demonstrated a statistically significant reduction in DBP, followed by AT. Cardiovascular epidemiological models indicate that even a modest 2mmHg population-wide reduction in usual DBP is associated with a 15% lower risk of stroke, a 6% to 9% lower risk of coronary heart disease (CHD), and a 17% reduction in the overall prevalence of hypertension (51). In terms of cumulative ranking probabilities, SUCRA values (Supplementary Appendix 7, Table 7.8) indicated that CT had the highest probability of being ranked best (SUCRA = 86.1%), followed by WT (64.7%) and AT (63.1%). Crucially, these SUCRA rankings reflect cumulative probability distributions rather than definitive clinical superiority; For instance, WT achieved a higher SUCRA ranking than AT purely based on mathematical probability, despite failing to attain statistical significance against CON. League table estimates mirrored these primary findings, demonstrating consistent effect directions and revealing no statistically significant differences across any active pairwise comparisons (Supplementary Appendix 8, Table S8.8). This lack of statistical differentiation reinforces that efficacy differentials between certain exercise modalities may be marginal and largely overlapping. According to the CINeMA framework, the overall certainty of evidence for DBP-related outcomes was predominantly appraised as moderate-to-high (Supplementary Appendix 9, Figure 9.10).

To further scrutinise the potential heterogeneity driven by baseline vascular health conditions, subgroup analyses for DBP were performed based on participant cohorts (Supplementary Figure S11.5).Within the physiological vascular ageing cohort, the data indicated that statistical differences in DBP relative to CON were exclusively sustained following CT (Supplementary Figure S11.5A). Conversely, statistically significant improvements vs. CON were re-established after both CT and AT within the cohort presenting with pathological endothelial dysfunction (Supplementary Figure S11.5B).

## 4 Discussion

### 4.1 Primary findings

This network meta-analysis provided a comprehensive evaluation of the comparative effectiveness of seven exercise modalities on arterial stiffness and endothelial function in older adults. By synthesising evidence from 47 RCTs encompassing 1,995 participants, our findings indicate that while exercise interventions generally confer vascular benefits in older populations, their therapeutic profiles vary significantly across distinct physiological indices.

This network meta-analysis highlights the nuanced and variable impacts of diverse exercise modalities on vascular health in older adults, underscoring that therapeutic responses are heavily conditioned by both the specific vascular target and baseline clinical status. Methodologically, our findings demand highly cautious interpretation; although specific modalities such as WBV and RT scored favorably in mean rankings for endothelial function (FMD), and AT or WT for peripheral arterial stiffness (baPWV), the extensive overlap in confidence intervals precluded any definitive claims of head-to-head clinical superiority. Crucially, no exercise modality yielded statistically significant improvements in central arterial stiffness (cfPWV), while the effects on hemodynamic outcomes remained highly heterogeneous. Exploratory subgroup analyses suggested that baseline vascular profiles may modulate these responses, showing divergent trends between individuals with physiological vascular ageing and those with established pathological endothelial dysfunction. However, given the compromised statistical power and expanded uncertainty inherent in network sub-cohorts, these stratifications should be viewed as purely hypothesis-generating rather than confirmatory. Collectively, the statistical evidence does not support the prescription of a single “most effective” modality; instead, it indicates that exercise-induced vascular benefits in older populations are modest, non-inferior across active interventions, and require individualized prescription based on baseline cardiovascular risk.

### 4.2 Comparison with previous research

Our results substantiate and extend the existing evidence landscape regarding exercise-induced vascular adaptations. Congruent with previous meta-analyses (52–56), the present study reaffirms the role of AT and WT in reducing peripheral arterial stiffness and improving endothelial function. Furthermore, by synthesizing a broader and more granular network of evidence, we noted that RT and WBV exhibited descriptive trends toward higher mean rankings in FMD improvement; However, given the widely overlapping confidence intervals across these modalities, these observations provide additional contextual clarity to previous ranking variations rather than demonstrating definitive clinical superiority (57). Notably, the minor discrepancies in statistical significance regarding cfPWV between our findings and previous reports (55) are likely attributable to the implementation of more stringent inclusion criteria and the adoption of a more conservative estimation model that properly reflects the statistically non-significant differences across network nodes. Collectively, these findings underscore the clinical relevance of a nuanced, multimodal framework specifically tailored to the management of age-related vascular disease, rather than relying solely on a generic, aerobic-centric perspective, while strictly acknowledging that individual therapeutic responses remain modest and non-inferior.

### 4.3 Mechanisms underlying the enhancement of FMD by WBV and RT

The demonstrated superiority of WBV and RT in enhancing FMD among older adults underscores the pivotal role of diversified mechanical stimuli in reversing endothelial senescence. WBV exerts its vasculoprotective effects via high-frequency, low-amplitude oscillatory shear stress (58), which serves as a potent mechanical trigger to directly activate mechanosensitive ion channels and catalyse the phosphorylation of endothelial nitric oxide synthase (eNOS) (59). This “vascular massage” effect potentially facilitates the recuperation of endothelial glycocalyx integrity or bypasses age-associated structural impairment (60), thereby providing a highly efficient intervention modality for frail older populations (61). Analogously, the substantial benefits derived from RT stem from a “hormetic response" (62) induced by intermittent ischemia–reperfusion cycles. Transient shear stress oscillations during resistance exercise, followed by subsequent reactive hyperaemia, prompt the endothelial system to upregulate antioxidant defence mechanisms. This process consequently enhances nitric oxide bioavailability (62) and stimulates vascular endothelial growth factor (VEGF)-mediated angiogenesis (63).

### 4.4 Mechanisms underlying the reduction of baPWV following AT and WT

The present study demonstrates that interventions primarily centered on AT and WT yield a substantial reduction in baPWV. The efficacy of AT in attenuating baPWV is largely attributable to the acute and chronic modulation of vasomotor tone; specifically, rhythmic AT elicits the localized release of metabolic vasodilators, such as adenosine, lactate, and potassium ions, while concurrently facilitating nitric oxide (NO)-mediated relaxation of the medial smooth muscle (64). Furthermore, WT underscores the pivotal role of the “lower-limb muscle pump” in delivering targeted hemodynamic stimuli, which directly elevates shear stress and flow pulsatility within the vascular beds of the lower extremities (54). This site-specific mechanotransduction elucidates the superior clinical outcomes associated with walking in modulating peripheral vascular segments compared with those observed in exercises limited to the upper limbs or static exertion. These findings suggest that exercise-induced improvements in arterial stiffness among older adults are predominantly mediated by functional regulatory adaptations rather than permanent structural remodeling (65).

### 4.5 Internal mechanisms underlying the structural irreversibility of cfPWV

The failure of cfPWV to demonstrate significant attenuation underscores a critical physiological bottleneck: the structural irreversibility of aortic aging within the temporal constraints of conventional clinical trials. In older cohorts, central arterial stiffness is primarily mediated by the degradation of elastic lamellae, pathological collagen cross-linking, and medial calcification (1). These structural remodeling processes appear relatively refractory to the functional modulation of smooth muscle tone, which serves as the principal target of short-term (8–12 week) exercise interventions, thereby contrasting with the more plastic adaptations observed in muscular arteries (66). Critically, this lack of improvement yields profound clinical and prognostic implications. It indicates that lifestyle or exercise interventions targeting central macrovascular compliance in geriatric populations demand a substantially extended therapeutic window, or must be initiated at an earlier stage of vascular aging before advanced elastolysis becomes structurally fixed. Furthermore, from a diagnostic perspective, cfPWV exhibits pronounced isobaric dependence; in the absence of a substantial reduction in mean arterial pressure, the aorta remains in a distended, high-pressure state where elevated stiffness may mask underlying improvements in intrinsic vascular compliance (67). This biomechanical constraint implies that the conventional reliance on cfPWV as a standalone metric may inadvertently precipitate a ‘false negative’ appraisal of short-term vascular benefits, failing to capture subtle cellular or microstructural amelioration within the aortic wall. Consequently, these findings highlight the clinical imperative for a dual-metric evaluation framework to effectively delineate functional from structural vascular health in aging populations, ensuring that therapeutic efficacy is not prematurely dismissed due to the structural inertia of central conduit arteries.

### 4.6 Comparative mechanistic pathways of SE on SBP and CT on diastolic blood pressure regulation

As a localized, low-intensity mechanical stimulus, SE subjects peripheral vasculature to longitudinal tensile strain, which directly gates mechanosensitive channels on the endothelial cell membrane. This mechanotransduction cascades into the phosphorylation of endothelial nitric oxide synthase (eNOS), upping the bioavailability of nitric oxide (NO) to trigger vascular smooth muscle relaxation and subsequent decreases in peripheral resistance; concurrently, SE attenuates sympathetic vasomotor tone (68). This systemic-functional orchestration enhances peripheral muscular arterial compliance (Cm) and decelerates the velocity of the retrograde reflected pressure wave, ensuring its return during diastole to effectively blunt the peak SBP (69). Conversely, combined training (CT) synergistically integrates the distinct pathways of aerobic (AT) and resistance training (RT). The AT component imposes continuous, laminar shear stress to promote endothelial NO release, whereas the RT component drives reactive hyperemia to stimulate microvascular remodeling and capillary angiogenesis, thereby expanding the total cross-sectional area of the peripheral capillary bed (70). These coupled structural and functional adaptations systematically lower total peripheral resistance (TPR). Characterized by the Windkessel model, this reduction in TPR shortens the diastolic pressure decay time constant (*τ*= TPR·C), accelerating diastolic pressure dissipation to significantly reduce diastolic blood pressure. Furthermore, CT mitigates renal and cardiac lipid peroxidation by over 50%, effectively preserving endothelial-dependent vasodilation through the scavenging of reactive oxygen species (ROS) (71).

## 5 Strengths and limitations

This study establishes a comprehensive evaluative framework by simultaneously integrating macrovascular indices (baPWV and cfPWV), microvascular endothelial responsiveness (FMD), and systemic hemodynamic profiles (SBP and DBP). Rather than overinterpreting absolute rankings, this multifaceted network accounts for the site-specific nature of vascular adaptations, showing that WBV and RT ranked highest in cumulative probabilities for FMD, and SE was associated with greater improvement in blood pressure modulation. Furthermore, by rigorously applying the CINeMA framework to grade the certainty of evidence, we have established a transparent and robust hierarchy for exercise intervention protocols. This systematic approach offers substantive refinements to the clinical evidence base essential for the development of individualized, precision exercise strategies for aging populations.

Nonetheless, these findings must be contextualized within the scope of several inherent limitations. First, while our cohort's female predominance (68.12%) reflects established epidemiological trends in geriatric vascular research, the baseline homogeneity across intervention arms and the sex-conserved physiological mechanisms of exercise-induced vascular remodeling ensure our comparative effectiveness findings remain robust. Second, the relative dearth of evidence for specific exercise modalities yielded imprecise estimates, as manifested by widened confidence intervals for certain indirect comparisons, which consequently tempered the certainty of our evidence-based rankings. Third, the extensive overlap in confidence intervals across active interventions precludes definitive claims of head-to-head clinical superiority. Finally, although a rigorous random-effects model was employed to mitigate clinical heterogeneity, the absence of data regarding long-term adherence and post-intervention sustainability, particularly the clinical “washout effect,” underscores that these rankings represent exploratory adaptations rather than long-term confirmatory efficacy. Future prospective longitudinal research remains essential to confirm the durability of the vascular benefits observed across these exercise modalities.

## 6 Conclusion

This network meta-analysis demonstrates that diverse exercise modalities exert nuanced and variable impacts on the pathophysiological hallmarks of vascular aging in older adults, with outcomes heavily conditioned by the specific vascular target. Methodologically, although certain modalities such as WBV and RT ranked highest in mean cumulative probabilities for FMD, and AT or WT for baPWV, the extensive overlap in confidence intervals precludes definitive claims of clinical superiority. Crucially, active interventions show statistical non-inferiority across modalities, and no exercise type yields significant improvements in central aortic stiffness (cfPWV). Furthermore, observed variations in SBP and DBP reductions remain exploratory, given the high heterogeneity and the hypothesis-generating nature of subgroup stratifications. Consequently, the statistical evidence does not support a uniform, single recommendation; instead, it underscores that exercise-induced vascular benefits are modest and necessitate individualized, precision-based prescription tailored to baseline cardiovascular risks and specific phenotypes. Future research must prioritize adequately powered, long-term trials to further characterize the dose-response relationships and durability of these adaptations.

## Data Availability

The datasets presented in this study can be found in online repositories. The names of the repository/repositories and accession number(s) can be found in the article/Supplementary Material.

## References

[1] Herzog MJ Müller P Lechner K Stiebler M Arndt P Kunz M. Arterial stiffness and vascular aging: mechanisms, prevention, and therapy. Signal Transduct Target Ther. (2025) 10(1):282. 10.1038/s41392-025-02346-0 40887468 PMC12399776

[2] Adhikari H Patel P Javvaji N Crabtree MJ Simon JN. Declining nitric oxide bioavailability in cardiovascular aging: mechanistic insights and emerging interventions. J Cardiovasc Aging. (2025) 5(4):20. 10.20517/jca.2025.14 41953454 PMC13055925

[3] Smith ER Tomlinson LA Ford ML McMahon LP Rajkumar C Holt SG. Elastin degradation is associated with progressive aortic stiffening and all-cause mortality in predialysis chronic kidney disease. Hypertension. (2012) 59(5):973–8. 10.1161/HYPERTENSIONAHA.111.187807 22411928

[4] Mitchell GF. Arterial stiffness in aging: does it have a place in clinical practice?: recent advances in hypertension. Hypertension. (2021) 77(3):768–80. 10.1161/HYPERTENSIONAHA.120.14515 33517682

[5] Sequí-Domínguez I Cavero-Redondo I Álvarez-Bueno C Pozuelo-Carrascosa DP Nuñez de Arenas-Arroyo S Martínez-Vizcaíno V. Accuracy of pulse wave velocity predicting cardiovascular and all-cause mortality. A systematic review and meta-analysis. J Clin Med. (2020) 9(7):2080. Published 2020 July 2. 10.3390/jcm9072080 32630671 PMC7408852

[6] Jones DW Ferdinand KC Taler SJ Johnson HM Shimbo D Abdalla M. 2025 AHA/ACC/AANP/AAPA/ABC/ACCP/ACPM/AGS/AMA/ASPC/NMA/PCNA/SGIM guideline for the prevention, detection, evaluation and management of high blood pressure in adults: a report of the American College of Cardiology/American Heart Association joint committee on clinical practice guidelines. Hypertension. (2025) 82(10):e212–316. 10.1161/HYP.0000000000000249 40811516

[7] Niiranen TJ Kalesan B Hamburg NM Benjamin EJ Mitchell GF Vasan RS. Relative contributions of arterial stiffness and hypertension to cardiovascular disease: the framingham heart study. J Am Heart Assoc. (2016) 5(11):e004271. 10.1161/JAHA.116.004271 27912210 PMC5210358

[8] Johns Hopkins Medicine. Polypharmacy in adults 60 and older. Available online at: https://www.hopkinsmedicine.org/health/wellness-and-prevention/polypharmacy-in-adults-60-and-older (Accessed December 18, 2025).

[9] American Heart Association. American Heart Association recommendations for physical activity in adults and kids. Accessed January 23, 2026. Available online at: https://www.heart.org/en/healthy-living/fitness/fitness-basics/aha-recs-for-physical-activity-infographic Accessed December 20, 2025.

[10] U.S. Department of Health and Human Services. *Physical Activity Guidelines for Americans*. 2nd ed. Washington, DC: U.S. Department of Health and Human Services (2018).

[11] Saz-Lara A Cavero-Redondo I Álvarez-Bueno C Notario-Pacheco B Reina-Gutiérrez S Sequí-Domínguez I. What type of physical exercise should be recommended for improving arterial stiffness on adult population? A network meta-analysis. Eur J Cardiovasc Nurs. (2021) 20(7):696–716. 10.1093/eurjcn/zvab022 33837399

[12] Tao X Chen Y Zhen K Ren S Lv Y Yu L. Effect of continuous aerobic exercise on endothelial function: a systematic review and meta-analysis of randomized controlled trials. Front Physiol. (2023) 14:1043108. 10.3389/fphys.2023.1043108 36846339 PMC9950521

[13] Lan YS Khong TK Yusof A. Effect of exercise on arterial stiffness in healthy young, middle-aged and older women: a systematic review. Nutrients. (2023) 15(2):308. 10.3390/nu15020308 36678179 PMC9867069

[14] TensioMed. Exercise and arterial stiffness. Available online at: https://tensiomed.com/exercise-and-arterial-stiffness/ (Accessed January 23, 2026).

[15] Aoyama A Yamaoka-Tojo M Obara S Shimizu E Fujiyoshi K Noda C. Acute effects of whole-body vibration training on endothelial function and cardiovascular response in elderly patients with cardiovascular disease. Int Heart J. (2019) 60(4):854–61. 10.1536/ihj.18-592 31257335

[16] Naci H Ioannidis JPA. Comparative effectiveness of exercise and drug interventions on mortality outcomes: metaepidemiological study. Br Med J. (2013) 347:f5577. 10.1136/bmj.f5577 24473061 PMC3788175

[17] Pelliccia A Sharma S Gati S Bäck Maria Börjesson M Caselli S. 2020 ESC guidelines on sports cardiology and exercise in patients with cardiovascular disease: the task force on sports cardiology and exercise in patients with cardiovascular disease of the European Society of Cardiology (ESC). Eur Heart J. (2021) 42(1):17–96. 10.1093/eurheartj/ehaa605 32860412

[18] Campbell A Grace F Ritchie L Beaumont A Sculthorpe N. Long-term aerobic exercise improves vascular function into old age: a systematic review, meta-analysis, and meta-regression of observational and interventional studies. Front Physiol. (2019) 10:31. 10.3389/fphys.2019.00031 30863313 PMC6399418

[19] Fuertes-Kenneally L Blasco-Peris C Casanova-Lizón A Baladzhaeva S Climent V Sarabia JM. Effects of high-intensity interval training on vascular function in patients with cardiovascular disease: a systematic review and meta-analysis. Front Physiol. (2023) 14:1196665. 10.3389/fphys.2023.1196665 37576344 PMC10413117

[20] Wu RS Zhang Y Yuan XW Yan X Fu XL. Comparative effectiveness of exercise interventions on arterial stiffness in individuals at risk for cardiovascular disease: a systematic review and network meta-analysis. Front Cardiovasc Med. (2025) 12:1489382. 10.3389/fcvm.2025.1489382 40083821 PMC11905979

[21] Page MJ McKenzie JE Bossuyt PM Boutron I Hoffmann TC Mulrow CD. The PRISMA 2020 statement: an updated guideline for reporting systematic reviews. Br Med J. (2021) 372:n71. Published 2021 March 29. 10.1136/bmj.n71 33782057 PMC8005924

[22] . Hutton B Salanti G Caldwell DM Chaimani A Schmid CH Cameron C. The PRISMA extension statement for reporting of systematic reviews incorporating network meta-analyses of health care interventions: checklist and explanations. Ann Intern Med. (2015) 162(11):777–84. 10.7326/M14-2385 26030634

[23] American College of Sports Medicine. In: Ozemek C Bonikowske AR Christle JW Gallo PM, editors. ACSM's Guidelines for Exercise Testing and Prescription. 12th ed. Philadelphia, PA: Wolters Kluwer (2026). p. 1–26, 148–174.

[24] Chodzko-Zajko WJ Proctor DN Fiatarone Singh MA Minson CT Nigg CR Salem GJ. American College of sports medicine position stand. Exercise and physical activity for older adults. Med Sci Sports Exerc. (2009) 41(7):1510–30. 10.1249/MSS.0b013e3181a0c95c 19516148

[25] Buttolph L Bruton AM Filbin P Wexler RS Gray O Mazure T. Effects of mind-body movement interventions for managing symptoms in people with multiple sclerosis: an overview of reviews. Curr Neurol Neurosci Rep. (2026) 26(1):10. 10.1007/s11910-025-01478-8 41619131 PMC12860761

[26] Pierce S Shah S. Prescribing Movement: Tool for Clinicians. Madison: Osher Center for Integrative Health, University of Wisconsin-Madison (2020). Available online at: https://www.fammed.wisc.edu/osher (Accessed May 28, 2026).

[27] Currier BS D'Souza AC Singh MAF Lowisz CV Rawson ES Schoenfeld BJ. American College of sports medicine position stand. Resistance training prescription for muscle function, hypertrophy, and physical performance in healthy adults: an overview of reviews. Med Sci Sports Exerc. (2026) 58(4):851–72. 10.1249/MSS.0000000000003897 41843416 PMC12965823

[28] van Heuvelen MJG Rittweger J Judex S Sañudo B Seixas A Fuermaier ABM. Reporting guidelines for whole-body vibration studies in humans, animals and cell cultures: a consensus statement from an international group of experts. Biology (Basel). (2021) 10(10):965. 10.3390/biology10100965 34681065 PMC8533415

[29] Wuestefeld A Fuermaier ABM Bernardo-Filho M da Cunha de Sá-Caputo D Rittweger J Schoenau E. Towards reporting guidelines of research using whole-body vibration as training or treatment scheme in human subjects—a delphi consensus study. PLoS One. (2020) 15(7):e0235905. 10.1371/journal.pone.0235905 32697809 PMC7375612

[30] Bushman BA, editor. American College of Sports Medicine. ACSM'S Complete Guide to Fitness & Health. 2nd ed. Champaign, IL: Human Kinetics (2017).

[31] Hakeem-Akanbi R. Investigating resistance training integration with aerobic routines to support cardiac workload tolerance, circulation efficiency, and sustained cardiovascular health. GSC Biological and Pharmaceutical Sciences. (2024) 27(2):307–20. 10.30574/gscbps.2024.27.2.0192

[32] Thijssen DHJ Bruno RM van Mil ACCM Holder SM Faita F Greyling A. Expert consensus and evidence-based recommendations for the assessment of flow-mediated dilation in humans. Eur Heart J. (2019) 40(30):2534–47. 10.1093/eurheartj/ehz350 31211361

[33] Sugawara J Tanaka H. Brachial-Ankle pulse wave velocity: myths, misconceptions, and realities. Pulse (Basel). (2015) 3(2):106–13. 10.1159/000430771 26587459 PMC4646157

[34] Stone K Veerasingam D Meyer ML Heffernan KS Higgins S Maria Bruno R. Reimagining the value of brachial-ankle pulse wave velocity as a biomarker of cardiovascular disease risk-A call to action on behalf of VascAgeNet. Hypertension. (2023) 80(10):1980–92. 10.1161/HYPERTENSIONAHA.123.21314 37470189 PMC10510846

[35] Wilhelm B Klein J Friedrich C Forst S Pfützner A Kann PH. Increased arterial augmentation and augmentation index as surrogate parameters for arteriosclerosis in subjects with diabetes mellitus and nondiabetic subjects with cardiovascular disease. J Diabetes Sci Technol. (2007) 1(2):260–3. 10.1177/193229680700100217 19888415 PMC2771462

[36] Cortez-Cooper MY Anton MM DeVan AE Neidre DB Cook JN Tanaka H. The effects of strength training on central arterial compliance in middle-aged and older adults. Eur J Cardiovasc Prev Rehabil. (2008) 15(2):149–55. 10.1097/HJR.0b013e3282f02fe2 18391640

[37] Sterne JAC Savović J Page MJ Elbers RG Blencowe NS Boutron I. Rob 2: a revised tool for assessing risk of bias in randomised trials. Br Med J. (2019) 366:l4898. Published 2019 August 28. 10.1136/bmj.l4898 31462531

[38] Nikolakopoulou A Higgins JPT Papakonstantinou T Chaimani A Del Giovane C Egger M. CINeMA: an approach for assessing confidence in the results of a network meta-analysis. PLoS Med. (2020) 17(4):e1003082. 10.1371/journal.pmed.1003082 32243458 PMC7122720

[39] Papakonstantinou T Nikolakopoulou A Higgins JPT Egger M Salanti G. CINeMA: software for semiautomated assessment of the confidence in the results of network meta-analysis. Campbell Syst Rev. (2020) 16(1):e1080. 10.1002/cl2.1080 37131978 PMC8356302

[40] White IR. Multivariate random-effects meta-regression: updates to mvmeta. Stata J. (2011) 11(2):255–70. 10.1177/1536867X1101100206

[41] Rücker G. Network meta-analysis, electrical networks and graph theory. Res Synth Methods. (2012) 3(4):312–24. 10.1002/jrsm.1058 26053424

[42] Langan D Higgins JPT Jackson D Bowden J Veroniki AA Kontopantelis E. A comparison of heterogeneity variance estimators in simulated random-effects meta-analyses. Res Synth Methods. 2019 10(1):83–98. 10.1002/jrsm.1316 30067315

[43] Chawla N Anothaisintawee T Charoenrungrueangchai K Thaipisuttikul P McKay GJ Attia J. Drug treatment for panic disorder with or without agoraphobia: systematic review and network meta-analysis of randomised controlled trials. Br Med J. (2022) 376:e066084. 10.1136/bmj-2021-066084 35045991 PMC8767458

[44] da Costa BR Juni P. Systematic reviews and meta-analyses of randomized trials: principles and pitfalls. Eur Heart J. 2014 35(47):3336–45. 10.1093/eurheartj/ehu424 25416325

[45] van Valkenhoef G Dias S Ades AE Welton NJ. Automated generation of node-splitting models for assessment of inconsistency in network meta-analysis. Res Synth Methods. (2016) 7(1):80–93. 10.1002/jrsm.1167 26461181 PMC5057346

[46] Turner RM Davey J Clarke MJ Thompson SG Higgins JP. Predicting the extent of heterogeneity in meta-analysis, using empirical data from the cochrane database of systematic reviews. Int J Epidemiol. 2012 41(3):818–27. 10.1093/ije/dys041 22461129 PMC3396310

[47] Bafeta A Trinquart L Seror R Ravaud P. Reporting of results from network meta-analyses: methodological systematic review. Br Med J. (2014) 348:g1741. 10.1136/bmj.g1741 24618053 PMC3949412

[48] Ras RT Streppel MT Draijer R Zock PL. Flow-mediated dilation and cardiovascular risk prediction: a systematic review with meta-analysis. Int J Cardiol. (2013) 168(1):344–51. 10.1016/j.ijcard.2012.09.047 23041097

[49] Munakata M. Brachial-Ankle pulse wave velocity: background, method, and clinical evidence. Pulse (Basel). (2016) 3(3-4):195–204. 10.1159/000443740 27195241 PMC4865079

[50] Ettehad D Emdin CA Kiran A Anderson SG Callender T Emberson J. Blood pressure lowering for prevention of cardiovascular disease and death: a systematic review and meta-analysis. Lancet. 2016 387(10022):957–67. 10.1016/S0140-6736(15)01225-8 26724178

[51] Ferguson D Wiley R. Scientific Evidence that Isometric Exercise Reduces Blood Pressure. Westerville, OH: MD Systems, Inc. (2002).

[52] You Q Yu L Li G He H Lv Y. Effects of different intensities and durations of aerobic exercise on vascular endothelial function in middle-aged and elderly people: a meta-analysis. Front Physiol. (2022) 12:803102. 10.3389/fphys.2021.803102 35126182 PMC8814456

[53] Pierce GL Eskurza I Walker AE Fay TN Seals DR. Sex-specific effects of habitual aerobic exercise on brachial artery flow-mediated dilation in middle-aged and older adults. Clin Sci (Lond). (2011) 120(1):13–23. 10.1042/CS20100174 20642454 PMC3809822

[54] Ho LYW Kwan RYC Yuen KM Leung WC Tam P Tsim NM. The effect of aerobic exercises on arterial stiffness in older people: a systematic review and meta-analysis. Gerontologist. (2024) 64(5):gnad123. 10.1093/geront/gnad123 37656163

[55] Li G Lv Y Su Q You Q Yu L. The effect of aerobic exercise on pulse wave velocity in middle-aged and elderly people: a systematic review and meta-analysis of randomized controlled trials. Front Cardiovasc Med. 2022 9:960096. 10.3389/fcvm.2022.960096 36061566 PMC9433655

[56] Zhou WS Zheng TT Mao SJ Xu H Wang XF Zhang SK. Comparing the effects of different exercises on blood pressure and arterial stiffness in postmenopausal women: a systematic review and meta-analysis. Exp Gerontol. (2023) 171:111990. 10.1016/j.exger.2022.111990 36397637

[57] Luo P Li J Liu K Zhang J. Effect of whole-body vibration training on arterial stiffness in adults: a systematic review and meta-analysis of randomized controlled trials. Blood Press. (2025) 34(1):2571416. 10.1080/08037051.2025.2571416 41054800

[58] Lee MC Lai CL Chen HY Tseng SY Liao WC Liu BT. Effect of whole-body vibration for 3 months on arterial stiffness in the middle-aged and elderly. Clin Interv Aging. (2014) 9:821–8. 10.2147/CIA.S60029 24872684 PMC4026558

[59] Adams JA Martínez A. Editorial: non-pharmacologic sustained endothelial shear stress: an evolving clinical paradigm. Front Physiol. (2021) 12:790022. 10.3389/fphys.2021.790022 34867490 PMC8635141

[60] Ahn Y Aung N Ahn HS. A comprehensive review of clinical studies applying flow-mediated dilation. Diagnostics (Basel). 2024 14(22):2499. 10.3390/diagnostics14222499 39594169 PMC11592698

[61] Adams JA Uryash A Lopez JR. Non-Invasive pulsatile shear stress modifies endothelial activation; A Narrative Review. Biomedicines. (2022) 10(12):3050. 10.3390/biomedicines10123050 36551807 PMC9775985

[62] Francois ME Durrer C Pistawka KJ Halperin FA Little JP. Resistance-based interval exercise acutely improves endothelial function in type 2 diabetes. Am J Physiol Heart Circ Physiol. (2016) 311(5):H1258–67. 10.1152/ajpheart.00398.2016 27638878 PMC5130498

[63] Kim HB Seo MW Jung HC. Effects of aerobic vs. Resistance exercise on vascular function and vascular endothelial growth factor in older women. Healthcare (Basel). (2023) 11(18):2479. 10.3390/healthcare11182479 37761675 PMC10530817

[64] Zang Y Ding X Zhao MX Zhang X Zhang Li Wu S. Arterial stiffness acute changes following aerobic exercise in males with and without hypertension. J Clin Hypertens (Greenwich). (2022) 24(4):430–7. 10.1111/jch.14461 35285576 PMC8989744

[65] Tanaka H Dinenno FA Monahan KD Clevenger CM DeSouza CA Seals DR. Aging, habitual exercise, and dynamic arterial compliance. Circulation. (2000) 102(11):1270–5. 10.1161/01.cir.102.11.1270 10982542

[66] Pierce GL. Aortic stiffness in aging and hypertension: prevention and treatment with habitual aerobic exercise. Curr Hypertens Rep. (2017) 19(11):90. 10.1007/s11906-017-0788-0 29046980 PMC10949831

[67] Nowak KL Rossman MJ Chonchol M Seals DR. Strategies for achieving healthy vascular aging. Hypertension. (2018) 71(3):389–402. 10.1161/HYPERTENSIONAHA.117.10439 29311256 PMC5812814

[68] Ko J Deprez D Shaw K Alcorn J Hadjistavropoulos T Tomczak C. Stretching is superior to brisk walking for reducing blood pressure in people with high-normal blood pressure or stage I hypertension. J Phys Act Health. (2021) 18(1):21–8. 10.1123/jpah.2020-0365 33338988

[69] Kim HL. Arterial stiffness and hypertension. Clin Hypertens. (2023) 29(1):31. 10.1186/s40885-023-00258-1 38037153 PMC10691097

[70] Li Z Lv M Li Z Gao W Li M. Physiological characteristics of blood pressure responses after combined exercise in elderly hypertensive patients: a systematic review and meta-analysis. Front Cardiovasc Med. (2024) 11:1404127. 10.3389/fcvm.2024.1404127 39526180 PMC11543474

[71] Shimojo GL da Silva Dias D Malfitano C Sanches IC Llesuy S Ulloa L. Combined aerobic and resistance exercise training improve hypertension associated with menopause. Front Physiol. (2018) 9:1471. 10.3389/fphys.2018.01471 30420811 PMC6215975

