# Supplementary material for: Comparative effectiveness of various exercise modalities on arterial stiffness and endothelial function in older adults: a systematic review and network meta-analysis of randomised controlled trials
Source: Front Cardiovasc Med. 2026 Jul 13;13:1815768. doi: 10.3389/fcvm.2026.1815768 (PMC13402533; doi:10.3389/fcvm.2026.1815768)

Supplementary Appendix

Comparative effectiveness of different exercise modalities on arterial stiffness and endothelial function in older adults: a systematic review and network meta-analysis of randomized controlled trials

Table of contents

Appendix 1: Search strategy 1

Appendix 2: Characteristics of included studies 6

Appendix 3: List of data extracted from the included randomized clinical trials 15

Appendix 4: Risk of bias of randomized clinical trials 16

Appendix 5: Evaluation of inconsistency and heterogeneity 19

Appendix 6: Network maps and forest plots of secondary outcomes 26

Appendix 7: SUCRA and cumulative probability plots 33

Appendix 8: League table of relative effect estimates for different exercise modalities on arterial stiffness and endothelial function: A network meta-analysis of 47 trials. 45

Appendix 9: CINeMA Assessment 57

Appendix 10: Funnel plots 76

Appendix 11: Subgroup analysis 88

Appendix 1: Search strategy

Table S1. Search strategy of PubMed

#	Searches
1	vascular stiffness[MeSH Terms]
2	vascular stiffness[MeSH Terms](((((((Stiffness, Vascular[Title/Abstract]) OR (Vascular Stiffnesses[Title/Abstract])) OR (Arterial Stiffness[Title/Abstract])) OR (Arterial Stiffnesses[Title/Abstract])) OR (Stiffness, Arterial[Title/Abstract])) OR (Aortic Stiffness[Title/Abstract])) OR (Aortic Stiffnesses[Title/Abstract])) OR (Stiffness, Aortic[Title/Abstract])
3	Pulse Wave Analysis[MeSH Terms]
4	((((((((((((((((Analyses, Pulse Wave[Title/Abstract]) OR (Analysis, Pulse Wave[Title/Abstract])) OR (Pulse Wave Analyses[Title/Abstract])) OR (Wave Analyses, Pulse[Title/Abstract])) OR (Wave Analysis, Pulse[Title/Abstract])) OR (Pulse Wave Velocity[Title/Abstract])) OR (Pulse Wave Velocities[Title/Abstract])) OR (Velocities, Pulse Wave[Title/Abstract])) OR (Velocity, Pulse Wave[Title/Abstract])) OR (Wave Velocities, Pulse[Title/Abstract])) OR (Wave Velocity, Pulse[Title/Abstract])) OR (Pulse Transit Time[Title/Abstract])) OR (Pulse Transit Times[Title/Abstract])) OR (Time, Pulse Transit[Title/Abstract])) OR (Times, Pulse Transit[Title/Abstract])) OR (Transit Time, Pulse[Title/Abstract])) OR (Transit Times, Pulse[Title/Abstract])) OR (Pulse Wave Transit Time[Title/Abstract])
5	#1 OR #2
6	#3 OR #4
7	#5 AND #6
8	Endothelium, Vascular[MeSH Terms]
9	((((((Vascular Endothelium[Title/Abstract]) OR (Endotheliums, Vascular[Title/Abstract])) OR (Vascular Endotheliums[Title/Abstract])) OR (Capillary Endothelium[Title/Abstract])) OR (Capillary Endotheliums[Title/Abstract])) OR (Endothelium, Capillary[Title/Abstract])) OR (Endotheliums, Capillary[Title/Abstract])
10	(((((((((((((((((((((Exercise[MeSH Terms]) OR (Exercise, Physical[Title/Abstract])) OR (Physical Exercise[Title/Abstract])) OR (Exercise, Aerobic[Title/Abstract])) OR (Aerobic Exercises[Title/Abstract])) OR (Exercises, Aerobic[Title/Abstract])) OR (Isometric Exercises[Title/Abstract])) OR (Acute Exercise[Title/Abstract])) OR (Exercise, Acute[Title/Abstract])) OR (Exercise Training[Title/Abstract])) OR (Training, Exercise[Title/Abstract])) OR (Physical Activity[Title/Abstract])) OR (Strength Training[Title/Abstract])) OR (Training, Strength[Title/Abstract])) OR (Strengthening Programs, Weight-Bearing[Title/Abstract])) OR (Aerobic Exercise[Title/Abstract])) OR (Exercise, Isometric[Title/Abstract])) OR (Exercise, Muscle Stretching[Title/Abstract])) OR (Stretching, Active[Title/Abstract])) OR (Stretching, Isometric[Title/Abstract])) OR (Tai-ji[Title/Abstract])) OR (Tai Chi[Title/Abstract])) OR (Chi, Tai[Title/Abstract])) OR (Walking[Title/Abstract])
11	(((((((Aged[MeSH Terms]) OR (Middle Aged[MeSH Terms])) OR (Aged, 80 and over[MeSH Terms])) OR (Oldest Old[Title/Abstract])) OR (senior[Title/Abstract])) OR (Elderly[Title/Abstract])) OR (frail elderly[Title/Abstract])) OR (geriatric[Title/Abstract])
12	#8 OR #9
13	#10 AND #11AND #12
14	((((((((((((randomized controlled trial[Publication Type]) OR (controlled clinical trial[Publication Type])) OR (clinical trials as topic[MeSH Terms])) OR (random allocation[MeSH Terms])) OR (randomized controlled trials as topic[MeSH Terms])) OR (clinical trial[Publication Type])) OR (clinical trial[Text Word])) OR (singl*[Text Word])) OR (doubl*[Text Word])) OR (trebl*[Text Word])) OR (tripl*[Text Word])) OR (randomly[Text Word])) OR (trial[Text Word])
15	#7 AND #13 AND #14

Table S2. Search strategy of Web of Science

#	Searches
1	TS=(vascular stiffness)
2	(((((((TS=(Stiffness, Vascular) OR TS=(Vascular Stiffnesses)) OR TS=(Arterial Stiffness)) OR TS=(Arterial Stiffnesses)) OR TS=(Stiffness, Arterial)) OR TS=(Aortic Stiffness)) OR TS=(Aortic Stiffnesses)) OR TS=(Stiffness, Aortic)
3	TS=(Pulse Wave Analysis)
4	((((((((((((((((TS=(Analyses, Pulse Wave) OR TS=(Analysis, Pulse Wave)) OR TS=(Pulse Wave Analyses)) OR TS=(Wave Analyses, Pulse)) OR TS=(Wave Analysis, Pulse)) OR TS=(Pulse Wave Velocity)) OR TS=(Pulse Wave Velocities)) OR TS=(Velocities, Pulse Wave)) OR TS=(Velocity, Pulse Wave)) OR TS=(Wave Velocities, Pulse)) OR TS=(Wave Velocity, Pulse)) OR TS=(Pulse Transit Time)) OR TS=(Pulse Transit Times)) OR TS=(Time, Pulse Transit)) OR TS=(Times, Pulse Transit)) OR TS=(Transit Time, Pulse)) OR TS=(Transit Times, Pulse)) OR TS=(Pulse Wave Transit Time)
5	#1 OR #2
6	#3 OR #4
7	#5 AND #6
8	TS=(Endothelium, Vascular)
9	(((((TS=(Vascular Endothelium) OR TS=(Endotheliums, Vascular)) OR TS=(Vascular Endotheliums)) OR TS=(Capillary Endothelium)) OR TS=(Capillary Endotheliums)) OR TS=(Endothelium, Capillary)) OR TS=(Endotheliums, Capillary)
10	((((((((((((((((((((((TS=(Exercise) OR TS=(Exercise, Physical)) OR TS=(Physical Exercise)) OR TS=(Exercise, Aerobic)) OR TS=(Aerobic Exercises)) OR TS=(Exercises, Aerobic)) OR TS=(Isometric Exercises)) OR TS=(Acute Exercise)) OR TS=(Exercise, Acute)) OR TS=(Exercise Training)) OR TS=(Training, Exercise)) OR TS=(Physical Activity)) OR TS=(Strength Training)) OR TS=(Training, Strength)) OR TS=(Strengthening Programs, Weight-Bearing)) OR TS=(Aerobic Exercise)) OR TS=(Exercise, Isometric)) OR TS=(Exercise, Muscle Stretching)) OR TS=(Stretching, Active)) OR TS=(Stretching, Isometric)) OR TS=(Tai-ji)) OR TS=(Tai Chi)) OR TS=(Chi, Tai)) OR TS=(Walking)
11	((((((TS=(Aged) OR TS=(Middle Aged)) OR TS=(Aged, 80 and over)) OR TS=(Oldest Old)) OR TS=(senior)) OR TS=(Elderly)) OR TS=(frail elderly)) OR TS=(geriatric)
12	#8 OR #9
13	#10 AND #11 AND #12
14	((((((((((((TS=(randomized controlled trial) OR PT=(Controlled Clinical Trial)) OR TS=(clinical trials as topic)) OR TS=(random allocation)) OR TS=(randomized controlled trials as topic)) OR PT=(Clinical Trial)) OR TS=(clinical trial)) OR TS=(singl*)) OR TS=(doubl*)) OR TS=(trebl*)) OR TS=(tripl*)) OR TS=(randomly)) OR TS=(trial)
15	#7 AND #13 AND #14

Table S3. Search strategy of Embase

#	Searches
1	(((((((Stiffness, Vascular).ab,ti OR (Vascular Stiffnesses).ab,ti) OR (Arterial Stiffness).ab,ti) OR (Arterial Stiffnesses).ab,ti) OR (Stiffness, Arterial).ab,ti) OR (Aortic Stiffness).ab,ti) OR (Aortic Stiffnesses).ab,ti) OR (Stiffness, Aortic).ab,ti
2	((((((((((((((((Analyses, Pulse Wave).ab,ti OR (Analysis, Pulse Wave).ab,ti) OR (Pulse Wave Analyses).ab,ti) OR (Wave Analyses, Pulse).ab,ti) OR (Wave Analysis, Pulse).ab,ti) OR (Pulse Wave Velocity).ab,ti) OR (Pulse Wave Velocities).ab,ti) OR (Velocities, Pulse Wave).ab,ti) OR (Velocity, Pulse Wave).ab,ti) OR (Wave Velocities, Pulse).ab,ti) OR (Wave Velocity, Pulse).ab,ti) OR (Pulse Transit Time).ab,ti) OR (Pulse Transit Times).ab,ti) OR (Time, Pulse Transit).ab,ti) OR (Times, Pulse Transit).ab,ti) OR (Transit Time, Pulse).ab,ti) OR (Transit Times, Pulse).ab,ti) OR (Pulse Wave Transit Time).ab,ti
3	#1 AND #2
4	(((((((Vascular Endothelium).ab,ti OR (Endotheliums, Vascular).ab,ti) OR (Vascular Endotheliums).ab,ti) OR (Capillary Endothelium).ab,ti) OR (Capillary Endotheliums).ab,ti) OR (Endothelium, Capillary).ab,ti) OR (Endotheliums, Capillary).ab,ti
5	((((((((((((((((((((((Exercise).mesh OR (Exercise, Physical).ab,ti) OR (Physical Exercise).ab,ti) OR (Exercise, Aerobic).ab,ti) OR (Aerobic Exercises).ab,ti) OR (Exercises, Aerobic).ab,ti) OR (Isometric Exercises).ab,ti) OR (Acute Exercise).ab,ti) OR (Exercise, Acute).ab,ti) OR (Exercise Training).ab,ti) OR (Training, Exercise).ab,ti) OR (Physical Activity).ab,ti) OR (Strength Training).ab,ti) OR (Training, Strength).ab,ti) OR (Strengthening Programs, Weight-Bearing).ab,ti) OR (Aerobic Exercise).ab,ti) OR (Exercise, Isometric).ab,ti) OR (Exercise, Muscle Stretching).ab,ti) OR (Stretching, Active).ab,ti) OR (Stretching, Isometric).ab,ti) OR (Tai-ji).ab,ti) OR (Tai Chi).ab,ti) OR (Chi, Tai).ab,ti) OR (Walking).ab,ti
6	(((((('Aged'.mesh OR 'Middle Aged'.mesh) OR 'Aged, 80 and over'.mesh) OR (Oldest Old).ab,ti) OR (senior).ab,ti) OR (Elderly).ab,ti) OR (frail elderly).ab,ti) OR (geriatric).ab,ti
7	#4 AND #5 AND #6
8	((((((((((('randomized controlled trial'.pt OR 'controlled clinical trial'.pt) OR 'clinical trials as topic'.mesh) OR 'random allocation'.mesh) OR 'randomized controlled trials as topic'.mesh) OR 'clinical trial'.pt) OR (clinical trial).ab,ti) OR (singl*).ab,ti) OR (doubl*).ab,ti) OR (trebl*).ab,ti) OR (tripl*).ab,ti) OR (randomly).ab,ti) OR (trial).ab,ti
9	#3 AND #7 AND #8

Table S4. Search strategy of Cochrane Central Register of Controlled Trials

#	Searches
1	MeSH descriptor:[Vascular Stiffness] explode all trees
2	(Stiffness, Vascular or Vascular Stiffnesses or Arterial Stiffness or Arterial Stiffnesses or Stiffness, Arterial or Aortic Stiffness or Aortic Stiffnesses or Stiffness, Aortic):ti,ab,kw
3	MeSH descriptor:[Pulse Wave Analysis]explode all trees
4	(Analyses, Pulse Wave or Analysis, Pulse Wave or Pulse Wave Analyses or Wave Analyses, Pulse or Wave Analysis, Pulse or Pulse Wave Velocity or Pulse Wave Velocities or Velocities, Pulse Wave or Velocity, Pulse Wave or Wave Velocities, Pulse or Wave Velocity, Pulse or Pulse Transit Time or Pulse Transit Times or Time, Pulse Transit or Times, Pulse Transit or Transit Time, Pulse or Transit Times, Pulse or Pulse Wave Transit Time):ti,ab,kw
5	#1 OR #2
6	#3 OR #4
7	#5 AND #6
8	MeSH descriptor:[Vascular Endothelium] explode all trees
9	(Vascular Endothelium or Endotheliums, Vascular or Vascular Endotheliums or Capillary Endothelium or Capillary Endotheliums or Endothelium, Capillary or Endotheliums, Capillary):ti,ab,kw
10	MeSH descriptor: [Exercise] explode all trees
11	(Exercises or Exercise, Physical or Exercises, Physical or Physical Exercise or Physical Exercises or Exercise, Aerobic or Aerobic Exercise or Aerobic Exercises or Exercises, Aerobic or Exercise, Isometric or Exercises, Isometric or Isometric Exercises or Isometric Exercise or Acute Exercise or Acute Exercises or Exercise, Acute or Exercises, Acute or Exercise Training or Exercise Trainings or Training, Exercise or Trainings, Exercise or Physical Activity or Activities, Physical or Activity, Physical or Physical Activities):ti,ab,kw
12	#8 OR #9
13	#10 OR #11
14	#12 AND #13
15	MeSH descriptor: [Randomized Controlled Trial] explode all trees
16	(controlled clinical trial or clinical trial or clinical trials as topic or randomized controlled trials as topic or random allocation or clinical trial or randomly or trial or singl*or doubl* or trebl*or tripl*):ti,ab,kw
17	#15 OR #16
18	#7 AND #14AND #17

Table S5. Search strategy of CNKI , VIP Database, Wanfang Data, SinoMed

#	Searches
1	(((((((TS=(Stiffness, Vascular)) OR TS=(Vascular Stiffnesses)) OR TS=(Arterial Stiffness)) OR TS=(Arterial Stiffnesses)) OR TS=(Stiffness, Arterial)) OR TS=(Aortic Stiffness)) OR TS=(Aortic Stiffnesses)) OR TS=(Stiffness, Aortic)
2	((((((((((((((((TS=(Analyses, Pulse Wave)) OR TS=(Analysis, Pulse Wave)) OR TS=(Pulse Wave Analyses)) OR TS=(Wave Analyses, Pulse)) OR TS=(Wave Analysis, Pulse)) OR TS=(Pulse Wave Velocity)) OR TS=(Pulse Wave Velocities)) OR TS=(Velocities, Pulse Wave)) OR TS=(Velocity, Pulse Wave)) OR TS=(Wave Velocities, Pulse)) OR TS=(Wave Velocity, Pulse)) OR TS=(Pulse Transit Time)) OR TS=(Pulse Transit Times)) OR TS=(Time, Pulse Transit)) OR TS=(Times, Pulse Transit)) OR TS=(Transit Time, Pulse)) OR TS=(Transit Times, Pulse)) OR TS=(Pulse Wave Transit Time)
3	(((((((TS=(Vascular Endothelium)) OR TS=(Endotheliums, Vascular)) OR TS=(Vascular Endotheliums)) OR TS=(Capillary Endothelium)) OR TS=(Capillary Endotheliums)) OR TS=(Endothelium, Capillary)) OR TS=(Endotheliums, Capillary)
4	#1 AND #2AND #3
5	((((((TS=(Exercise therapy)) OR TS=(Rehabilitation exercise)) OR TS=(Exercise training)) OR TS=(Physical exercise)) OR TS=(Aerobic exercise)) OR TS=(Resistance exercise)) OR TS=(Exercise intervention)
6	((((((TS=(Aged)) OR TS=(Middle Aged)) OR TS=(Aged, 80 and over)) OR TS=(Oldest Old)) OR TS=(senior)) OR TS=(Elderly)) OR TS=(frail elderly)) OR TS=(geriatric)
7	((TS=(Randomized controlled trials)) OR TS=(RCT OR Random assignment)) OR TS=(Randomized trials))
8	#5 AND #6 AND #7
9	#4 AND #8

Appendix 2: Characteristics of included studies

Table S2.1: Baseline of characteristics of included studies

Study	Trial registration	Country and region	Design	Number of participants	Randomised treatments	Age (mean ± SD), years	BMI (mean ± SD), kg/m2	Exercise frequency	Outcome measures	Gender
**Westhoff 2007[1]**	NCT00315224	Germany	RCT	54	AT ：27<br>CON：27	67.2 ± 4.8<br>68.9 ± 5.2	27.7 ± 4.4<br>29.8 ± 4.3	Experimental group: 3 times weekly<br>Control group: No structured exercise	①⑦⑧	Male:26<br>Female:28
**Lee 2019[2]**	NCT03544307	USA	RCT	20	CT：10<br>CON：10	70 ± 4<br>70 ± 4	25.0 ± 1.6<br>25.4 ± 3.4	Taekwondo training group: 3 times/week<br>Control group: No structured exercise (maintained normal sedentary lifestyle)	⑦⑧	Male:0<br>Female:20
**Gholami 2021[3]**	IRCT20170120032066N4	Iran	RCT	34	RT:17<br>CON:17	63 ± 3<br>64 ± 3	none<br>none	Experimental group: 3 times/week<br>Control group: No structured resistance training	⑥⑩	Male:34<br>Female:0
**Suboc 2014[4]**	NCT01212978	USA	RCT	107	WT:36<br>WT:30<br>CON:41	64 ± 7<br>63 ± 8<br>62 ± 7	28.8 ± 4.9<br>29.7 ± 5.5<br>none	Pedometer-only group: 12-week intervention, no fixed weekly frequency, targeting ≥ 10000 steps/day with 10% weekly increment<br>Pedometer + interactive website group: 12-week intervention, no fixed weekly frequency, targeting ≥10000 steps/day with website-based motivational support<br>Control group: No structured exercise	①④⑥⑦⑧	Male:71<br>Female:36
**Fujie 2014[5]**	none	Japan	RCT	34	AT：18<br>CON：16	66.4 ± 2.1<br>67.8 ± 1.5	24.7 ± 1.0<br>21.8 ± 0.9	Experimental group: 3 days/week (8-week aerobic exercise training, 45 min/session at 60–70% VO_2peak, cycling on leg ergometer)<br>Control group: No structured exercise (maintained habitual sedentary lifestyle)	⑦⑧⑨	Male:14<br>Female:20
**Shimomura 2021[6]**	UMIN000035520	Japan	RCT	31	AT：16<br>CON:15	64.8 ± 2.0<br>68.1 ± 1.6	24.6 ± 1.1<br>22.0 ± 1.0	Experimental group: 3 days/week (8-week aerobic exercise training, 45 min/session at 60–70% VO_2peak, cycling on leg ergometer initial 2 weeks at 50% VO_2peak for 40 min, with 5-min warm-up/cool-down at 40% VO_2peak)<br>Control group: No structured exercise	⑦⑧⑨	Male:13<br>Female:18
**Ploydang 2023[7]**	none	Thailand	RCT	36	WT:18<br>CON:18	68.9 ± 3.7<br>69.2 ± 5.3	24.2 ± 3.6<br>24.8 ± 2.7	Experimental group: 3 times/week (12-week aquatic Nordic walking, 40 min exercise bout/session in 34°C–36°C water 40%–50% HRR for first 6 weeks, 50%–60% HRR for last 6 weeks, total session including warm-up/cool-down: 60 min)<br>Control group: No structured exercise	①③⑦⑧	Male:12<br>Female:24
**Otsuki 2019[8]**	none	Japan	RCT	49	AT：23<br>CON:26	67 ± 8<br>65 ± 7	22 ± 2, 6<br>22 ± 2	Experimental group: 4.4 ± 1.3 days/week (6-week aerobic exercise 1 supervised walking session/week [35–50 min] + 2–4 self-directed walking sessions/week intensity from 60–65% to 75% age-predicted maximal HR, 59 ± 20 min/session)<br>Control group: No structured exercise	③⑦⑧	Male:20<br>Female:29
**Novakovic 2019[9]**	NCT02642276	Slovenia	RCT	36	AT：12<br>WT:12<br>CON:12	65.6 ± 11.0<br>65.1 ± 7.6<br>62.0 ± 8.3	27.3 ± 3.3<br>30.4 ± 6.2<br>28.5 ± 2.3	Moderate-pain/pain-free training groups: 2–3 times/week (36 sessions total, ~60 min/session, intensity 70% predicted maximal heart rate)<br>Control group: No supervised exercise	①⑦⑧⑨⑩	Male:27<br>Female:9
**Yasuda 2013[10]**	none	Japan	RCT	19	RT:9<br>CON:10	71 ± 7<br>67.7 ± 6.0	20.8 ± 2.6<br>21.3 ± 2.9	Experimental group: 2 days/week (12-week training, 75 repetitions per exercise [30/20/15/10 reps with 30-s rest between sets], 20% 1-RM for knee extension, 30% 1-RM for leg press, elastic cuffs 120–270 mmHg)<br>Control group: No structured resistance training	①⑦⑧⑩	Male:4<br>Female:15
**Miura 2008[11]**	none	Japan	RCT	77	CT:29<br>CT:25<br>CON:23	69.0 ± 6.5<br>69.5 ± 7.0<br>68.9 ± 7.5	22.8 ± 2.4<br>23.5 ± 2.7<br>23.7 ± 3.0	1DW/2DW groups: 1/2 times/week (12-week group training, 90 min/session 40-min circuit training with 6–8 resistance exercises [15–20 reps/group, 3–5 groups], 20-min chair-based leg exercises)<br>Control group: No regular exercise	③⑦⑧	Male:0<br>Female:77
**Fujie 2025[12]**	UMIN000038253	Japan	RCT	59	RT:20<br>RT:18<br>CON:21	66.9 ± 5.0<br>67.2 ± 5.5<br>67.6 ± 6.4	none	RT/RT+HP groups: 3 days/week (12-week resistance training, 3 sets × 10 reps of seated leg extension/curls at 70% 1-RM, 2-min rest between sets, 3-min treadmill warm-up + 5-min lower limb stretch cool-down)<br>CON group：additionally consumed carbohydrate-rich food 3 times/week	②⑦⑧⑨	Male:0<br>Female:59

**Jaime 2019[13]**	none	USA	RCT	33	WBV:13<br>RT:12<br>CON:8	64 ± 1<br>64 ± 1<br>67 ± 1	23.2 ± 0.8<br>24.0 ± 0.6<br>22.5 ± 0.9	WBVT/LIRET groups: 12-week intervention (20–35 min/session, progressive intensity<br>WBVT: 24–40 Hz vibration, 2–3 sets/exercise<br>LIRET: 40% 1-RM, 15 reps/set, slow contractions)<br>Control group: No structured exercise	①②③④⑤⑦⑧⑪	Male:0<br>Female:33
**Bellia 2017[14]**	none	Italy	RCT	22	AT:11<br>WT:11	58.8 ± 7.9<br>56.3 ± 6.4	27.7 ± 2.8<br>29.9 ± 3.4	AIT group: Progressive frequency (Weeks 1-2: 2 times/week Weeks 3-4: 3 times/week [2 reps/session] Weeks 5-6: 3 times/week [3 reps/session] Weeks 7-12: 3 times/week [4 reps/session] 4-min work bouts at 75-80% HRmax, 3-min recovery at 45-50% HRmax)<br>SOC group: 70,000 steps/week or 10,000 steps/day (unsupervised brisk walking)	④⑤⑦⑧	Male:16<br>Female:6
**Kim 2023[15]**	none	Korea	RCT	43	AT：14<br>RT：15<br>CON：14	68.4 ± 3.51<br>68.3 ± 3.45<br>67.5 ± 5.17	24.3 ± 1.9<br>24.3 ± 1.5<br>24.9 ± 2.8	AE/RE groups: 3 days/week (16-week intervention, 60 min/session AE: 10-min warm-up + 40-min aerobic exercise + 10-min cool-down, 40–60% heart rate reserve<br>RE: 10-min warm-up + 40-min resistance exercise + 10-min cool-down, RPE 12–13)<br>CG group: No structured exercise (maintained habitual lifestyle)	③	Male:0<br>Female:43
**Ohta 2012[16]**	none	Japan	RCT	26	AT：13<br>CON:13	72.2 ± 4.2<br>71.5 ± 7.4	23.0 ± 2.6<br>: 21.8 ± 2.6	Bench step exercise group: 3 times/day, 10–20 min/session (140 min/week total, 12-week intervention, intensity at lactate threshold) Control group: No structured exercise	③⑦⑧	Male:0<br>Female:26
**Bouaziz 2019[17]**	NCT02263573	France	RCT	56	AT：27<br>CON:29	72.9 ± 2.5<br>74.3 ± 3.4	28.7 ± 5.3<br>28.8 ± 5.0	IATP-R group: 2 times/week (9.5-week intervention, 19 sessions total 30 min/session: 3-min warm-up + 6×(4-min at VT1 intensity + 1-min at 40% VT1 intensity) + 3-min cool-down)<br>Control group: No structured exercise	①②⑦⑧	Male:15<br>Female:41
**Lai 2014[18]**	none	China	RCT	38	WBV：19<br>CON：19	60.1 ± 5.9<br>63.6 ± 9.8	23.5 ± 1.9<br>22.9 ± 1.9	WBV group: 3 times/week (3-month intervention, 5 min/session 30 Hz frequency, 3.2 g acceleration, natural full standing posture)<br>CON group: No structured exercise	③⑦⑧	Male:17<br>Female:21
**Kitzman 2013[19]**	NCT01113840	USA	RCT	54	AT：24<br>CON:30	70 ± 7<br>70 ± 7	32.2 ± 6.7<br>32.0 ± 6.6	ET group: 3 times/week (16-week intervention, 60 min/session 10-min warm-up + 40-min training [track walking, cycle ergometry, arm ergometry] + 10-min cool-down intensity from 40–50% HRR to 70% HRR)<br>CT group: No structured exercise (telephone follow-ups every 2 weeks)	①⑦⑧⑨	Male:13<br>Female:41
**Patil 2015[20]**	CTRI/2011/10/002077	India	RCT	60	SE：30<br>WT：30	68.50 ± 4.85<br>69.30 ± 5.93	24.64 ± 3.65<br>25.17 ± 3.90	Yoga/BW groups: 6 days/week (12-week intervention, 60 min/session in the morning Yoga: 15–20 min loosening/asanas + 40–45 min pranayama/meditation BW: 20 min stretching + 35 min brisk-walking + 5 min rest)<br>No control group with no exercise	②③④⑤⑦⑧	Male:60<br>Female:0
**Oliveira 2015[21]**	NCT01432639	Portugal	RCT	78	AT：37<br>CON:41	55.0 ± 10.7<br>58.5 ± 10.7	26.1<br>26.9	EG: 3 times/week (8-week intervention, 50 min/session 10-min warm-up + 30-min aerobic exercise [cycloergometer/treadmill] at 70–85% HRmax + 10-min cool-down)<br>CG: No structured exercise	②④⑤⑦⑧	Male:65<br>Female:13
**Kobayashi 2022[22]**	none	Japan	RCT	45	AT：15<br>AT：15<br>CON:15	62.5 ± 2.8<br>62.9 ± 1.9<br>60.9 ± 3.9	22.4 ± 1.6<br>22.8 ± 1.1<br>22.7 ± 1.4	LF group: 2 times/week (8-week intervention, 30 min/session jogging/running at 65% heart rate reserve) HF group: 4 times/week (8-week intervention, 30 min/session jogging/running at 65% heart rate reserve)<br>CON group: No structured exercise	②③⑦⑧	Male:0<br>Female:45
**Otsuki 2020[23]**	none	Japan	RCT	27	CT：12<br>CON：15	68 ± 2<br>64 ± 2	21 ± 1<br>22 ± 1	CET group: 3 times/week (6-week intervention 1 supervised session + 2 home-based sessions/week 30-min walking [60–75% age-predicted maximal HR] + lower-extremity bodyweight resistance training [2–3 sets × 8–10 reps])<br>CON group: No structured exercise)	③⑦⑧	Male:11<br>Female:16
**Figueroa 2011[24]**	none	USA;Korea	RCT	24	CT：12<br>CON：12	54 ± 2<br>54 ± 1	24.2 ± 0.7<br>23.1 ± 0.7	EX group: 3 times/week (12-week intervention, 40 min/session 20-min circuit resistance training [9 exercises, 1 set × 12 reps, <15 s interset rest, 60% predicted maximal HR] + 20-min treadmill walking [60% predicted maximal HR])<br>Control group: No structured exercise	③⑦⑧	Male:0<br>Female:24
**Son 2017[25]**	none	Korea; USA	RCT	20	CT：10<br>CON：10	76 ± 5<br>76 ± 5	22.77 ± 0.7<br>24.71 ± 0.4	EX group: 3 times/week (12-week intervention, 70 min/session 5-min warm-up + 20-min resistance band training [upper/lower body exercises] + 30-min walking + 5-min cool-down intensity: 40–50% HRR [Weeks 1–4], 50–60% HRR [Weeks 5–8], 60–70% HRR [Weeks 9–12])<br>CON group: No structured exercise	③⑦⑧	Male:0<br>Female:20
**Shin 2015[26]**	none	Korea	RCT	43	MBE：29<br>CON：14	64.0 ± 5.4<br>62.7 ± 5.9	22.5 ± 2.7<br>23.4 ± 3.2	Tai Chi group: 1 time/week (3-month intervention, 60 min/session group-based Tai Chi practice with slow, gentle movements and deep breathing)<br>Control group: No structured exercise	①③⑦⑧	Male:0<br>Female:43

**Kim 2017[27]**	none	USA;Korea	RCT	49	CT：17<br>CT：18<br>CON：14	64 ± 1<br>64 ± 1<br>64 ± 1	none	HIIT group: 4 times/week (8-week intervention, 40 min/session 10-min warm-up + 4×4 min at 90% HRpeak + 3×3 min active recovery at 70% HRpeak + 5-min cool-down)<br>MICT group: 4 times/week (8-week intervention, 47 min/session 10-min warm-up + 32-min continuous exercise at 70% HRpeak + 5-min cool-down)<br>CONT group: No structured exercise	②⑦⑧	Male:16<br>Female:33
**Hasegawa 2018[28]**	none	Japan	RCT	52	AT：26<br>CON:26	68.0 ± 7.1<br>65.8 ± 8.6	23.5 ± 3.5<br>24.7 ± 4.7	Training group: 3 times/week (8-week intervention, 55 min/session 5-min warm-up at 40% VO_2peak + 45-min cycling at 60–70% VO_2peak + 5-min cool-down at 40% VO_2peak)<br>Control group: No structured exercise	②⑦⑧	Male:26<br>Female:26
**Madden 2013[29]**	none	Canada	RCT	52	AT：25<br>RT：27	68.5 ± 0.9<br>70.0 ± 0.8	30.9 ± 1.0<br>28.6 ± 0.8	AT group: 3 times/week (6-month intervention, 60 min/session 10-min warm-up + 40-min aerobic exercise [treadmill/cycle ergometer] at 60–75% heart rate reserve + 10-min cool-down<br>NA group: 3 times/week (6-month intervention, low-intensity non-aerobic training, no aerobic component)	⑦⑧	Male:30<br>Female:22
**Cui 2019[30]**	none	China	RCT	60	MBE：30<br>CON:30	64.72 ± 5.38<br>65.91 ± 4.57	19.9 ± 2.7<br>19.5 ± 3.2	Tai Chi group: 3 times/week (24-week intervention, 60 min/session 5–10 min warm-up + 40–50 min 24-style Tai Chi practice + 5–10 min cool-down intensity controlled at target heart rate = (max HR - resting HR)×50% + resting HR)<br>Control group: No structured exercise	③⑩	Male:30<br>Female:30
**Pierce 2011[31]**	none	USA	RCT	26	AT：13<br>CON:13	65.91±4.57<br>63±3.1	26.8 ± 0.5<br>25.9 ± 0.5	Intervention study: Exercise group: 6 days/week (8-week intervention, 48.6 ± 1.1 min/session, 71.1 ± 0.8% maximal HR) Control group: No structured aerobic exercise	①⑦⑧	Male:11<br>Female:15
**Park 2020[32]**	none	Korea	RCT	22	CT：11<br>CON：11	69.1 ± 0.9<br>68.5 ± 0.9	26.2 ± 0.5<br>26.0 ± 0.4	EXP group: 3 times/week (12-week intervention, 90–120 min/session 30–40 min elastic-band resistance training [6 exercises, 3 sets × 10–15 reps, OMNI-RES 6–7, 90 s rest/set] + 10-min rest + 60-min aerobic exercise [30-min treadmill + 30-min bicycle, 60–70% maximal HR])<br>CON group: No structured exercise	③⑦⑧	Male:22<br>Female:0
**Rech 2019[33]**	NCT02548000	Brazil	RCT	39	RT：18<br>CON：21	70.5 ± 7.4<br>68.0 ± 6.5	28.46 ± 3.2<br>28.3 ± 3.1	RT group: 3 times/week (12-week intervention) functional exercises (2-3 sets, 10-15 reps) + traditional resistance exercises (2-3 sets, 10-12 reps) 1-min rest between sets<br>AC group: 1 time/week (12-week intervention) 20-30 s static stretches for large muscle groups, total ~45 min/session	①	Male:20<br>Female:19
**Haynes 2021[34]**	ACTRN12614000017628	Australia; Canada	RCT	51	WT：17<br>WT：18<br>CON：16	61.9 ± 5.4<br>62.2 ± 7.4<br>61.8 ± 7.3	26.5 ± 4.1<br>27.6 ± 7.5<br>27.3 ± 5.6	LW/WW groups: 3 times/week (24-week intervention) initial 15 min at 40–45% HRR, progressing to 50 min at 55–65% HRR (~70% max HR) sessions included warm-up, steady-state walking (with brief intermittent intensity reduction in mid-week session), cool-down<br>CG: No structured exercise (maintained pre-study activity)	①	Male:12<br>Female:39
**Oudegeest-Sander 2013[35]**	NCT01417663	Netherlands; USA	RCT	22	AT：11<br>CON:11	68 ± 3<br>71 ± 5	27.0 ± 2.6<br>24.3 ± 3.3	Exercise groups: 3 times/week (12-month intervention, 45 min/session 10-min warm-up + 30-min cycle ergometer exercise at 70–85% heart rate reserve + 5-min cool-down)<br>No Exercise groups: No structured exercise	②⑦⑧	Male:11<br>Female:11
**Prakhinkit 2014[36]**	none	Thailand	RCT	40	WT:13<br>WT:14<br>CON:13	74.8 ± 1.7<br>74.0 ± 1.9<br>81.0 ± 1.7	81.0 ± 1.7<br>25.5 ± 1.1<br>24.0 ± 1.1	TWE/BWM groups: 3 times/week (12-week intervention, 2 phases) Phase 1 (Weeks 1–6): 20 min/session at 20–39% HRR Phase 2 (Weeks 7–12): 30 min/session at 40–50% HRR (BWM added 500mL water bottles per hand to maintain intensity) both included warm-up/cool-down stretching<br>CON group: No structured exercise	①⑦⑧	Male:0<br>Female:40
**Shiotsu 2018[37]**	none	Japan	RCT	40	CT:15<br>CT:15<br>CON:10	70.4 ± 4.1<br>69.6 ± 4.6<br>71.0 ± 4.4	23.6 ± 3.1<br>24.1 ± 2.1<br>23.8 ± 2.5	AR/RA groups: 2 times/week (10-week intervention) Aerobic exercise: 20 min/session (cycle ergometer, 60% HRR, 50–55 rpm, RPE 12–14) Resistance training: 5 exercises (leg curl/press, chest press, seated row, shoulder press), 3 sets × 8–12 reps, 70–80% 1RM, 1-min rest/set both included warm-up/cool-down<br>CON group: No structured exercise	①②⑥⑧	Male:40<br>Female:0
**Park 2019[38]**	NCT03849300	Korea; USA	RCT	72	WT:35<br>CON:37	70.0 ± 10.0<br>71.0 ± 8.0	20.4 ± 2.1<br>21.3 ± 3.2	AQ group: 4 times/week (12-week intervention, 60 min/session) Warm-up (10 min: underwater stretching + low-intensity gait training) Main exercise (40 min: leg movements + forward/backward/lateral water walking) Cool-down (10 min: same as warm-up) intensity progression: Weeks 1-4 (50-60% HRR, RPE 6-8), Weeks 5-8 (60-70% HRR, RPE 6-8), Weeks 9-12 (70-85% HRR, RPE 6-8)<br>CON group: No structured exercise	⑦⑧⑩⑪	Male:0<br>Female:72
**Park 2023[39]**	KCT0006888	Korea	RCT	29	AT：14<br>CON:15	81.5 ± 4.6<br>82.9 ± 2.7	25.4 ± 2.9<br>25.9 ± 4.0	ADGgroups: 3 times/week (8-week intervention, 45 min/session) 5-min warm-up + 35-min stepping (50–60% HRmax) + 5-min cool-down<br>CG group: No structured exercise	①③⑦⑧	Male:0<br>Female:29

**Figueroa 2014[40]**	none	USA	RCT	25	WBV：13<br>CON:12	55.5 ± 0.7<br>56.4 ± 1.0	33.6 ± 1.3<br>36.0 ± 0.9	WBV group: 3 times/week (12-week intervention) 4 leg exercises (dynamic/static squats, lunges, calf raises) on vibration platform vibration frequency 25–40 Hz, amplitude 1–2 mm 1–6 sets/exercise, 30–60 s/set, 30–60 s rest/set<br>Control group: No structured exercise	②③⑦⑧⑪	Male:0<br>Female:25
**Wong 2018[41]**	NCT03254251	Korea; USA	RCT	41	AT：20<br>CON:21	59 ± 1<br>59 ± 1	24.2 ± 0.8<br>23.8 ± 0.8	SC group: 4 times/week (12-week intervention) progressive training: Weeks 1-2 (2 climbs/day), increased by 1 climb every 3 weeks, Weeks 10-12 (5 climbs/day) each climb: 192 steps (12 flights), 3 sets of 4 flights with 2-min rest/set, 5-min rest between climbs intensity: RPE 11-13 (Borg 6-20 scale)<br>Control group: No structured exercise	③⑦⑧	Male:0<br>Female:41
**Jo 2020[42]**	NCT04042896	Korea	RCT	47	AT：21<br>AT：13<br>CON:13	61.8 ± 10.1<br>57.3 ± 8.4<br>62.5 ± 13.9	27.7 ± 3.0<br>27.0 ± 3.0<br>27.3 ± 4.6	Exergame/Treadmill groups: 3 times/week (12-week intervention, total 50 min/session) 5-min warm-up + 40-min main exercise + 5-min cool-down Exergame: self-selected pace (42–82% HRR)<br>Treadmill: 60–80% HRR<br>Control group: No structured exercise	①⑦⑧	Male:0<br>Female:47
**Jung 2022[43]**	none	Korea	RCT	28	CT：14<br>CON:14	74.64 ± 5.77<br>75.36 ± 4.50	22.58 ± 1.69<br>22.50 ± 1.75	EG group: 3 times/week (12-week intervention, progressive duration) Phase 1 (Weeks 1-2): 25 min/session (10-min warm-up + 10-min main exercise ×2 sets + 5-min rest + 10-min cool-down) Phase 2 (Weeks 3-8): 40 min/session (3 sets main exercise) Phase 3 (Weeks 9-12): 55 min/session (4 sets main exercise) intensity: 60–80% HRR<br>CG group: No structured exercise	③⑦⑧	Male:0<br>Female:28
**Swift 2012[44]**	none	USA	RCT	55	AT：32<br>CON:23	56.3 ± 6.8<br>56.8 ± 5.4	30.7 ± 3.1<br>32.2 ± 3.0	Exercise groups: 3–4 times/week (6-month intervention) intensity: 50% of individual VO2peak mode: alternating semirecumbent cycle ergometer and treadmill initial dose: 4 kcal/kg/week for all, progressive increase to target dose (8/12 kcal/kg/week) for respective groups<br>Control group: No structured exercise	①⑦⑧	Male:0<br>Female:55
**Fetter 2020[45]**	NCT03137849	Brazil	RCT	33	SE：10<br>CON:23	60.4 ± 1.1<br>59.0 ± 1.1	25.9 ± 1.3<br>27.7 ± 0.7	All intervention groups: 2 times/week (12-week intervention, 75 min/session) 60-min main exercise + 15-min supine relaxation<br>No structured exercise control not applicable	①②④⑦⑧	Male:0<br>Female:33
**Figueroa2013[46]**	NCT01371370	USA	RCT	27	RT：14<br>CON：13	54 ± 6<br>54 ± 6	32.6 ± 1.0<br>32.7 ± 1.1	LIRET groups: 3 times/week (12-week intervention) 4 leg exercises (leg press, leg extension, leg flexion, calf raise)<br>Diet group: Hypocaloric diet	②③⑦⑧⑪	Male:0<br>Female:27
**Boutcher 2020[47]**	ACTRN12614001023640	Australia	RCT	60	AT：30<br>CON:30	53.9 ± 3.39<br>53.2 ± 3.07	28.2 ± 3.45<br>27.4 ± 3.51	Ex group: 3 times/week (8-week intervention, total 30 min/session) 5-min warm-up (50–60 RPM) + 20-min ISE (8s maximal sprint [120 RPM] + 12s active recovery) + 5-min cool-down (50–60 RPM)<br>C group: No structured exercise, maintained habitual physical activity	③④⑦⑧	Male:0<br>Female:60

Abbreviations: MBE, Mind-Body Exercise ; SE, Stretching Exercise; RT, Resistance Training; WBV, Whole-Body Vibration ;CON, Non-Exercise Control ; AT,Aerobic Training; WT, Walking Training ; CT,Combined Training ;FMD, Flow-Mediated Dilation; cfPWV, Carotid-Femoral Pulse Wave Velocity; baPWV, Brachial-Ankle Pulse Wave Velocity; AIx, Augmentation Index; AIx@75, Heart Rate-Corrected Augmentation Index ; BSBP, Brachial Systolic Blood Pressure; SBP, Systolic Blood Pressure; DBP, Diastolic Blood Pressure; β-index,Carotid Beta-Stiffness Index (β) ; ABI, Ankle-Brachial Index; faPWV, Femoral-Ankle Pulse Wave Velocity ; BMI, Body Mass Index.

Note: ①Flow-Mediated Dilation; ②Carotid-Femoral Pulse Wave Velocity; ③Brachial-Ankle Pulse Wave Velocity; ④Augmentation Index; ⑤Heart Rate-Corrected Augmentation Index; ⑥Brachial Systolic Blood Pressure; ⑦Systolic Blood Pressure; ⑧Diastolic Blood Pressure; ⑨Carotid Beta-Stiffness Index (β); ⑩Ankle-Brachial Index; ⑪Femoral-Ankle Pulse Wave Velocity.

References

[1] Westhoff TH, Franke N, Schmidt S, et al. Too old to benefit from sports? The cardiovascular effects of exercise training in elderly subjects treated for isolated systolic hypertension. Kidney Blood Press Res. 2007;30(4):240-247. doi:10.1159/000104093

[2] Lee SH, Scott SD, Pekas EJ, Lee S, Lee SH, Park SY. Taekwondo training reduces blood catecholamine levels and arterial stiffness in postmenopausal women with stage-2 hypertension: randomized clinical trial. Clin Exp Hypertens. 2019;41(7):675-681. doi:10.1080/10641963.2018.1539093

[3] Gholami F, Khaki R, Mirzaei B, Howatson G. Resistance training improves nerve conduction and arterial stiffness in older adults with diabetic distal symmetrical polyneuropathy: A randomized controlled trial. Exp Gerontol. 2021;153:111481. doi:10.1016/j.exger.2021.111481

[4] Suboc TB, Strath SJ, Dharmashankar K, et al. Relative importance of step count, intensity, and duration on physical activity's impact on vascular structure and function in previously sedentary older adults. J Am Heart Assoc. 2014;3(1):e000702. Published 2014 Feb 26. doi:10.1161/JAHA.113.000702

[5] Fujie S, Sato K, Miyamoto-Mikami E, et al. Reduction of arterial stiffness by exercise training is associated with increasing plasma apelin level in middle-aged and older adults. PLoS One. 2014;9(4):e93545. Published 2014 Apr 1. doi:10.1371/journal.pone.0093545

[6] Shimomura M, Fujie S, Sanada K, Kajimoto H, Hamaoka T, Iemitsu M. Relationship between plasma asymmetric dimethylarginine and nitric oxide levels affects aerobic exercise training-induced reduction of arterial stiffness in middle-aged and older adults. Phys Act Nutr. 2021;25(1):16-22. doi:10.20463/pan.2021.0003

[7] Ploydang T, Khovidhunkit W, Tanaka H, Suksom D. Nordic Walking in Water on Cerebrovascular Reactivity and Cognitive Function in Elderly Patients with Type 2 Diabetes. Med Sci Sports Exerc. 2023;55(10):1803-1811. doi:10.1249/MSS.0000000000003216

[8] Otsuki T, Nakamura F, Zempo-Miyaki A. Nitric Oxide and Decreases in Resistance Exercise Blood Pressure With Aerobic Exercise Training in Older Individuals. Front Physiol. 2019;10:1204. Published 2019 Sep 20. doi:10.3389/fphys.2019.01204

[9] Novaković M, Krevel B, Rajkovič U, et al. Moderate-pain versus pain-free exercise, walking capacity, and cardiovascular health in patients with peripheral artery disease. J Vasc Surg. 2019;70(1):148-156. doi:10.1016/j.jvs.2018.10.109

[10] Yasuda T, Fukumura K, Fukuda T, et al. Muscle size and arterial stiffness after blood flow-restricted low-intensity resistance training in older adults. Scand J Med Sci Sports. 2014;24(5):799-806. doi:10.1111/sms.12087

[11] Miura H, Nakagawa E, Takahashi Y. Influence of group training frequency on arterial stiffness in elderly women. Eur J Appl Physiol. 2008;104(6):1039-1044. doi:10.1007/s00421-008-0860-1

[12] Fujie S, Horii N, Kajimoto H, et al. Impact of resistance training and chicken intake on vascular and muscle health in elderly women. J Cachexia Sarcopenia Muscle. 2025;16(1):10.1002/jcsm.13572. doi:10.1002/jcsm.13572

[13] Jaime SJ, Maharaj A, Alvarez-Alvarado S, Figueroa A. Impact of low-intensity resistance and whole-body vibration training on aortic hemod

ynamics and vascular function in postmenopausal women. Hypertens Res. 2019;42(12):1979-1988. doi:10.1038/s41440-019-0328-1

[14] Bellia A, Iellamo F, De Carli E, et al. Exercise individualized by TRIMPi method reduces arterial stiffness in early onset type 2 diabetic patients: A randomized controlled trial with aerobic interval training. Int J Cardiol. 2017;248:314-319. doi:10.1016/j.ijcard.2017.06.065

[15] Kim HB, Seo MW, Jung HC. Effects of Aerobic vs. Resistance Exercise on Vascular Function and Vascular Endothelial Growth Factor in Older Women. Healthcare (Basel). 2023;11(18):2479. Published 2023 Sep 7. doi:10.3390/healthcare11182479

[16] Ohta M, Hirao N, Mori Y, et al. Effects of bench step exercise on arterial stiffness in post-menopausal women: contribution of IGF-1 bioactivity and nitric oxide production. Growth Horm IGF Res. 2012;22(1):36-41. doi:10.1016/j.ghir.2011.12.004

[17] Bouaziz W, Lang PO, Schmitt E, et al. Effects of a short-term interval aerobic training program with recovery bouts on vascular function in sedentary aged 70 or over: A randomized controlled trial. Arch Gerontol Geriatr. 2019;82:217-225. doi:10.1016/j.archger.2019.02.017

[18] Lai CL, Chen HY, Tseng SY, et al. Effect of whole-body vibration for 3 months on arterial stiffness in the middle-aged and elderly. Clin Interv Aging. 2014;9:821-828. Published 2014 May 12. doi:10.2147/CIA.S60029

[19] Kitzman DW, Brubaker PH, Herrington DM, et al. Effect of endurance exercise training on endothelial function and arterial stiffness in older patients with heart failure and preserved ejection fraction: a randomized, controlled, single-blind trial. J Am Coll Cardiol. 2013;62(7):584-592. doi:10.1016/j.jacc.2013.04.033

[20] Patil SG, Aithala MR, Das KK. Effect of yoga on arterial stiffness in elderly subjects with increased pulse pressure: A randomized controlled study. Complement Ther Med. 2015;23(4):562-569. doi:10.1016/j.ctim.2015.06.002

[21] Oliveira NL, Ribeiro F, Silva G, et al. Effect of exercise-based cardiac rehabilitation on arterial stiffness and inflammatory and endothelial dysfunction biomarkers: a randomized controlled trial of myocardial infarction patients. Atherosclerosis. 2015;239(1):150-157. doi:10.1016/j.atherosclerosis.2014.12.057

[22] Kobayashi R, Asaki K, Hashiguchi T, Negoro H. Effect of aerobic exercise training frequency on arterial stiffness in middle-aged and elderly females. J Phys Ther Sci. 2022;34(5):347-352. doi:10.1589/jpts.34.347

[23] Otsuki T, Namatame H, Yoshikawa T, Zempo-Miyaki A. Combined aerobic and low-intensity resistance exercise training increases basal nitric oxide production and decreases arterial stiffness in healthy older adults. J Clin Biochem Nutr. 2020;66(1):62-66. doi:10.3164/jcbn.19-81

[24] Figueroa A, Park SY, Seo DY, Sanchez-Gonzalez MA, Baek YH. Combined resistance and endurance exercise training improves arterial stiffness, blood pressure, and muscle strength in postmenopausal women. Menopause. 2011;18(9):980-984. doi:10.1097/gme.0b013e3182135442

[25] Son WM, Sung KD, Cho JM, Park SY. Combined exercise reduces arterial stiffness, blood pressure, and blood markers for cardiovascular risk in postmenopausal women with hypertension. Menopause. 2017;24(3):262-268. doi:10.1097/GME.0000000000000765

[26] Shin JH, Lee Y, Kim SG, Choi BY, Lee HS, Bang SY. The beneficial effects of Tai Chi exercise on endothelial function and arterial stiffne

ss in elderly women with rheumatoid arthritis. Arthritis Res Ther. 2015;17:380. Published 2015 Dec 24. doi:10.1186/s13075-015-0893-x
[27] Kim HK, Hwang CL, Yoo JK, et al. All-Extremity Exercise Training Improves Arterial Stiffness in Older Adults. Med Sci Sports Exerc. 2017;49(7):1404-1411. doi:10.1249/MSS.0000000000001229
[28] Hasegawa N, Fujie S, Horii N, et al. Aerobic exercise training-induced changes in serum C1q/TNF-related protein levels are associated with reduced arterial stiffness in middle-aged and older adults. Am J Physiol Regul Integr Comp Physiol. 2018;314(1):R94-R101. doi:10.1152/ajpregu.00212.2017
[29] Madden KM, Lockhart C, Cuff D, Potter TF, Meneilly GS. Aerobic training-induced improvements in arterial stiffness are not sustained in older adults with multiple cardiovascular risk factors. J Hum Hypertens. 2013;27(5):335-339. doi:10.1038/jhh.2012.38
[30] 崔永胜,牛爱军. 24周太极拳运动对中老年人AIP、ABI和PWV的影响[J].湖州师范学院学报,2019,41(08):94-99.
[31] Pierce GL, Eskurza I, Walker AE, Fay TN, Seals DR. Sex-specific effects of habitual aerobic exercise on brachial artery flow-mediated dilation in middle-aged and older adults. Clin Sci (Lond). 2011;120(1):13-23. doi:10.1042/CS20100174
[32] Park W, Jung WS, Hong K, Kim YY, Kim SW, Park HY. Effects of Moderate Combined Resistance- and Aerobic-Exercise for 12 Weeks on Body Composition, Cardiometabolic Risk Factors, Blood Pressure, Arterial Stiffness, and Physical Functions, among Obese Older Men: A Pilot Study. Int J Environ Res Public Health. 2020;17(19):7233. Published 2020 Oct 3. doi:10.3390/ijerph17197233
[33] Rech A, Botton CE, Lopez P, Quincozes-Santos A, Umpierre D, Pinto RS. Effects of short-term resistance training on endothelial function and inflammation markers in elderly patients with type 2 diabetes: A randomized controlled trial. Exp Gerontol. 2019;118:19-25. doi:10.1016/j.exger.2019.01.003
[34] Haynes A, Naylor LH, Spence AL, et al. Effects of Land versus Water Walking Interventions on Vascular Function in Older Adults. Med Sci Sports Exerc. 2021;53(1):83-89. doi:10.1249/MSS.0000000000002439
[35] Oudegeest-Sander MH, Olde Rikkert MG, Smits P, et al. The effect of an advanced glycation end-product crosslink breaker and exercise training on vascular function in older individuals: a randomized factorial design trial. Exp Gerontol. 2013;48(12):1509-1517. doi:10.1016/j.exger.2013.10.009
[36] Prakhinkit S, Suppapitiporn S, Tanaka H, Suksom D. Effects of Buddhism walking meditation on depression, functional fitness, and endothelium-dependent vasodilation in depressed elderly. J Altern Complement Med. 2014;20(5):411-416. doi:10.1089/acm.2013.0205
[37] Shiotsu Y, Watanabe Y, Tujii S, Yanagita M. Effect of exercise order of combined aerobic and resistance training on arterial stiffness in older men. Exp Gerontol. 2018;111:27-34. doi:10.1016/j.exger.2018.06.020
[38] Park SY, Kwak YS, Pekas EJ. Impacts of aquatic walking on arterial stiffness, exercise tolerance, and physical function in patients with peripheral artery disease: a randomized clinical trial. J Appl Physiol (1985). 2019;127(4):940-949. doi:10.1152/japplphysiol.00209.2019
[39] Park W, Lee J, Hong K, et al. Protein-Added Healthy Lunch-Boxes Combined with Exercise for Improving Physical Fitness and Vascular Function in Pre-Frail Older Women: A Community-Based Randomized Controlled Trial. Clin Interv Aging. 2023;18:13-27. Published 2023 Jan 5. doi:

10.2147/CIA.S391700

[40] Figueroa A, Kalfon R, Madzima TA, Wong A. Whole-body vibration exercise training reduces arterial stiffness in postmenopausal women with prehypertension and hypertension. Menopause. 2014;21(2):131-136. doi:10.1097/GME.0b013e318294528c

[41] Wong A, Figueroa A, Son WM, Chernykh O, Park SY. The effects of stair climbing on arterial stiffness, blood pressure, and leg strength in postmenopausal women with stage 2 hypertension. Menopause. 2018;25(7):731-737. doi:10.1097/GME.0000000000001072

[42] Jo EA, Wu SS, Han HR, Park JJ, Park S, Cho KI. Effects of exergaming in postmenopausal women with high cardiovascular risk: A randomized controlled trial. Clin Cardiol. 2020;43(4):363-370. doi:10.1002/clc.23324

[43] Jung WS, Kim YY, Kim JW, Park HY. Effects of Circuit Training Program on Cardiovascular Risk Factors, Vascular Inflammatory Markers, and Insulin-like Growth Factor-1 in Elderly Obese Women with Sarcopenia. Rev Cardiovasc Med. 2022;23(4):134. Published 2022 Apr 8. doi:10.31083/j.rcm2304134

[44] Swift DL, Earnest CP, Blair SN, Church TS. The effect of different doses of aerobic exercise training on endothelial function in postmenopausal women with elevated blood pressure: results from the DREW study. Br J Sports Med. 2012;46(10):753-758. doi:10.1136/bjsports-2011-090025

[45] Fetter C, Marques JR, de Souza LA, et al. Additional Improvement of Respiratory Technique on Vascular Function in Hypertensive Postmenopausal Women Following Yoga or Stretching Video Classes: The YOGINI Study. Front Physiol. 2020;11:898. Published 2020 Aug 27. doi:10.3389/fphys.2020.00898

[46] Figueroa A, Vicil F, Sanchez-Gonzalez MA, et al. Effects of diet and/or low-intensity resistance exercise training on arterial stiffness, adiposity, and lean mass in obese postmenopausal women. Am J Hypertens. 2013;26(3):416-423. doi:10.1093/ajh/hps050

[47] Ho TY, Redmayne GP, Tran A, et al. The effect of interval sprinting exercise on vascular function and aerobic fitness of post-menopausal women. Scand J Med Sci Sports. 2020;30(2):312-321. doi:10.1111/sms.13574

Appendix 3: List of data extracted from the included randomized clinical trials

Data category	List of variables
Study	Primary author, year of publication, study duration, total number of patients in each group, study source,country/region, trial registration number, study design
Patients	Sex, age, systolic blood pressure, diastolic blood pressure,baseline BMI,exclusion of exercise contraindications,smoking/alcohol consumption status
Interventions	Exercise type , exercise frequency, single exerciseduration, exercise intensity control , supervision mode, core exercise content, progressive training strategy, control group intervention, compliance monitoring method
Efficacy outcome	Mean of change in FMD(%), cfPWV(m/s),baPWV(m/s),AIx(%),AIx@75(%),BSBP(mmHg)，SBP(mmHg),DBP(mmHg), faPWV(m/s), BMI，with respective standard deviation from baseline
Adverse events	Types of exercise-related adverse events

FMD, Flow-Mediated Dilation; cfPWV, Carotid-Femoral Pulse Wave Velocity; baPWV, Brachial-Ankle Pulse Wave Velocity; AIx, Augmentation Index; AIx@75, Heart Rate-Corrected Augmentation Index ; BSBP, Brachial Systolic Blood Pressure; SBP, Systolic Blood Pressure; DBP, Diastolic Blood Pressure; faPWV, Femoral-Ankle Pulse Wave Velocity; BMI, Body Mass Index

Appendix 4: Risk of bias of randomized clinical trials

Figure S4: Overall risk of bias presented as percentage of each risk of bias item across all included studies. Green = Low risk, Red = High risk, Yellow = Some concerns.

Risk of bias of randomized clinical trials

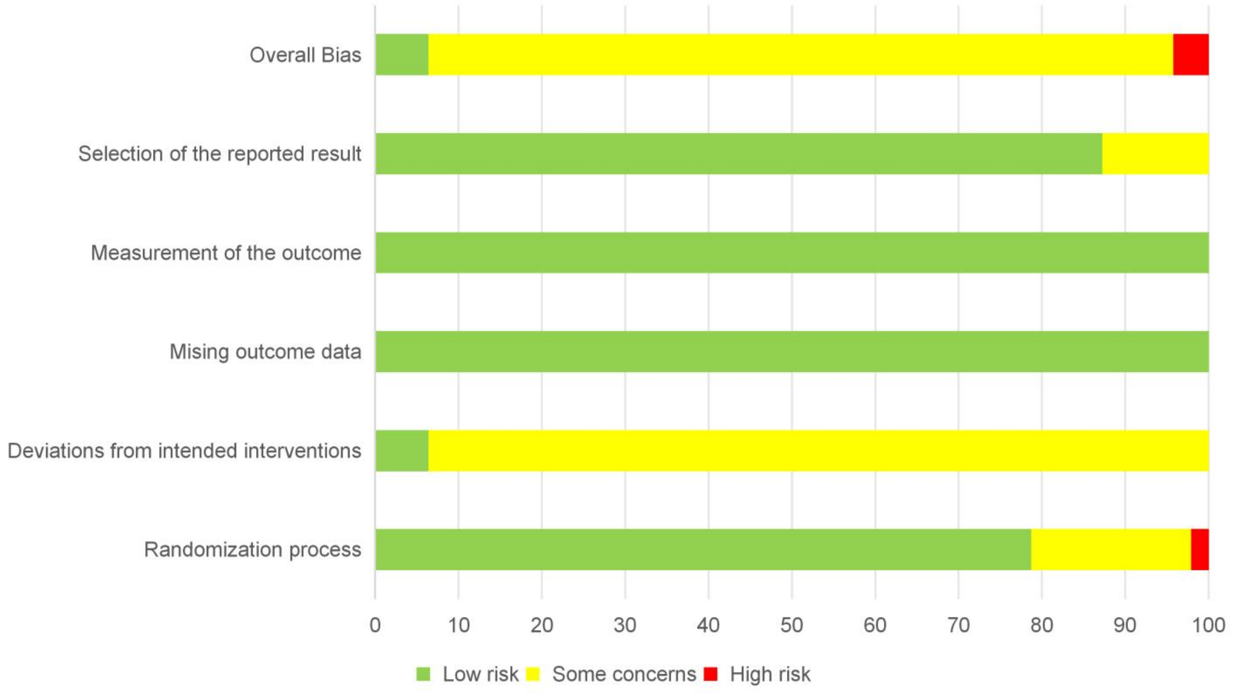

Table S4: Study level risk of bias assessment using Cochrane risk of bias tool 2.0 for assessing risk of bias of randomized clinical trials.

Unique ID	Study ID	Randomization process	Deviations from intended interventions	Mising outcome data	Measurement of the outcome	Selection of the reported result	Over all
**Westhoff2007**	NCT00315224	Low	Some concerns	Low	Low	Low	Some concerns
**Lee2019**	NCT03544307	Low	Some concerns	Low	Low	Low	Some concerns
**Gholami2021**	IRCT20170120032066N4	Low	Some concerns	Low	Low	Low	Some concerns
**Suboc2014**	NCT01212978	Low	Some concerns	Low	Low	Low	Some concerns
**Fujie2014**	none	Some concerns	Some concerns	Low	Low	Some concerns	High risk
**Shimomura2021**	UMIN000035520	Low	Some concerns	Low	Low	Low	Some concerns
**Ploydang2023**	none	Low	Some concerns	Low	Low	Low	Some concerns
**Otsuki2019**	none	High risk	Some concerns	Low	Low	Some concerns	High risk
**Novakovic2019**	NCT02642276	Low	Low	Low	Low	Low	Low
**Yasuda2013**	none	Low	Some concerns	Low	Low	Some concerns	Some concerns
**Miura2008**	none	Low	Some concerns	Low	Low	Low	Some concerns
**Fujie2025**	UMIN000038253	Low	Some concerns	Low	Low	Low	Some concerns
**Jaime2019**	none	Low	Some concerns	Low	Low	Low	Some concerns
**Bellia2017**	none	Low	Some concerns	Low	Low	Low	Some concerns
**Kim2023**	none	Low	Some concerns	Low	Low	Low	Some concerns
**Ohta2012**	none	Low	Some concerns	Low	Low	Low	Some concerns
**Bouaziz2019**	NCT02263573	Low	Some concerns	Low	Low	Low	Some concerns
**Lai2014**	none	Low	Some concerns	Low	Low	Some concerns	Some concerns
**Kitzman2013**	NCT01113840	Some concerns	Some concerns	Low	Low	Low	Some concerns
**Patil2015**	CTRI/2011/10/002077	Low	Low	Low	Low	Low	Low
**Oliveira2015**	NCT01432639	Some concerns	Some concerns	Low	Low	Low	Some concerns
**Kobayashi2022**	none	Low	Some concerns	Low	Low	Low	Some concerns
**Otsuki2020**	none	Low	Some concerns	Low	Low	Low	Some concerns
**Figueroa2011**	none	Low	Some concerns	Low	Low	Low	Some concerns
**Son2017**	none	Low	Some concerns	Low	Low	Low	Some concerns
**Shin2015**	none	Low	Some concerns	Low	Low	Low	Some concerns
**Kim2017**	none	Low	Some concerns	Low	Low	Low	Some concerns
**Hasegawa2018**	none	Some concerns	Some concerns	Low	Low	Low	Some concerns
**Madden2013**	none	Low	Some concerns	Low	Low	Low	Some concerns
**Cui2019**	none	Low	Some concerns	Low	Low	Low	Some concerns
**Pierce2011**	none	Low	Some concerns	Low	Low	Low	Some concerns
**Park2020**	none	Some concerns	Some concerns	Low	Low	Low	Some concerns
**Rech2019**	NCT02548000	Low	Some concerns	Low	Low	Low	Some concerns
**Haynes2021**	ACTRN12614000017628	Low	Some concerns	Low	Low	Low	Some concerns
**Oudegeest-Sander2013**	NCT01417663	Some concerns	Some concerns	Low	Low	Low	Some concerns
**Prakhinkit2014**	none	Low	Some concerns	Low	Low	Some concerns	Some concerns
**Shiotsu2018**	none	Low	Some concerns	Low	Low	Low	Some concerns
**Park2019**	NCT03849300	Low	Some concerns	Low	Low	Low	Some concerns
**Park2023**	KCT0006888	Some concerns	Some concerns	Low	Low	Low	Some concerns

**Figueroa2014**	none	Low	Some concerns	Low	Low	Some concerns	Some concerns
**Wong2018**	NCT03254251	Low	Some concerns	Low	Low	Low	Some concerns
**Jo2020**	NCT04042896	Low	Some concerns	Low	Low	Low	Some concerns
**Jung2022**	none	Some concerns	Some concerns	Low	Low	Low	Some concerns
**Swift2012**	none	Low	Low	Low	Low	Low	Low
**Fetter2020**	NCT03137849	Low	Some concerns	Low	Low	Low	Some concerns
**Figueroa2013**	NCT01371370	Low	Some concerns	Low	Low	Low	Some concerns
**Boutcher2020**	ACTRN12614001023640	Some concerns	Some concerns	Low	Low	Low	Some concerns

Appendix 5: Evaluation of inconsistency and heterogeneity

Table S5.1: Global consistency

**Clinical outcome**	**Chi square**	**P value**	τ^2
FMD	2.44	0.1179	2.4445
cfPWV	0.14	0.7049	1.7025
baPWV	25.28	0.2438	＜0.01
AIx	0.06	0.7989	0.5142
AIx@75	4.83	0.0281	0.5307
BSBP	0.03	0.8677	＜0.01
SBP	14.93	0.0019	0.2154
DBP	0.49	0.7809	0.3681
β-index	3.42	0.0645	0.8140
ABI	0.13	0.7194	＜0.01
faPWV	38.43	0.3359	0.0167
BMI	0.27	0.6014	＜0.01

Abbreviations: FMD, Flow-Mediated Dilation; cfPWV, Carotid-Femoral Pulse Wave Velocity; baPWV, Brachial-Ankle Pulse Wave Velocity; AIx, Augmentation Index; AIx@75, Heart Rate-Corrected Augmentation Index ; BSBP, Brachial Systolic Blood Pressure; SBP, Systolic Blood Pressure; DBP, Diastolic Blood Pressure; β -index,Carotid Beta-Stiffness Index (β) ; ABI, Ankle-Brachial Index; faPWV, Femoral-Ankle Pulse Wave Velocity ; BMI, Body Mass Index.

Table S5.2: Side-splitting of FMD. Inconsistency test between direct and indirect treatment comparisons in mixed treatment comparison.

Comparison	Direct		Indirect		Difference		
	Coef.	Std. Err.	Coef.	Std. Err.	Coef.	Std. Err.	P>|z|
MBEvsCON	−2.43	1.717056	−1.064891	24.25705	−1.365109	24.31774	0.955
RTvsCON	−5.039614	1.35959	0.1962949	141.4745	−5.235908	141.4854	0.97
WBVvsCON	−5.7	1.70828	0.8555877	200.0578	−6.555588	200.0674	0.974
CONvsAT	1.263817	0.6298412	4.820501	2.182605	−3.556684	2.273103	0.118
CONvsWT	3.519887	0.7563594	−0.0309156	2.142813	3.550802	2.273857	0.118
CONvsCT	0.2	1.567544	5.047816	200.0335	−4.847816	200.0396	0.981
ATvsWT	−1.299934	2.048714	2.256194	0.9876874	−3.556128	2.274365	0.118

Abbreviations:FMD, Flow-Mediated Dilation; MBE, Mind-Body Exercise ; CON, Non-Exercise Control ; RT, Resistance Training; WBV, Whole-Body Vibration ;AT,Aerobic Training; WT, Walking Training ; CT,Combined Training.

Table S5.3: Side-splitting of cfPWV. Inconsistency test between direct and indirect treatment comparisons in mixed treatment comparison.

Comparison	Direct		Indirect		Difference		
	Coef.	Std. Err.	Coef.	Std. Err.	Coef.	Std. Err.	P>|z|
SEvsCON	0.4791489	1.512311	1.308713	1.598815	-0.8295641	2.200748	0.706
SEvsAT	0.4263784	1.475978	-0.4073291	1.632836	0.8337075	2.20106	0.705
RTvsCON	0.2966695	1.383494	1.428918	200.0433	-1.132249	200.0491	0.995
WBVvsCON	0.5385628	0.972567	1.187061	141.4608	-0.648498	141.4648	0.996
CONvsAT	-0.8855475	0.6205134	-0.0525085	2.112799	-0.833039	2.20204	0.705
CONvsWT	-0.9524354	1.396619	-2.678082	200.0457	1.725646	200.0493	0.993
CONvsCT	-0.6034077	1.370581	-2.329052	200.0267	1.725644	200.0305	0.993

Abbreviations: cfPWV, Carotid-Femoral Pulse Wave Velocity; SE, Stretching Exercise;CON, Non-Exercise Control ;AT,Aerobic Training; RT, Resistance Training; WBV, Whole-Body Vibration; WT, Walking Training ; CT,Combined Training

Table S5.4: Side-splitting of AIx@75. Inconsistency test between direct and indirect treatment comparisons in mixed treatment comparison.

Comparison	Direct		Indirect		Difference		
	Coef.	Std. Err.	Coef.	Std. Err.	Coef.	Std. Err.	P>|z|
SEvsCON	-10.6	5.923522	4.752138	3.665389	-15.35214	6.965858	0.028
SEvsAT	3.069999	3.00597	-12.18623	6.25911	15.25623	6.943509	0.028
RTvsCON	-2.9	8.610466	-5.425943	201.0679	2.525943	201.3191	0.99
WBVvsCON	3.9	8.500028	-12.30465	201.066	16.20465	201.3025	0.936
CONvsAT	-1.700641	2.110225	13.58906	6.62293	-15.2897	6.951403	0.028

Abbreviations: AIx@75, Heart Rate-Corrected Augmentation Index ; SE, Stretching Exercise; CON, Non-Exercise Control ; AT,Aerobic Training; RT, Resistance Training; WBV, Whole-Body Vibration ;

Table S5.5: Side-splitting of SBP. Inconsistency test between direct and indirect treatment comparisons in mixed treatment comparison.

Comparison	Direct		Indirect		Difference		
	Coef.	Std. Err.	Coef.	Std. Err.	Coef.	Std. Err.	P>|z|
MBEvsCON	2.7	5.968759	0.7951647	16.03535	1.904835	17.11019	0.911
SEvsCON	-7.905135	6.394287	19.84509	3.933896	-27.75023	7.509027	0.9
SEvsAT	14.22954	3.670807	-13.50517	6.539285	27.73471	7.499477	0.7
RTvsCON	1.409429	3.07553	-7.789951	7.059655	9.199381	7.70068	0.232
RTvsAT	-11.99194	6.894362	-2.703505	3.477014	-9.288437	7.722123	0.229
WBVvsCON	2.654112	4.056743	2.280716	116.1419	0.3733957	116.2971	0.997
CONvsAT	-5.090223	1.739612	-0.9933253	4.353865	-4.096898	4.69012	0.382
CONvsWT	-3.855206	3.096977	-2.463973	6.485179	-1.391233	7.185452	0.846
CONvsCT	-5.857959	1.880514	-10.97109	61.42341	5.113132	61.42378	0.934
ATvsWT	1.997536	6.275855	0.5804009	3.531285	1.417135	7.200703	0.844

Abbreviations:SBP, Systolic Blood Pressure; MBE, Mind-Body Exercise ; CON, Non-Exercise Control ; SE, Stretching Exercise; AT,Aerobic Training; RT, Resistance Training; WBV, Whole-Body Vibration ;WT, Walking Training ; CT,Combined Training

Table S5.6: Side-splitting of DBP. Inconsistency test between direct and indirect treatment comparisons in mixed treatment comparison.

Comparison	Direct		Indirect		Difference		
	Coef.	Std. Err.	Coef.	Std. Err.	Coef.	Std. Err.	P>|z|
MBEvsCON	0.3	3.21032	0.0641168	15.43752	0.2358832	15.76779	0.988
SEvsCON	-0.8702051	4.335379	2.145821	3.032005	-3.016026	5.290934	0.569
SEvsAT	0.0999744	2.904	-2.915584	4.419574	3.015559	5.2884	0.569
RTvsCON	0.0009437	2.653209	-2.100889	3.966887	2.101833	4.773548	0.66
RTvsAT	-3.999463	3.872431	-1.892084	2.793349	-2.107379	4.77504	0.659
WBVvsCON	1.006848	2.285003	-0.4306221	115.6865	1.43747	115.7347	0.99
CONvsAT	-1.974923	0.8934265	-1.784815	3.485922	-0.1901077	3.598742	0.958
CONvsWT	-2.164616	1.830129	-2.747386	100.232	0.5827705	100.2323	0.995
CONvsCT	-3.477409	1.043531	-4.064827	58.09813	0.587417 6	58.09851	0.992

Abbreviations:DBP, Diastolic Blood Pressure; MBE, Mind-Body Exercise ; CON, Non-Exercise Control ; SE, Stretching Exercise; AT,Aerobic Training; RT, Resistance Training; WBV, Whole-Body Vibration ;WT, Walking Training ; CT,Combined Training

Table S5.7: Side-splitting of BMI. Inconsistency test between direct and indirect treatment comparisons in mixed treatment comparison.

Comparison	Direct		Indirect		Difference		
	Coef.	Std. Err.	Coef.	Std. Err.	Coef.	Std. Err.	P>|z|
MBEvsCON	0.00000000001	0.7884584	0.087667	17.67942	-0.087667	17.69699	0.996
SEvsCON	-0.48	1.501146	0.4804043	200.0133	-0.9604043	200.0302	0.996
RTvsCON	0.7737185	0.2707614	0.0433358	1.371354	0.7303827	1.397835	0.601
RTvsAT	-0.1000372	1.350288	0.6302465	0.3621859	-0.7302837	1.39802	0.601
WBVvsCON	0.3	1.560865	-0.2996958	200.0183	0.5996958	200.0365	0.998
CONvsAT	-0.1434749	0.2405552	-0.873182	1.376708	0.729707	1.397537	0.602
CONvsWT	0.4093528	0.5187829	0.4090002	100.0286	0.0003526	100.0272	1
CONvsCT	-0.6766884	0.2127147	-0.6770371	75.60408	0.000348	775.60378	1

Abbreviations: BMI, Body Mass Index; MBE, Mind-Body Exercise ; CON, Non-Exercise Control ;SE, Stretching Exercise; RT, Resistance Training;AT,Aerobic Training; WBV, Whole-Body Vibration ; WT, Walking Training ; CT,Combined Training

Appendix 6: Network maps and forest plots of secondary outcomes

Figure S6.1: Network map of the effect on Augmentation Index, and forest plot of network effect sizes for compared with placebo.The size of the nodes was proportional to the number of participants included in the trial, and the thickness of lines between the interventions relates to the number of studies for that comparison.

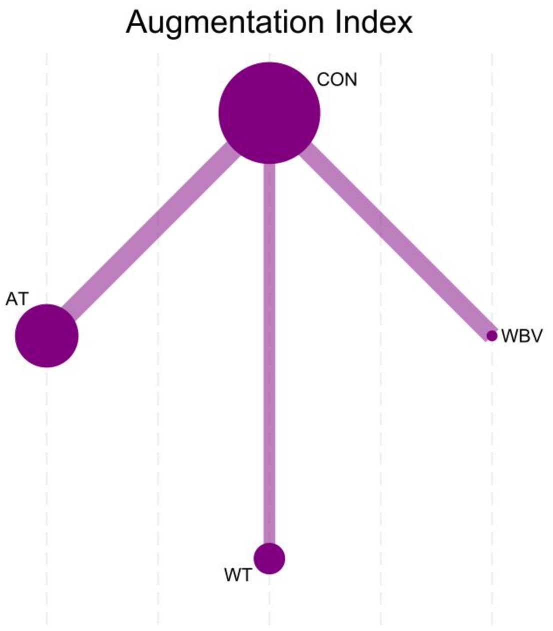

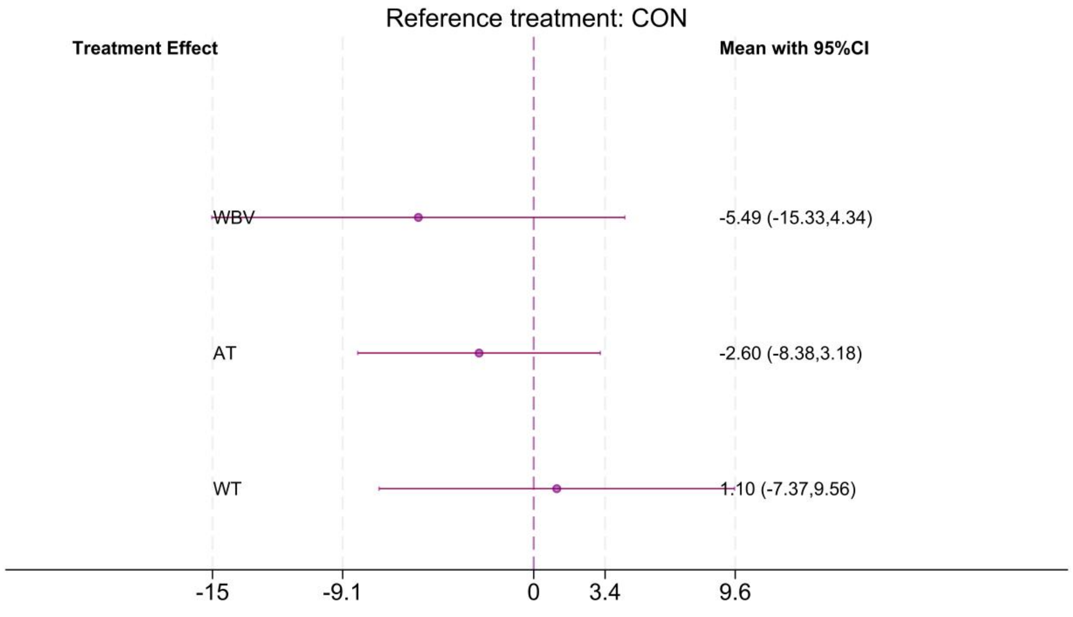

Figure S6.2: Network map of the effect on Heart Rate-Corrected Augmentation Index, and forest plot of network effect sizes for compared with placebo.The size of the nodes was proportional to the number of participants included in the trial, and the thickness of lines between the interventions relates to the number of studies for that comparison.

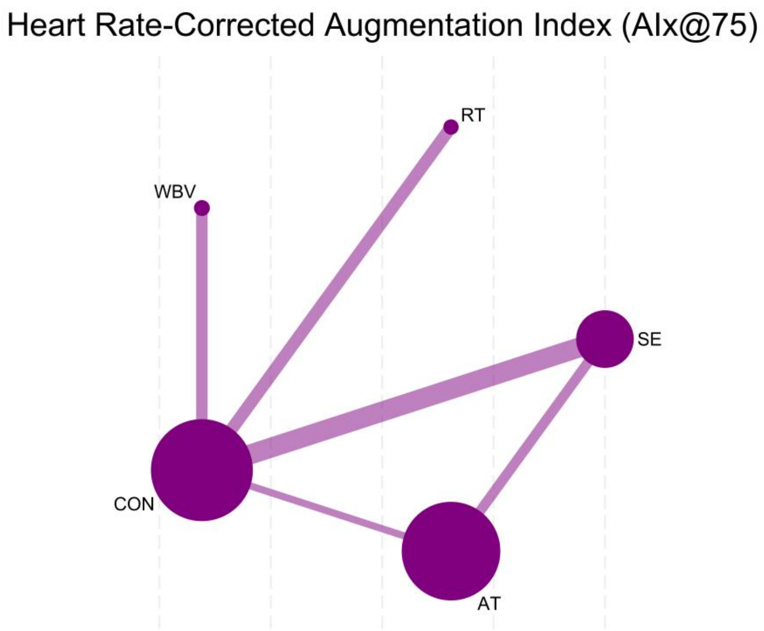

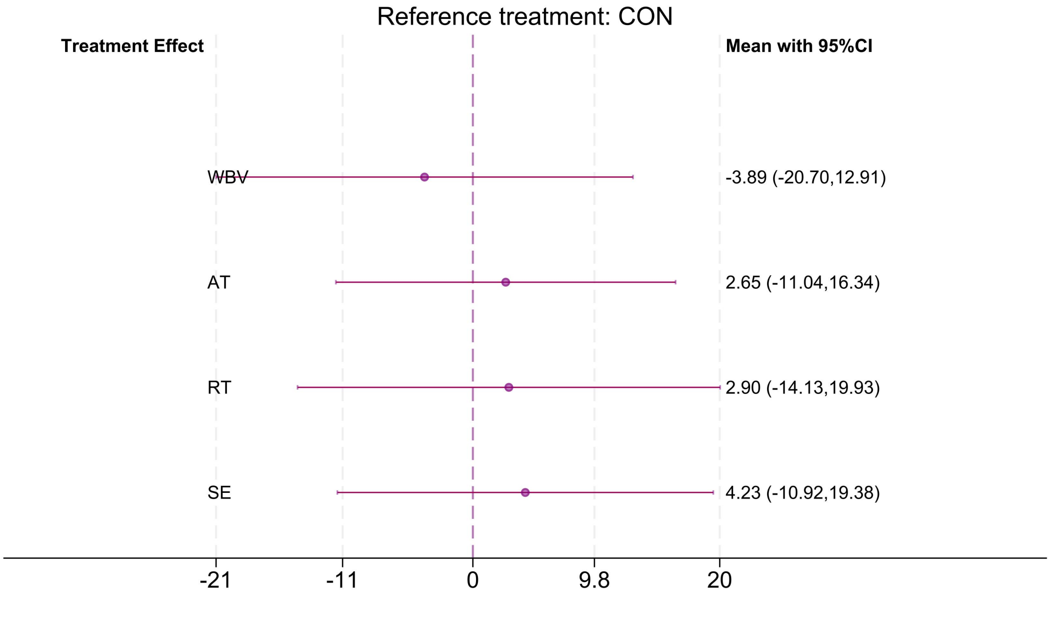

Figure S6.3: Network map of the effect on Brachial Systolic Blood Pressure, and forest plot of network effect sizes for compared with placebo.The size of the nodes was proportional to the number of participants included in the trial, and the thickness of lines between the interventions relates to the number of studies for that comparison.

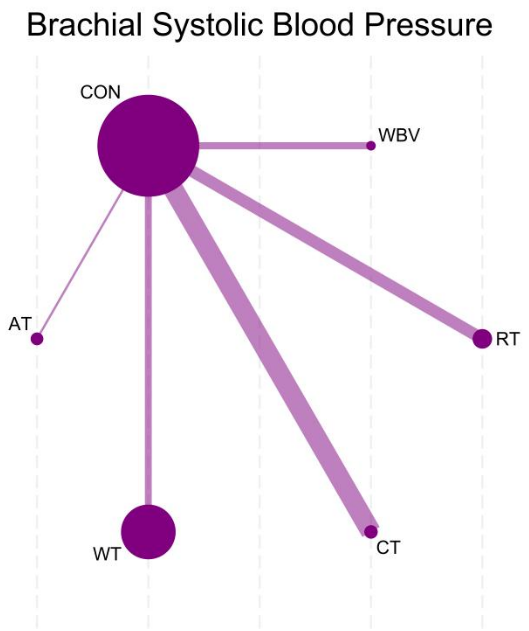

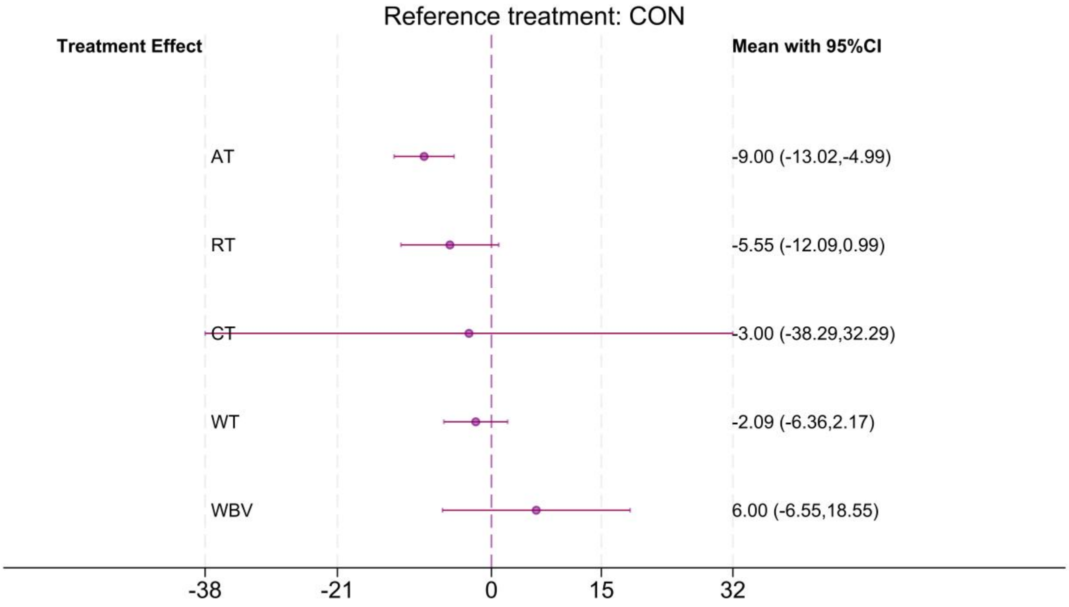

Figure S6.4: Network map of the effect on Carotid Beta-Stiffness Index (β), and forest plot of network effect sizes for compared with placebo.The size of the nodes was proportional to the number of participants included in the trial, and the thickness of lines between the interventions relates to the number of studies for that comparison.

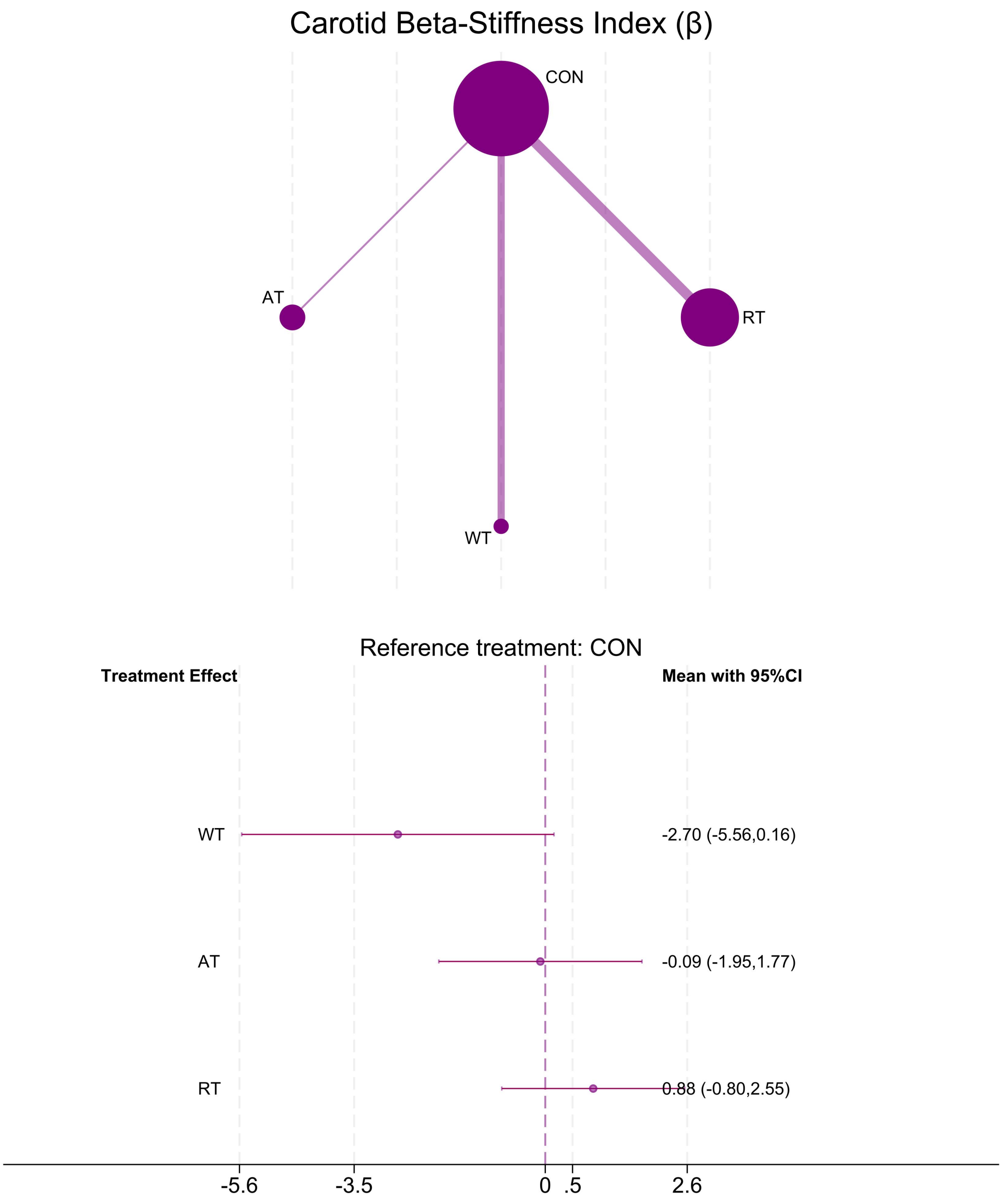

Figure S6.5: Network map of the effect on Ankle-Brachial Index, and forest plot of network effect sizes for compared with placebo.The size of the nodes was proportional to the number of participants included in the trial, and the thickness of lines between the interventions relates to the number of studies for that comparison.

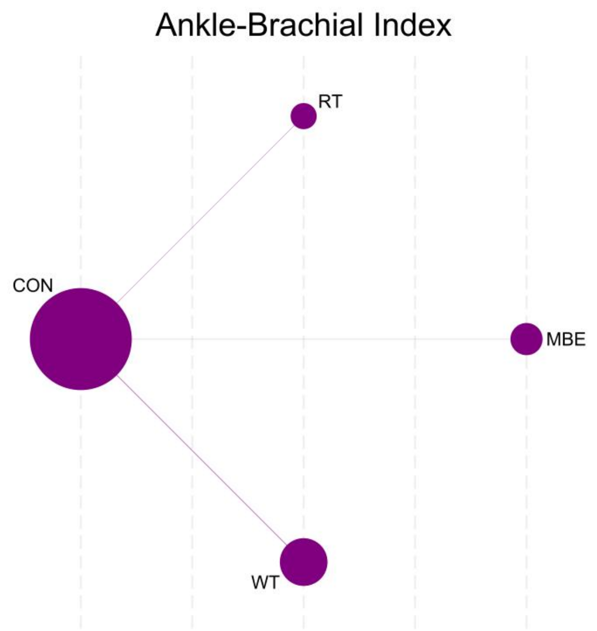

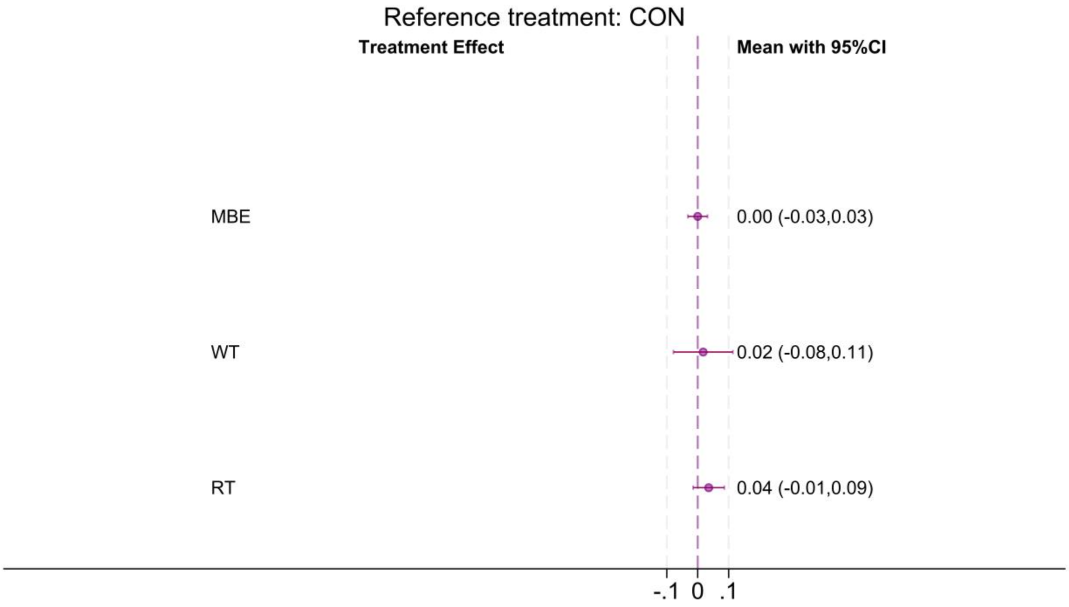

Figure S6.6: Network map of the effect on Femoral-Ankle Pulse Wave Velocity, and forest plot of network effect sizes for compared with placebo.The size of the nodes was proportional to the number of participants included in the trial, and the thickness of lines between the interventions relates to the number of studies for that comparison.

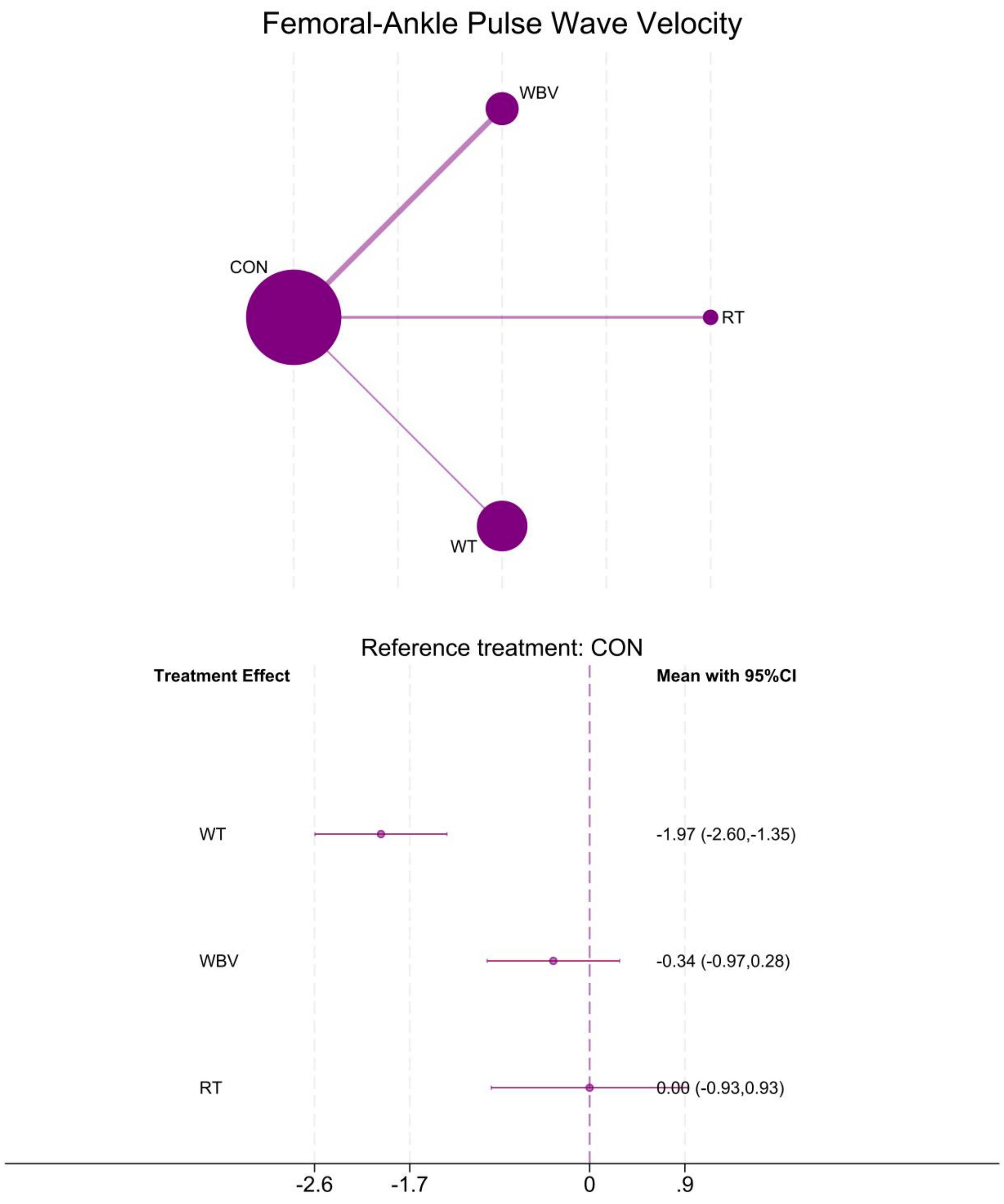

Figure S6.7: Network map of the effect on BMI, and forest plot of network effect sizes for compared with placebo.The size of the nodes was proportional to the number of participants included in the trial, and the thickness of lines between the interventions relates to the number of studies for that comparison.

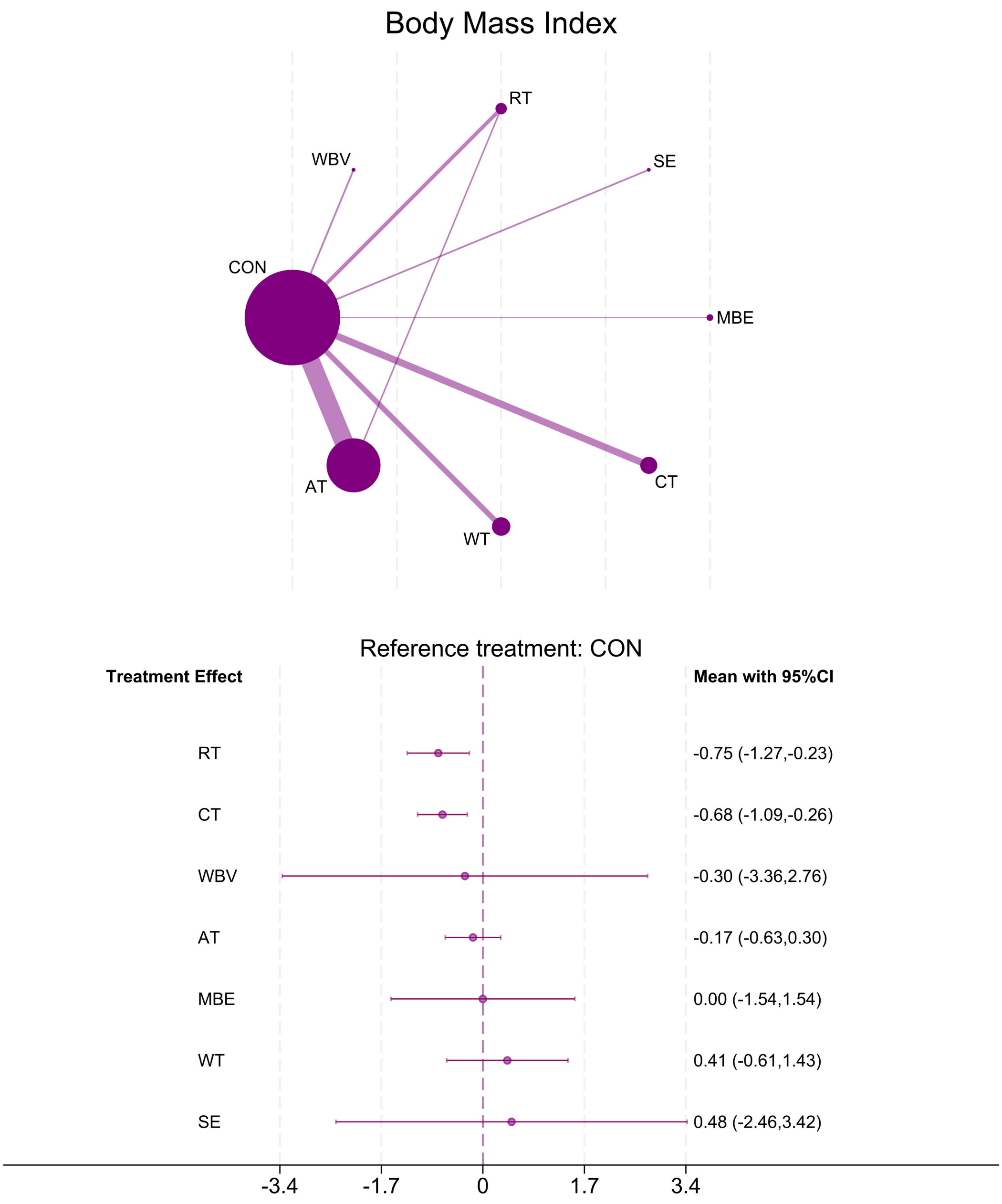

Appendix 7: SUCRA and cumulative probability plots

Figure S7.1: Cumulative Ranking Curve Graph of the Effects of Various Exercise Interventions Incidence of Flow-Mediated Dilation(FMD).The larger the area under the curve , the better the improvement effect on Flow-Mediated Dilation(FMD).

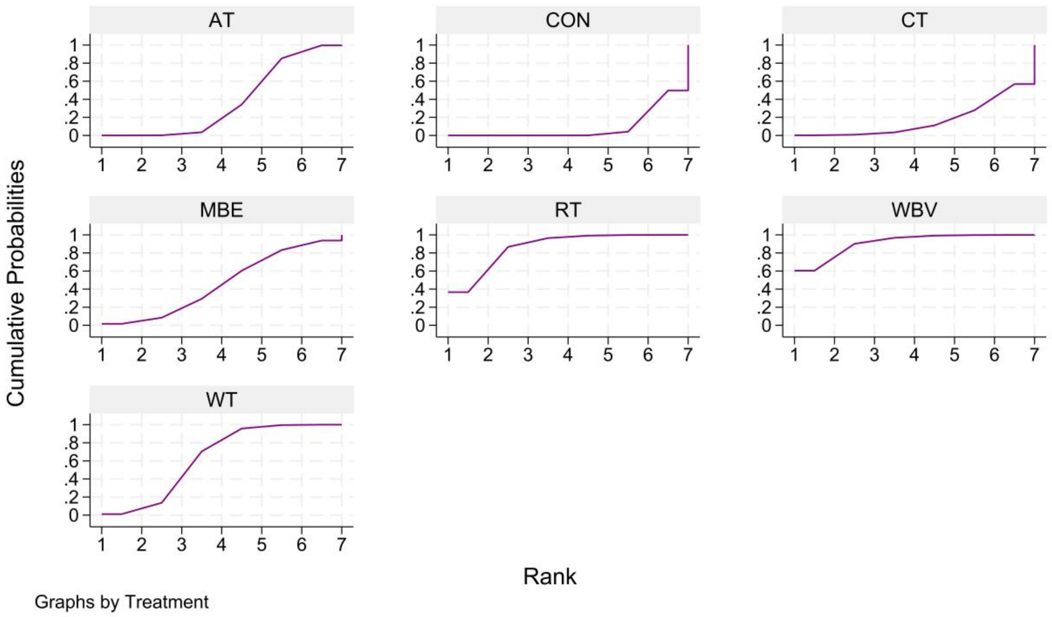

Table S7.1: Assessment of the Effects of Various Exercise Interventions on the Incidence of Flow-Mediated Dilation(FMD)Using SUCRA.

Treatment	SUCRA	PrBest	MeanRank
**MBE**	46.1	1.6	4.2
**RT**	86.5	36.7	1.8
**WBV**	91	60.4	1.5
**CON**	9	0	6.5
**AT**	37.2	0	4.8
**WT**	63.4	1.1	3.2
**CT**	16.7	0.2	6

Abbreviations: SUCRA, surface under the cumulative ranking curve.

Figure S7.2: Cumulative Ranking Curve Graph of the Effects of Various Exercise Interventions Incidence of Carotid-Femoral Pulse Wave Velocity(cfPWV).The larger the area under the curve , the better the improvement effect on Carotid-Femoral Pulse Wave Velocity.

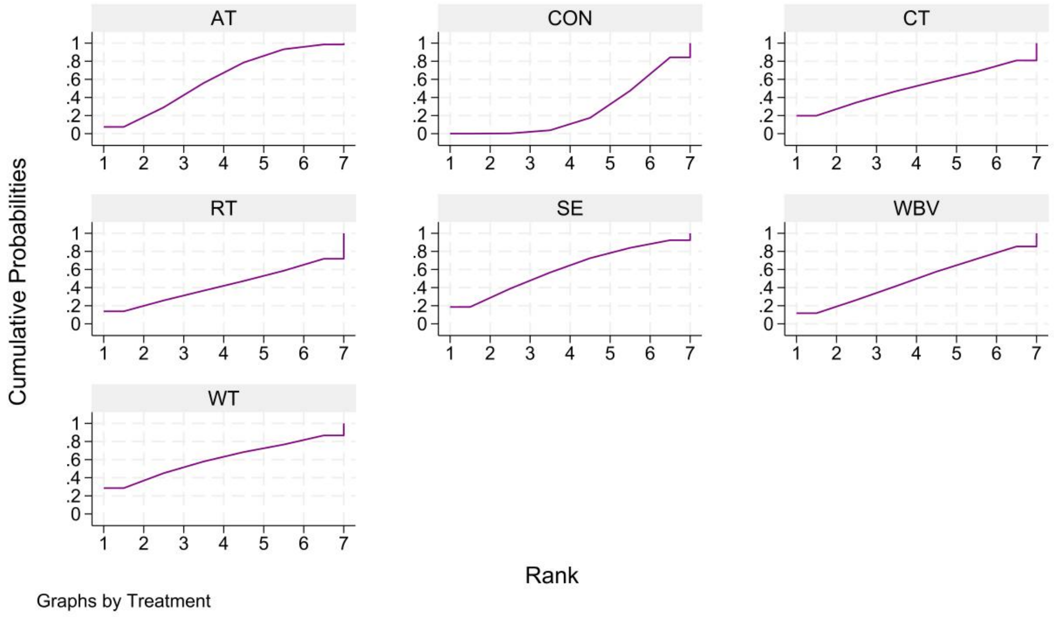

Table S7.2: Assessment of the Effects of Various Exercise Interventions on the Carotid-Femoral Pulse Wave Velocity Using SUCRA.

Treatment	SUCRA	PrBest	MeanRank
SE	60.5	18.6	3.4
RT	42.3	13.8	4.5
WBV	49.1	11.7	4.1
CON	25.6	0	5.5
AT	60.5	7.4	3.4
WT	60.5	28.5	3.4
CT	51.5	19.9	3.9

Abbreviations: SUCRA, surface under the cumulative ranking curve.

Figure S7.3: Cumulative Ranking Curve Graph of the Effects of Various Exercise Interventions Incidence of Brachial-Ankle Pulse Wave Velocity(baPWV).The larger the area under the curve,the better the improvement effect on Brachial-Ankle Pulse Wave Velocity.

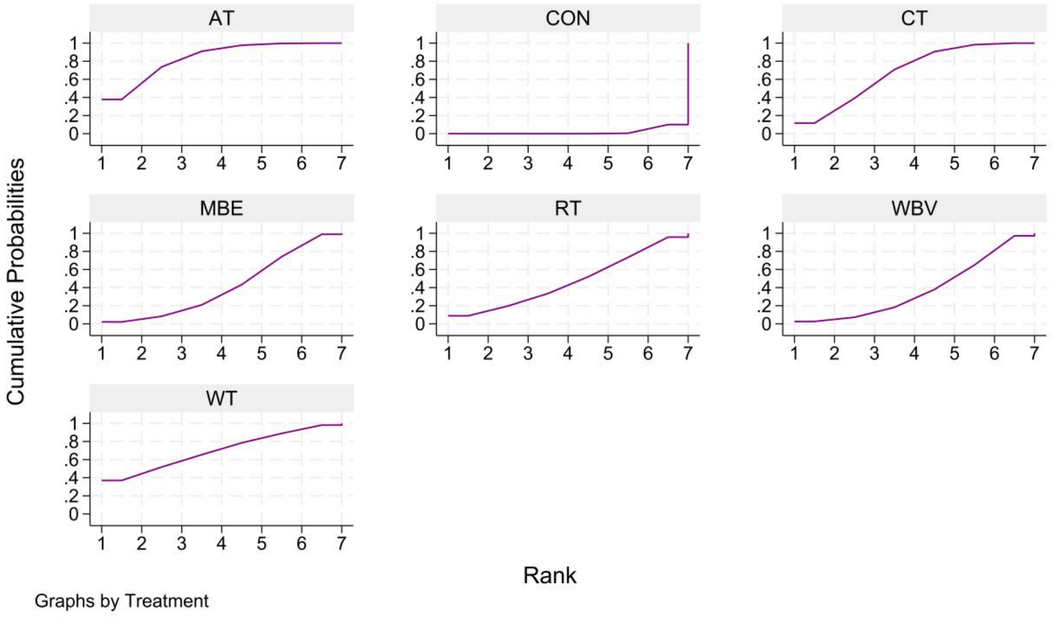

Table S7.3: Assessment of the Effects of Various Exercise Interventions on the Incidence of Brachial-Ankle Pulse Wave Velocity Using SUCRA.

Treatment	SUCRA	PrBest	MeanRank
MBE	41.3	2.1	4.5
RT	47.2	8.9	4.2
WBV	38	2.6	4.7
CON	1.8	0	6.9
AT	83.3	37.8	2
WT	70	37	2.8
CT	68.4	11.6	2.9

Abbreviations: SUCRA, surface under the cumulative ranking curve.

Figure S7.4: Cumulative Ranking Curve Graph of the Effects of Various Exercise Interventions Incidence of Augmentation Index(AIx).The larger the area under the curve , the better the improvement effect on Augmentation Index.

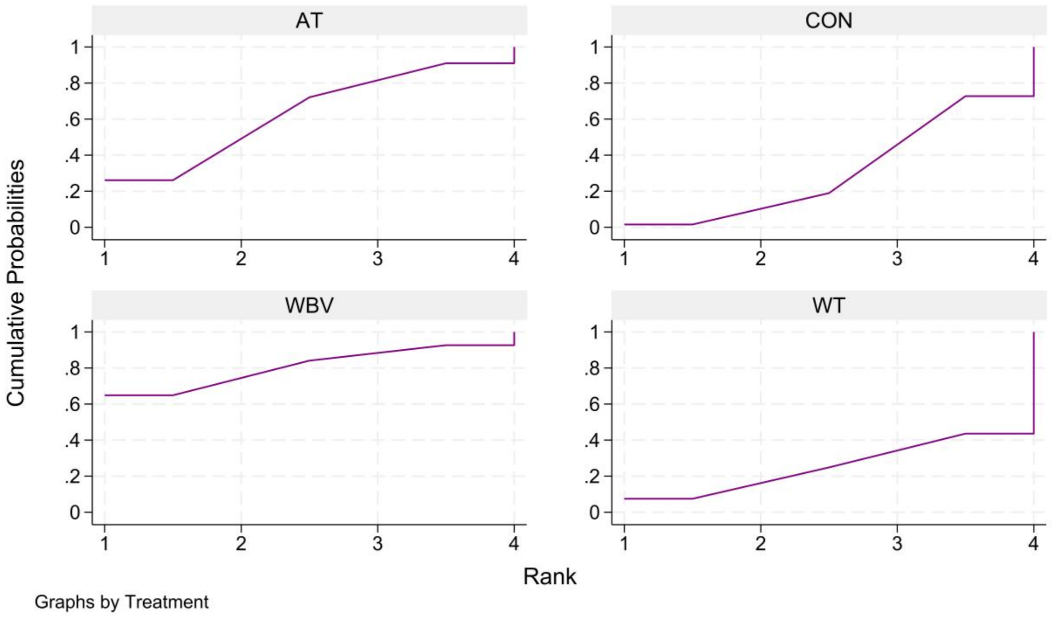

Table S7.4: Assessment of the Effects of Various Exercise Interventions on the Incidence of Augmentation Index Using SUCRA.

Treatment	SUCRA	PrBest	MeanRank
WBV	80.5	64.9	1.6
CON	31.1	1.5	3.1
AT	63.1	26.1	2.1
WT	25.3	7.5	3.2

Abbreviations: SUCRA, surface under the cumulative ranking curve.

Figure S7.5: Cumulative Ranking Curve Graph of the Effects of Various Exercise Interventions Incidence of Heart Rate-Corrected Augmentation Index(AIx@75).The larger the area under the curve , the better the improvement effect on Heart Rate-Corrected Augmentation Index.

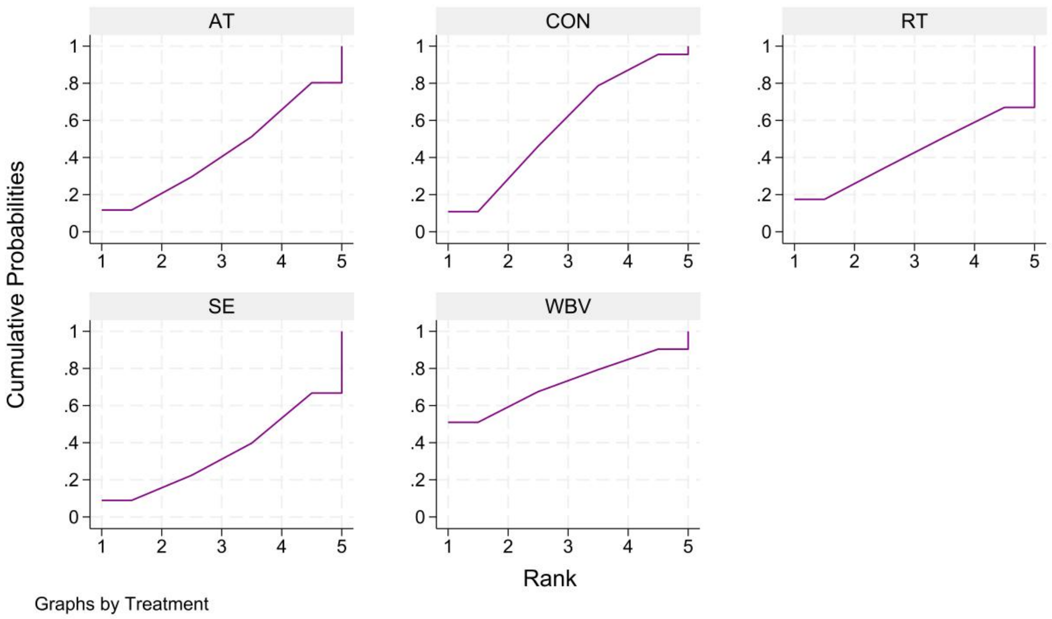

Table S7.5: Assessment of the Effects of Various Exercise Interventions on the Incidence of Heart Rate-Corrected Augmentation Index Using SUCRA.

Treatment	SUCRA	PrBest	MeanRank
SE	35.5	9.6	3.6
RT	41.8	17.5	3.3
WBV	71.8	50.3	2.1
CON	58.2	10.8	2.7
AT	42.6	11.8	3.3

Abbreviations: SUCRA, surface under the cumulative ranking curve.

Figure S7.6: Cumulative Ranking Curve Graph of the Effects of Various Exercise Interventions Incidence of Brachial Systolic Blood Pressure(BSBP).The larger the area under the curve , the better the improvement effect on Brachial Systolic Blood Pressure.

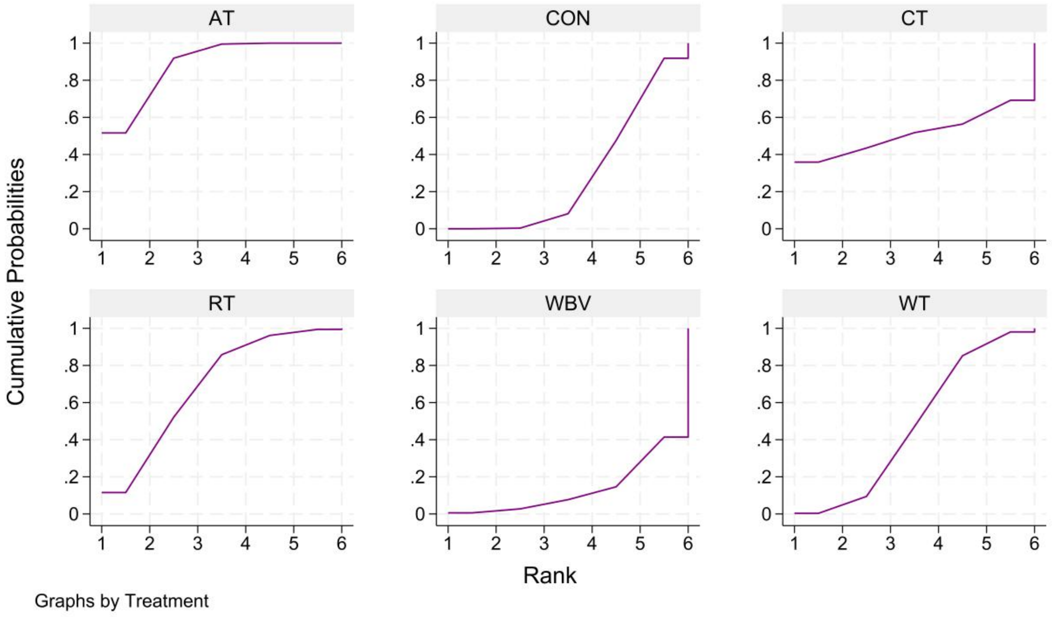

Table S7.6: Assessment of the Effects of Various Exercise Interventions on the Incidence of Brachial Systolic Blood Pressure Using SUCRA.

**Treatment**	**SUCRA**	**PrBest**	**MeanRank**
RT	69	11.5	2.5
WBV	13.4	0.6	5.3
CON	29.6	0	4.5
AT	88.6	51.6	1.6
WT	48	0.3	3.6
CT	51.4	35.9	3.4

Abbreviations: SUCRA, surface under the cumulative ranking curve.

Figure S7.7: Cumulative Ranking Curve Graph of the Effects of Various Exercise Interventions Incidence of Systolic Blood Pressure(SBP).The larger the area under the curve , the better the improvement effect on Systolic Blood Pressure.

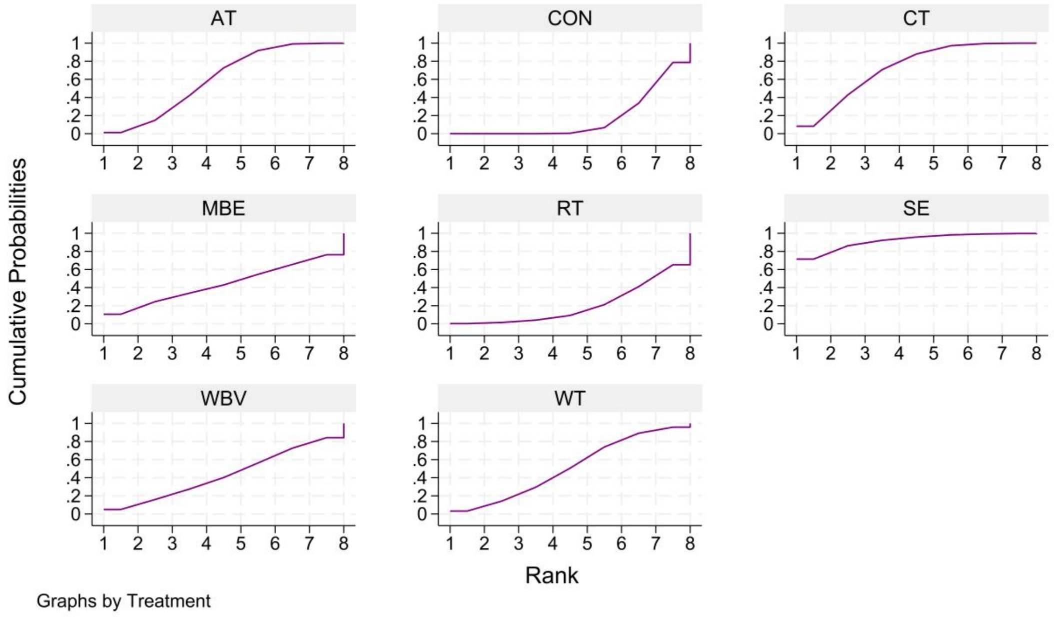

Table S7.7: Assessment of the Effects of Various Exercise Interventions on the Incidence of Systolic Blood Pressure Using SUCRA.

Treatment	SUCRA	PrBest	MeanRank
MBE	44.1	10.6	4.9
SE	91.8	71.5	1.6
RT	20.4	0.3	6.6
WBV	43.1	5	5
CON	17.1	0	6.8
AT	60.2	1.2	3.8
WT	50.9	3.2	4.4
CT	72.4	8.3	2.9

Abbreviations: SUCRA, surface under the cumulative ranking curve.

Figure S7.8: Cumulative Ranking Curve Graph of the Effects of Various Exercise Interventions Incidence of Diastolic Blood Pressure(DBP).The larger the area under the curve , the better the improvement effect on Diastolic Blood Pressure.

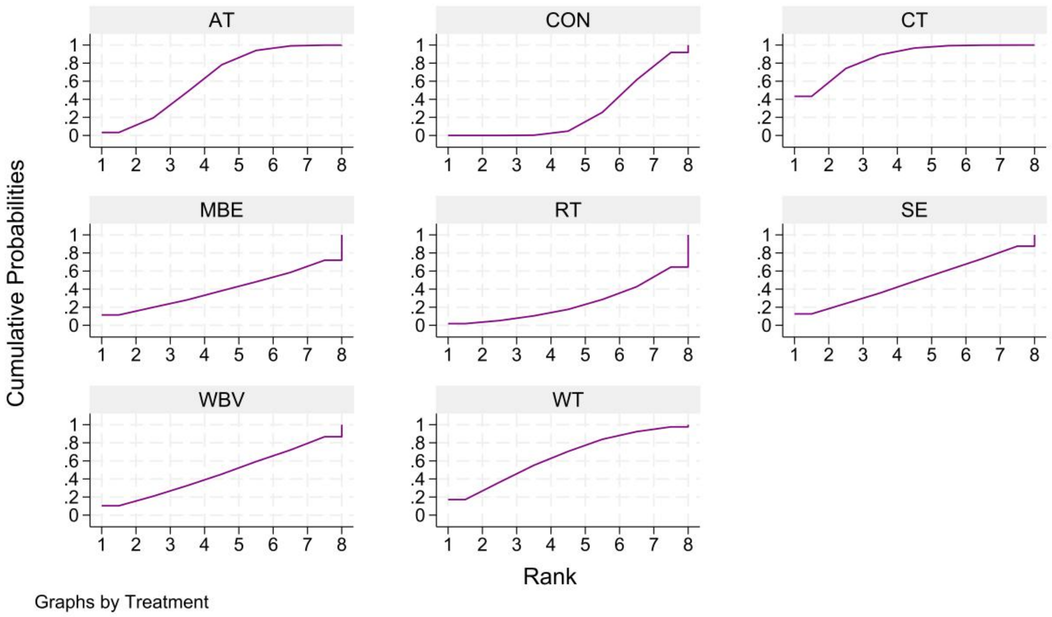

Table S7.8: Assessment of the Effects of Various Exercise Interventions on the Incidence of Diastolic Blood Pressure Using SUCRA.

Treatment	SUCRA	PrBest	MeanRank
MBE	39.5	11.4	5.2
SE	49.2	12.6	4.6
RT	24.4	1.9	6.3
WBV	46.7	10.4	4.7
CON	26.3	0	6.2
AT	63.1	3.3	3.6
WT	64.7	17.2	3.5
CT	86.1	43.2	2

Abbreviations: SUCRA, surface under the cumulative ranking curve.

Figure S7.9: Cumulative Ranking Curve Graph of the Effects of Various Exercise Interventions Incidence of Carotid Beta-Stiffness Index (β) (β -index).The larger the area under the curve , the better the improvement effect on Carotid Beta-Stiffness Index (β) .

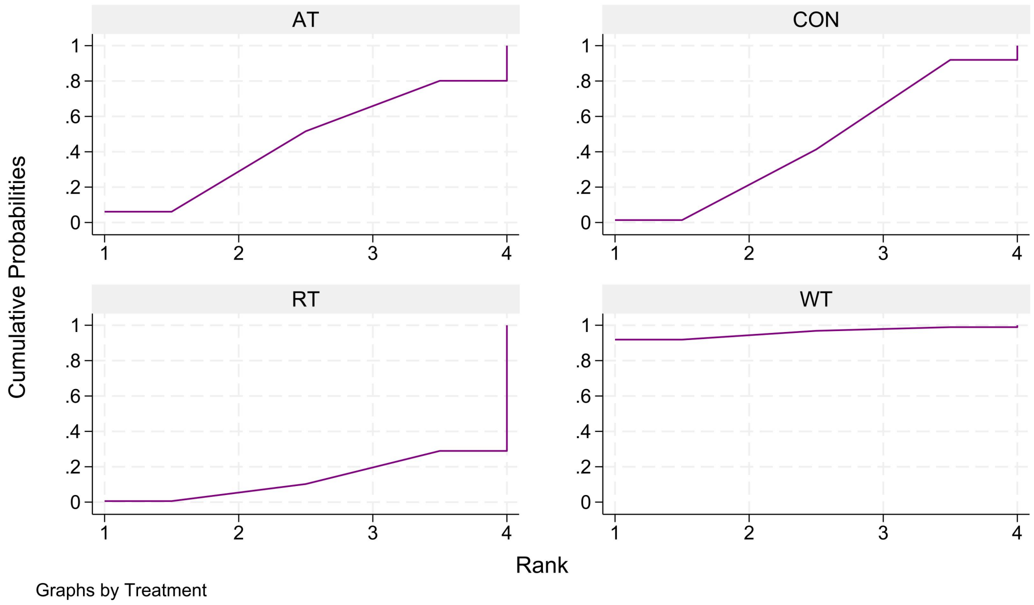

Table S7.9: Assessment of the Effects of Various Exercise Interventions on the Incidence of Carotid Beta-Stiffness Index (β) Using SUCRA.

Treatment	SUCRA	PrBest	MeanRank
RT	13.3	0.6	3.6
CON	44.9	1.4	2.7
AT	46	6.1	2.6
WT	95.9	91.9	1.1

Abbreviations: SUCRA, surface under the cumulative ranking curve.

Figure S7.10: Cumulative Ranking Curve Graph of the Effects of Various Exercise Interventions Incidence of Ankle-Brachial Index(ABI).The larger the area under the curve , the better the improvement effect on Ankle-Brachial Index.

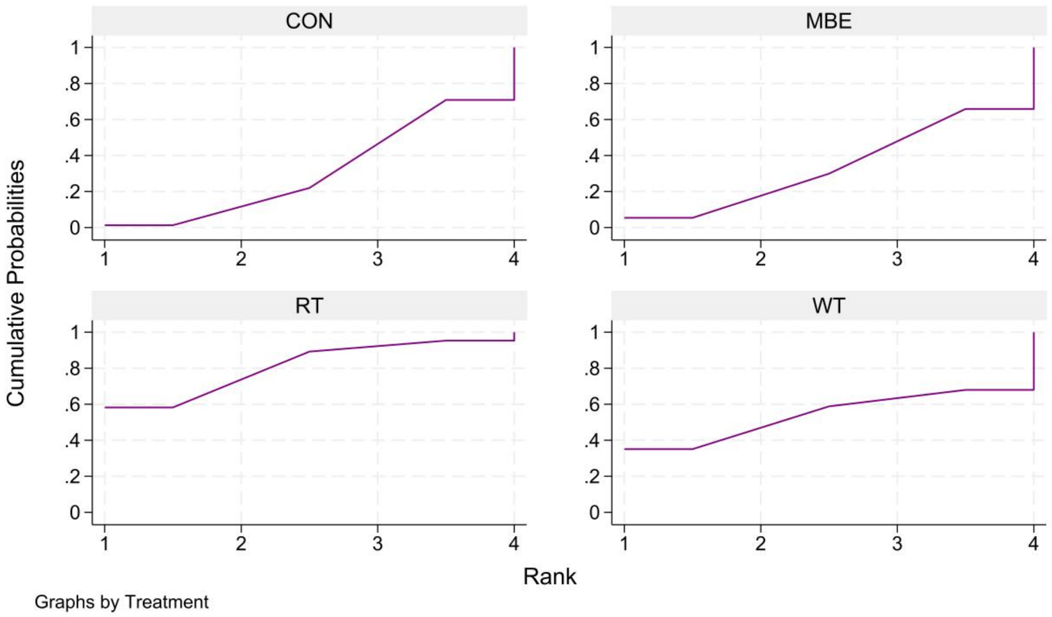

Table S7.10: Assessment of the Effects of Various Exercise Interventions on the Incidence of Ankle-Brachial Index Using SUCRA.

Treatment	SUCRA	PrBest	MeanRank
MBE	33.7	5.4	3
RT	80.9	58.2	1.6
CON	31.4	1.3	3.1
WT	54	35.1	2.4

Abbreviations: SUCRA, surface under the cumulative ranking curve.

Figure S7.11: Cumulative Ranking Curve Graph of the Effects of Various Exercise Interventions Incidence of Femoral-Ankle Pulse Wave Velocity(faPWV).The larger the area under the curve , the better the improvement effect on Femoral-Ankle Pulse Wave Velocity.

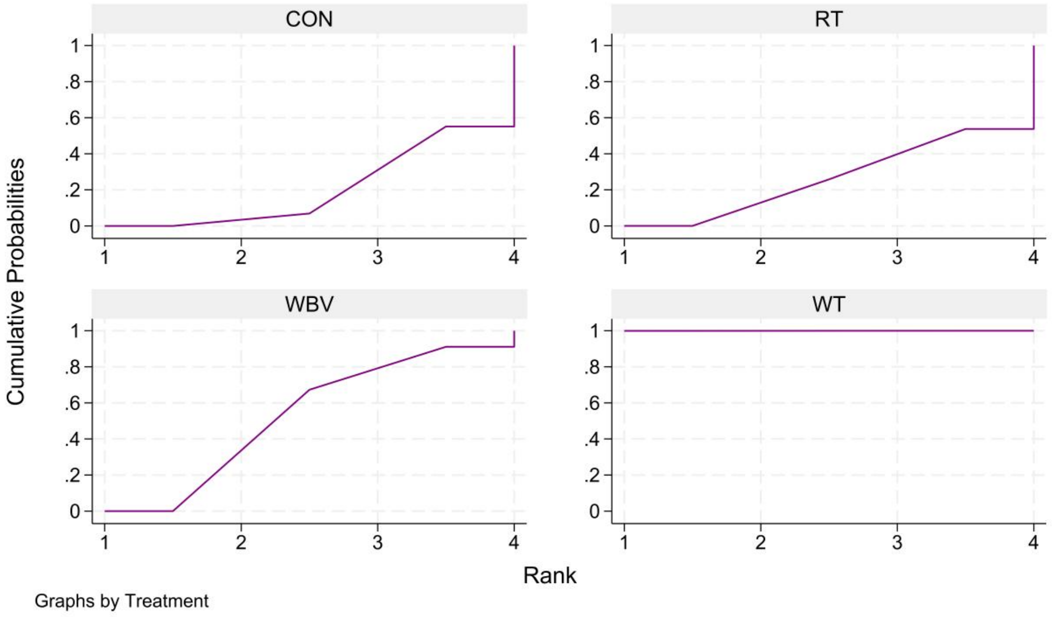

Table S7.11: Assessment of the Effects of Various Exercise Interventions on the Incidence of Femoral-Ankle Pulse Wave Velocity Using SUCRA.

Treatment	SUCRA	PrBest	MeanRank
RT	26.5	0	3.2
WBV	52.8	0	2.4
CON	20.7	0	3.4
WT	100	99.9	1

Abbreviations: SUCRA, surface under the cumulative ranking curve.

Figure S7.12: Cumulative Ranking Curve Graph of the Effects of Various Exercise Interventions Incidence of Body Mass Index(BMI).The larger the area under the curve , the better the improvement effect on Body Mass Index.

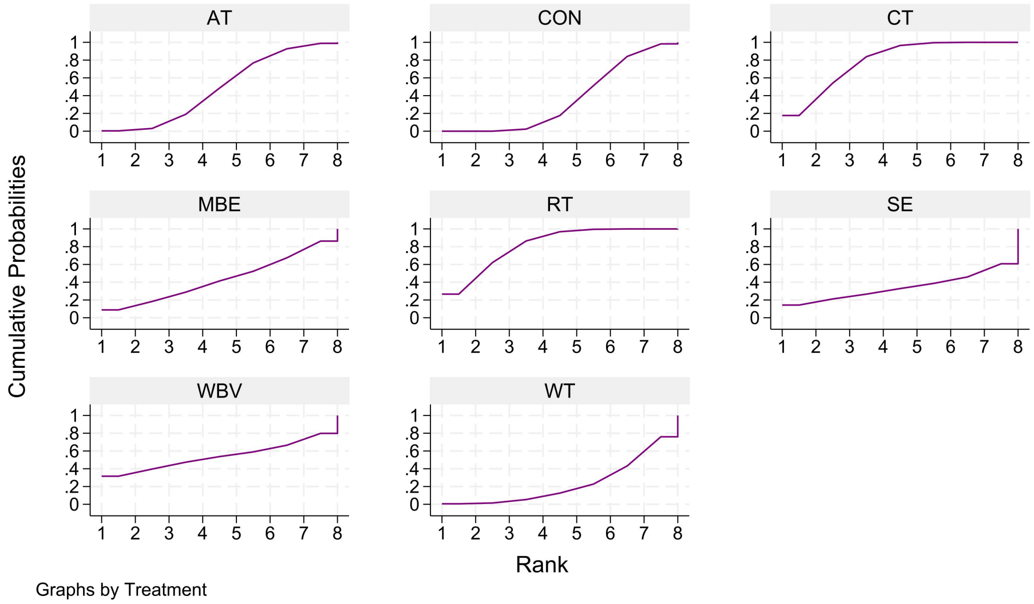

Table S7.12: Assessment of the Effects of Various Exercise Interventions on the Incidence of Body Mass Index.

Treatment	SUCRA	PrBest	MeanRank
MBE	43.4	8.9	5
SE	34.3	14.3	5.6
RT	81.6	26.6	2.3
WBV	54	31.6	4.2
CON	36.2	0	5.5
AT	48.5	0.4	4.6
WT	23.1	0.6	6.4
CT	78.8	17.6	2.5

Abbreviations: SUCRA, surface under the cumulative ranking curve.

Appendix 8: League table of relative effect estimates for different exercise modalities on arterial stiffness and endothelial function: A network meta-analysis of 47 trials.

Table S8.1: Flow-mediated dilation (FMD).

MBE						
-2. 91 (-6. 91, 1. 08)	RT					
-3. 56 (-8. 04, 0. 92)	-0. 65 (-4. 87, 3. 58)	WBV				
2. 14 (-0. 89, 5. 16)	5. 05 (2. 41, 7. 68)	5. 70 (2. 40, 9. 00)	CON			
0. 56 (-2. 70, 3. 82)	3. 47 (0. 53, 6. 42)	4. 12 (0. 59, 7. 66)	-1. 58 (-2. 83, -0. 32)	AT		
-0. 97 (-4. 33, 2. 38)	1. 94 (-1. 07, 4. 94)	2. 59 (-1. 03, 6. 21)	-3. 11 (-4. 59, -1. 64)	-1. 54 (-3. 39, 0. 32)	WT	
1. 91 (-2. 33, 6. 15)	4. 82 (0. 81, 8. 82)	5. 47 (1. 00, 9. 94)	-0. 23 (-3. 24, 2. 78)	1. 35 (-1. 92, 4. 61)	2. 88 (-0. 47, 6. 24)	CT

Table S8.2:cfPWV, Carotid-Femoral Pulse Wave Velocity

SE						
−0. 56 (−3. 88, 2. 76)	RT					
−0. 32 (−3. 03, 2. 39)	0. 24 (−3. 06, 3. 55)	WBV				
−0. 86 (−2. 79, 1. 08)	−0. 30 (−3. 00, 2. 41)	−0. 54 (−2. 44, 1. 36)	CON			
−0. 06 (−1. 99, 1. 87)	0. 50 (−2. 40, 3. 40)	0. 26 (−1. 92, 2. 43)	0. 80 (−0. 26, 1. 86)	AT		
0. 09 (−3. 25, 3. 44)	0. 66 (−3. 19, 4. 50)	0. 41 (−2. 91, 3. 74)	0. 95 (−1. 78, 3. 68)	0. 15 (−2. 77, 3. 08)	WT	
−0. 25 (−3. 56, 3. 05)	0. 31 (−3. 50, 4. 11)	0. 06 (−3. 22, 3. 35)	0. 60 (−2. 07, 3. 28)	−0. 19 (−3. 07, 2. 68)	−0. 35 (−4. 17, 3. 48)	CT

Table S8.3:baPWV, Brachial-Ankle Pulse Wave Velocity

MBE						
0. 04 (−0. 66, 0. 75)	RT					
−0. 04 (−0. 64, 0. 55)	−0. 08 (−0. 81, 0. 64)	WBV				
−0. 46 (−0. 86, −0. 06)	−0. 50 (−1. 08, 0. 08)	−0. 42 (−0. 86, 0. 02)	CON			
0. 35 (−0. 13, 0. 84)	0. 31 (−0. 33, 0. 95)	0. 39 (−0. 12, 0. 91)	0. 81 (0. 54, 1. 09)	AT		
0. 27 (−0. 53, 1. 08)	0. 23 (−0. 67, 1. 14)	0. 31 (−0. 51, 1. 14)	0. 74 (0. 04, 1. 43)	−0. 08 (−0. 83, 0. 67)	WT	
0. 23 (−0. 25, 0. 71)	0. 19 (−0. 45, 0. 83)	0. 27 (−0. 24, 0. 79)	0. 69 (0. 42, 0. 96)	−0. 12 (−0. 50, 0. 26)	−0. 04 (−0. 79, 0. 71)	CT

Table S8.4:AIx, Augmentation Index

WBV			
-5. 49(-15. 33, 4. 34)	CON		
-2. 89(-14. 30, 8. 51)	2. 60 (-3. 18, 8. 38)	AT	
-6. 59(-19. 57, 6. 38)	-1. 10 (-9. 56, 7. 37)	-3. 70(-13. 95, 6. 55)	WT

Table S8.5:AIx@75, Heart Rate-Corrected Augmentation Index

SE				
1.33 (−21.46, 24.12)	RT			
8.12 (−14.49, 30.74)	6.80 (−17.13, 30.72)	WBV		
4.23 (−10.92, 19.38)	2.90 (−14.13, 19.93)	−3.89 (−20.70, 12.91)	CON	
1.58 (−12.27, 15.43)	0.25 (−21.60, 22.10)	−6.54 (−28.21, 15.12)	−2.65 (−16.34, 11.04)	AT

Table S8.6:BSBP, Brachial Systolic Blood Pressure

RT					
−11. 55(−25. 70, 2. 60)	WBV				
−5. 55 (−12. 09, 0. 99)	6. 00 (−6. 55, 18. 55)	CON			
3. 45 (−4. 23, 11. 12)	15. 00 (1. 82, 28. 17)	9. 00 (4. 99, 13. 02)	AT		
−3. 46 (−11. 27, 4. 35)	8. 09 (−5. 16, 21. 35)	2. 09 (−2. 17, 6. 36)	−6. 91(−12. 76, −1. 05)	WT	
−2. 55(−38. 44, 33. 34)	9. 00 (−28. 45, 46. 45)	3. 00 (−32. 29, 38. 29)	−6. 00(−41. 52, 29. 52)	0. 91 (−34. 64, 36. 45)	CT

Table S8.7:SBP, Systolic Blood Pressure

MBE							
7. 74 (−6. 57, 22. 05)	SE						
−2. 79 (−15. 64, 10. 0)	−10. 53 (−20. 38, −0. 6)	RT					
−0. 04 (−14. 08, 14. 0)	−7. 78 (−19. 37, 3. 81)	2. 75 (−7. 00, 12. 49)	WBV				
−2. 69 (−14. 25, 8. 87)	−10. 43 (−18. 88, −1. 9)	0. 09 (−5. 54, 5. 72)	−2. 65 (−10. 62, 5. 31)	CON			
1. 83 (−10. 16, 13. 82)	−5. 91 (−14. 08, 2. 26)	4. 62 (−1. 57, 10. 80)	1. 87 (−6. 71, 10. 45)	4. 53 (1. 34, 7. 71)	AT		
0. 90 (−11. 87, 13. 66)	−6. 85 (−16. 77, 3. 08)	3. 68 (−4. 10, 11. 46)	0. 94 (−8. 70, 10. 57)	3. 59 (−1. 82, 9. 0)	−0. 94 (−6. 89, 5. 02)	WT	
3. 16 (−8. 97, 15. 29)	−4. 58 (−13. 66, 4. 50)	5. 95 (−0. 74, 12. 63)	3. 20 (−5. 58, 11. 98)	5. 85 (2. 16, 9. 55)	1. 33 (−3. 54, 6. 19)	2. 26 (−4. 30, 8. 82)	CT

Table S8.8:DBP, Diastolic Blood Pressure

MBE							
0. 85 (−7. 06, 8. 77)	SE						
−0. 96 (−8. 56, 6. 63)	−1. 81 (−8. 20, 4. 57)	RT					
0. 70 (−7. 00, 8. 39)	−0. 16 (−6. 76, 6. 45)	1. 66 (−4. 55, 7. 87)	WBV				
−0. 31 (−6. 57, 5. 95)	−1. 16 (−6. 01, 3. 68)	0. 65 (−3. 65, 4. 95)	−1. 01 (−5. 49, 3. 47)	CON			
1. 66 (−4. 82, 8. 14)	0. 80 (−3. 92, 5. 53)	2. 62 (−1. 80, 7. 04)	0. 96 (−3. 83, 5. 75)	1. 97 (0. 29, 3. 65)	AT		
1. 85 (−5. 36, 9. 07)	1. 00 (−5. 05, 7. 05)	2. 82 (−2. 79, 8. 42)	1. 16 (−4. 58, 6. 90)	2. 17 (−1. 42, 5. 76)	0. 20 (−3. 78, 4. 17)	WT	
3. 17 (−3. 42, 9. 75)	2. 31 (−2. 94, 7. 56)	4. 13 (−0. 64, 8. 89)	2. 47 (−2. 46, 7. 40)	3. 48 (1. 43, 5. 52)	1. 51 (−1. 13, 4. 15)	1. 31 (−2. 84, 5. 46)	CT

Table S8.9:β-index,Carotid Beta-Stiffness Index (β)

RT			
0. 88 (−0. 80, 2. 55)	CON		
0. 97 (−1. 53, 3. 47)	0. 09 (−1. 77, 1. 95)	AT	
3. 58 (0. 26, 6. 89)	2. 70 (−0. 16, 5. 56)	2. 61 (−0. 80, 6. 02)	WT

Table S8.10: ABI, Ankle-Brachial Index

MBE			
-0. 04 (-0. 10, 0. 02)	RT		
0. 00 (-0. 03, 0. 03)	0. 04 (-0. 01, 0. 09)	CON	
-0. 02 (-0. 12, 0. 08)	0. 02 (-0. 09, 0. 13)	-0. 02 (-0. 11, 0. 08)	WT

Table S8.11:faPWV, Femoral-Ankle Pulse Wave Velocity

RT			
0. 34 (-0. 78, 1. 46)	WBV		
-0. 00 (-0. 93, 0. 93)	-0. 34 (-0. 97, 0. 28)	CON	
1. 97 (0. 85, 3. 09)	1. 63 (0. 75, 2. 51)	1. 97 (1. 35, 2. 60)	WT

Table S8.12: BMI, Body Mass Index

MBE							
−0.48 (−3.80, 2.84)	SE						
0.74 (−0.88, 2.37)	1.23 (−1.76, 4.21)	RT					
0.30 (−3.13, 3.72)	0.78 (−3.46, 5.02)	−0.45 (−3.55, 2.66)	WBV				
−0.00 (−1.54, 1.54)	0.48 (−2.46, 3.42)	−0.75 (−1.27, −0.23)	−0.30 (−3.36, 2.76)	CON			
0.16 (−1.45, 1.77)	0.65 (−2.33, 3.62)	−0.58 (−1.27, 0.10)	−0.13 (−3.23, 2.96)	0.17 (−0.30, 0.63)	AT		
−0.41 (−2.26, 1.44)	0.07 (−3.04, 3.18)	−1.16 (−2.30, −0.01)	−0.71 (−3.93, 2.51)	−0.41 (−1.43, 0.61)	−0.57 (−1.69, 0.54)	WT	
0.68 (−0.92, 2.27)	1.16 (−1.81, 4.13)	−0.07 (−0.74, 0.60)	0.38 (−2.71, 3.46)	0.68 (0.26, 1.09)	0.51 (−0.11, 1.14)	1.09 (−0.01, 2.18)	CT

Appendix 9: CINeMA Assessment

We use the CINeMA framework to evidence certainty, assessing it for each network estimate based on the following criteria:

A: Within study bias: We classified the overall risk of bias for each study as low risk of bias, the risk of bias as moderate when none of the four assessed risk of bias items were rated as high risk, and the risk of bias as high when one or both items were rated as high risk. See Appendix 4 for the bias assessment. The risk of bias for a pairwise comparison of each sport is shown in figure S9.1-9.6.

Figure S9.1: Risk of bias contribution by intervention group in FMD.

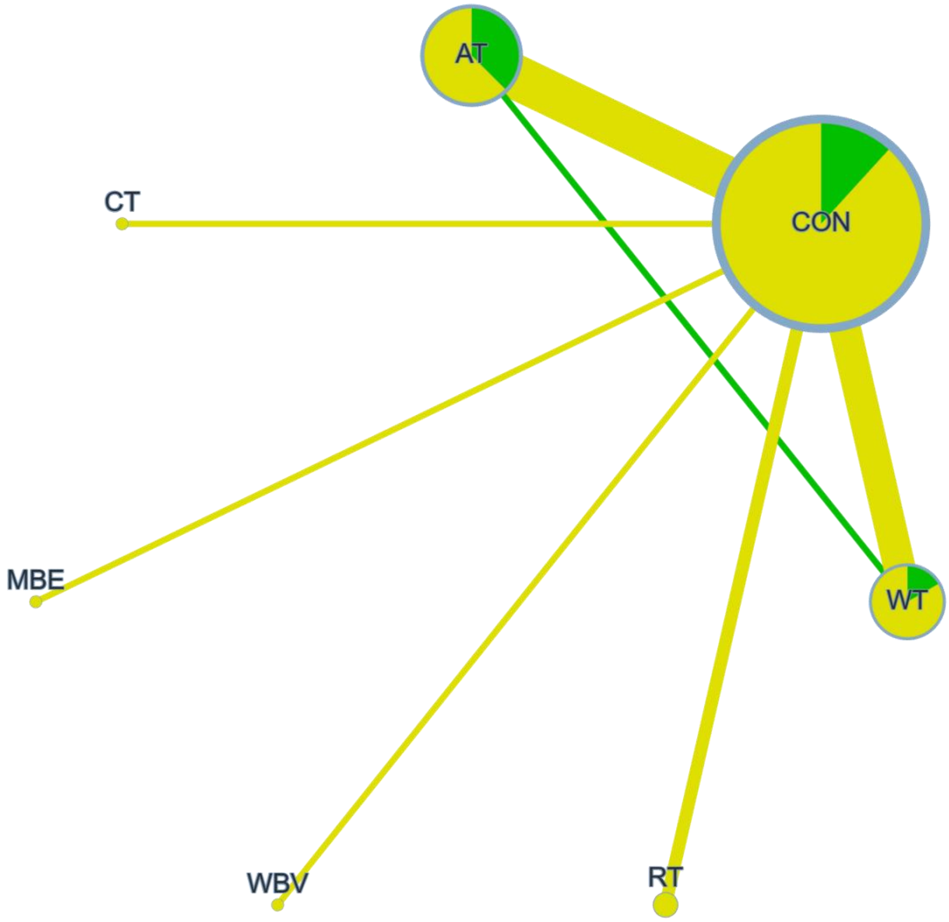

Figure S9.2: Overall risk of bias by treatment comparison in FMD

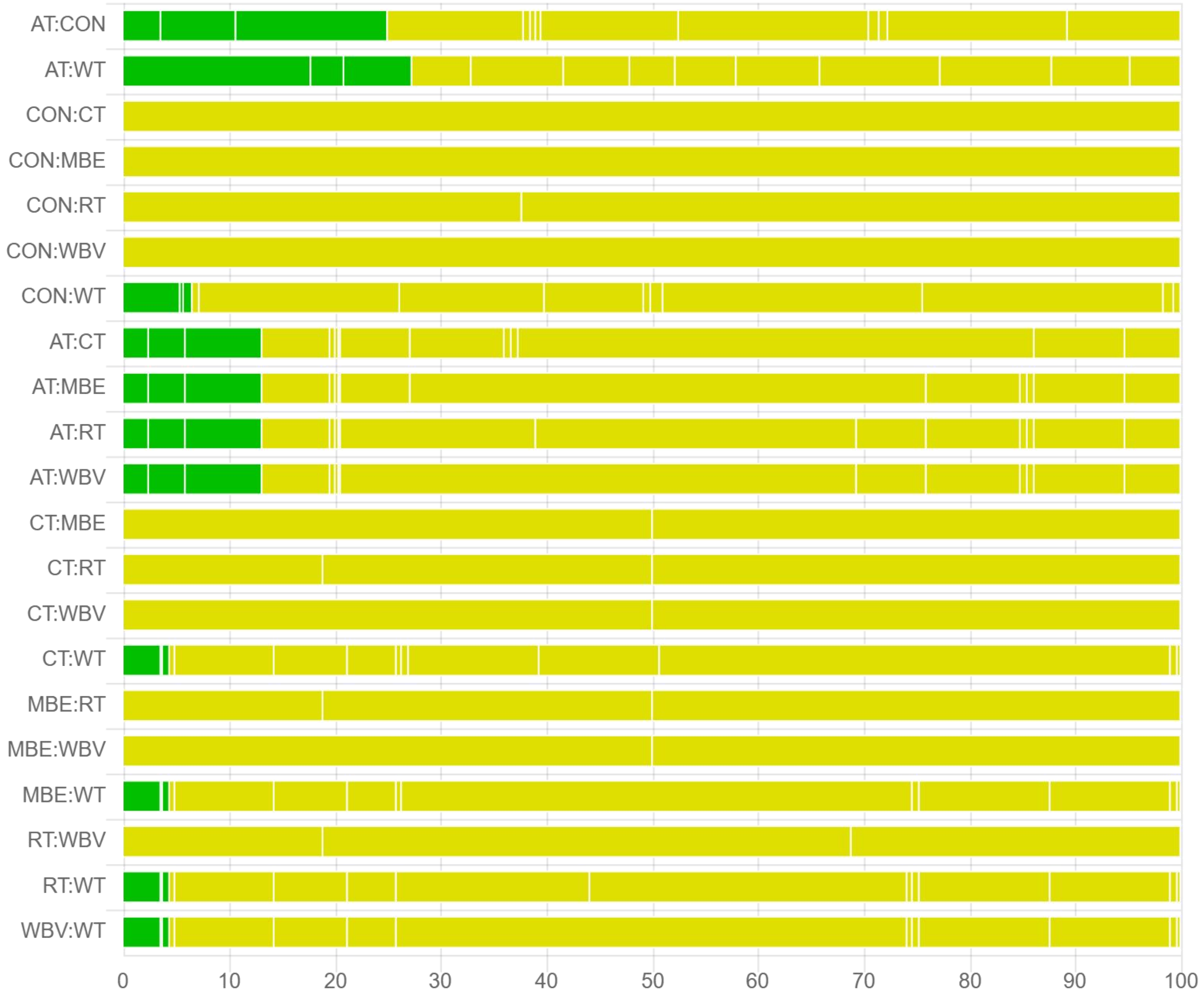

Figure S9.3: Risk of bias contribution by intervention group in cfPWV.

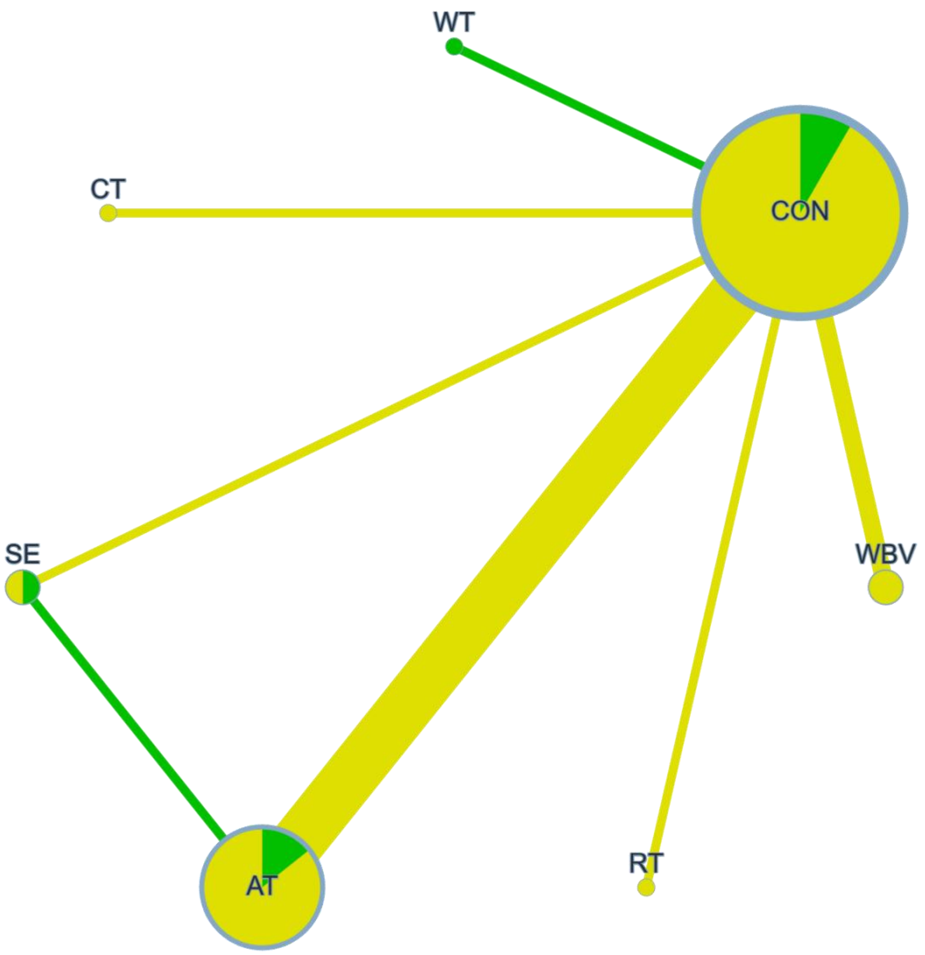

Figure S9.4: Overall risk of bias by treatment comparison in cfPWV

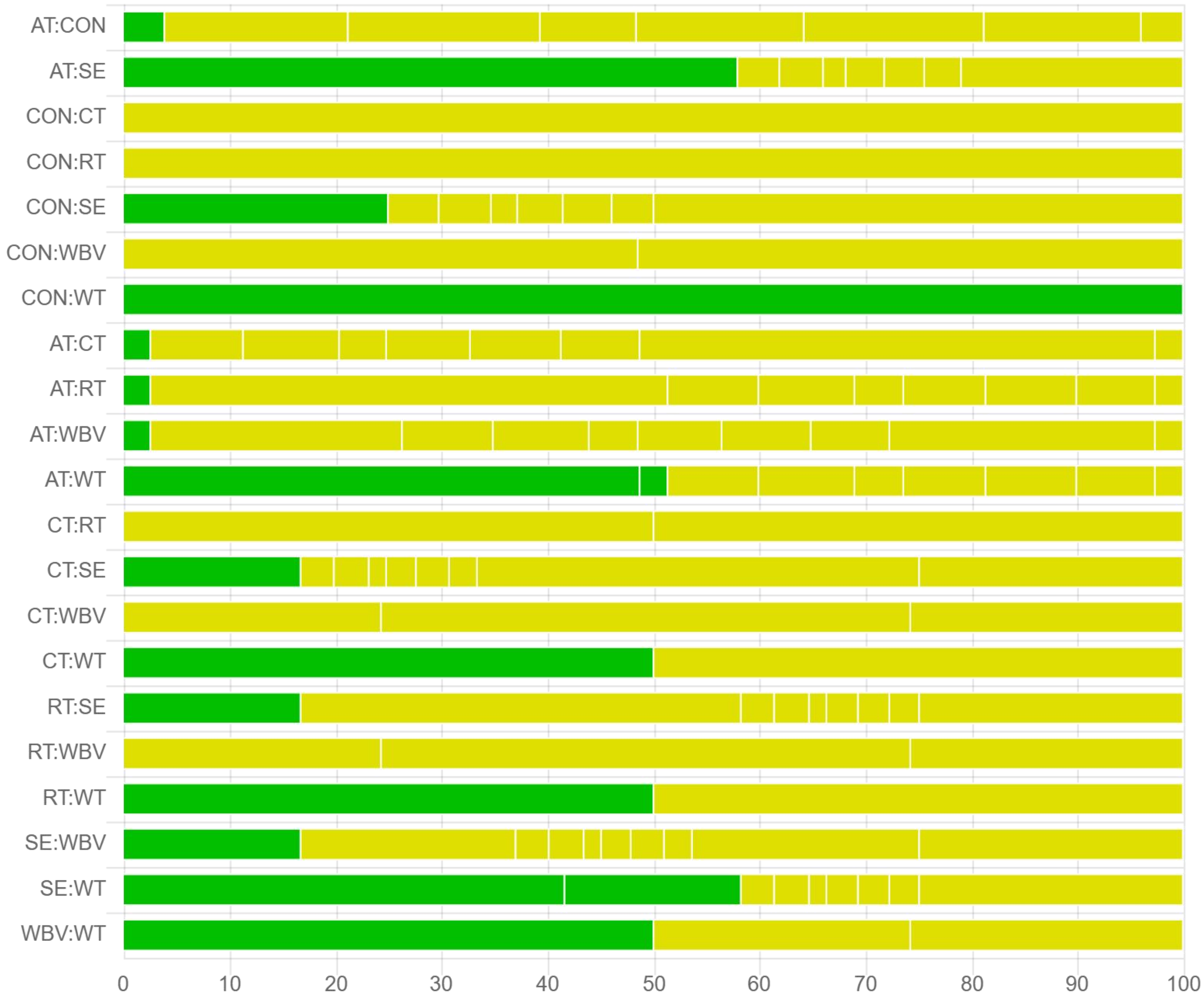

Figure S9.5: Risk of bias contribution by intervention group in baPWV

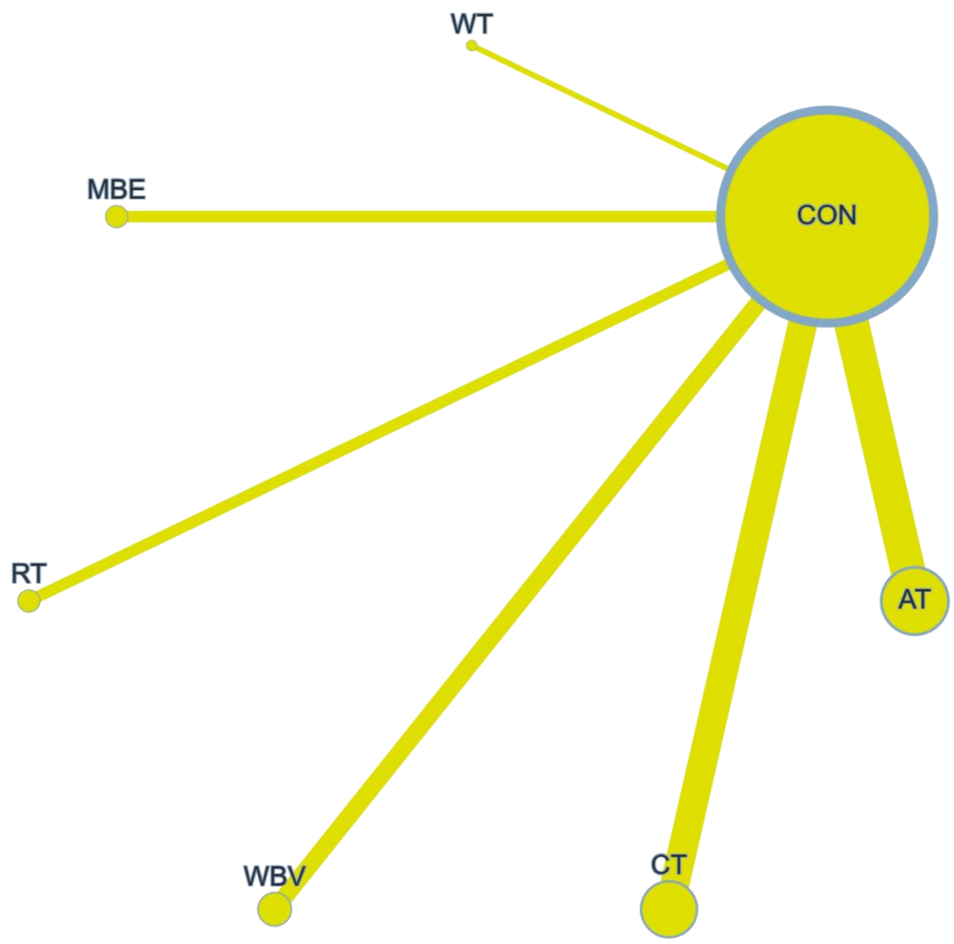

Figure S9.6: Overall risk of bias by treatment comparison in baPWV

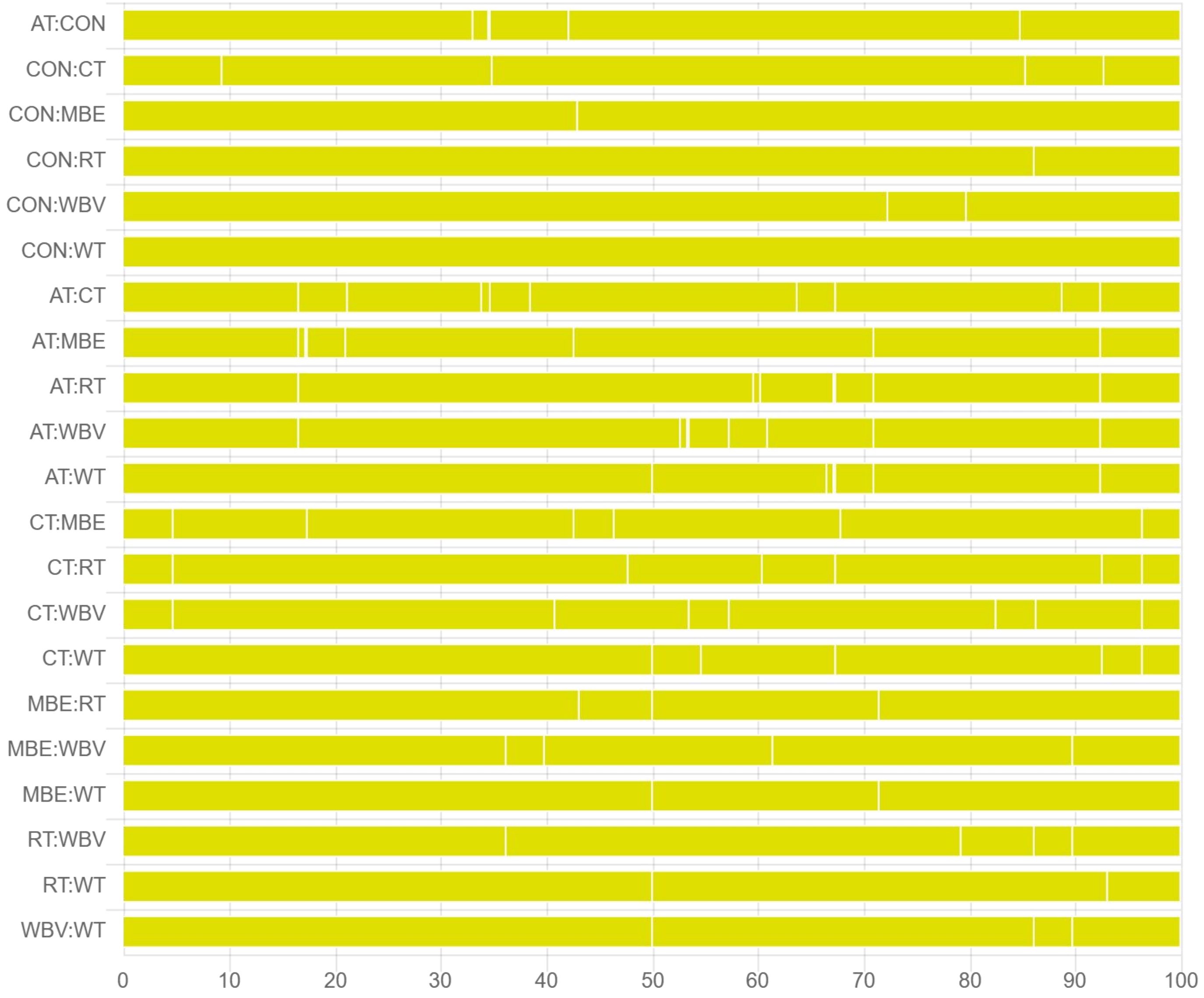

Figure S9.7: Overall risk of bias by treatment comparison in SBP.

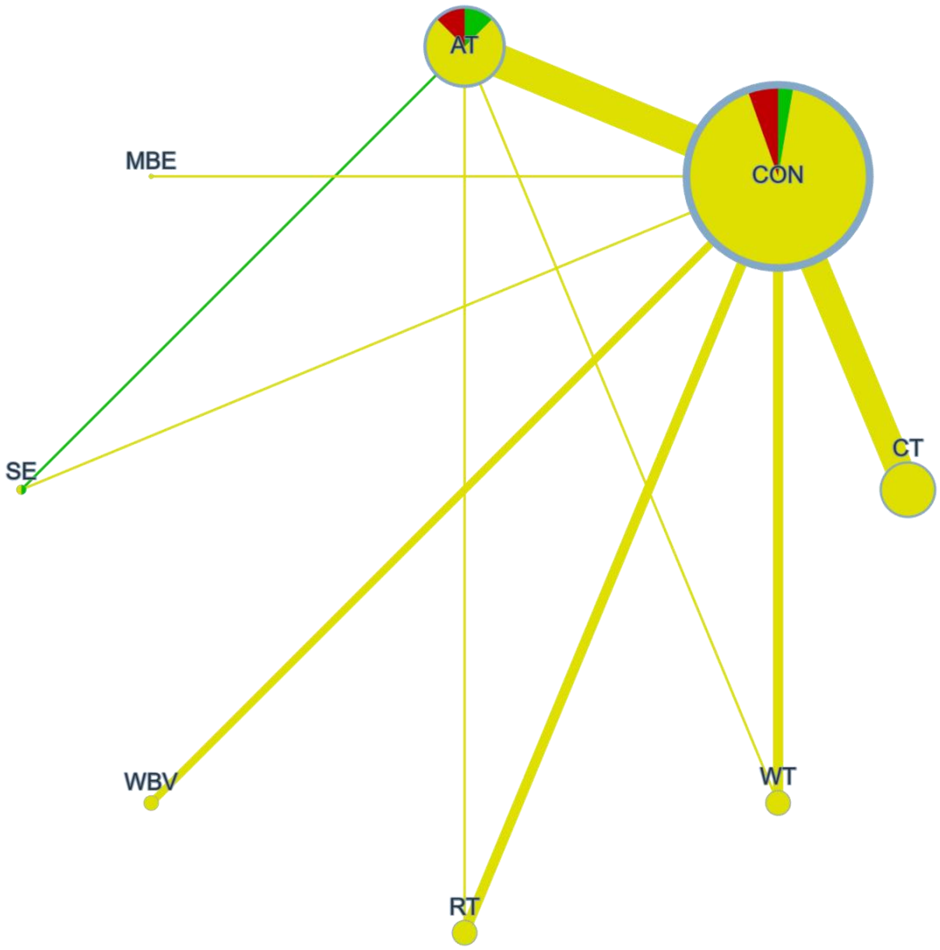

Figure S9.8: Overall risk of bias by treatment comparison in SBP

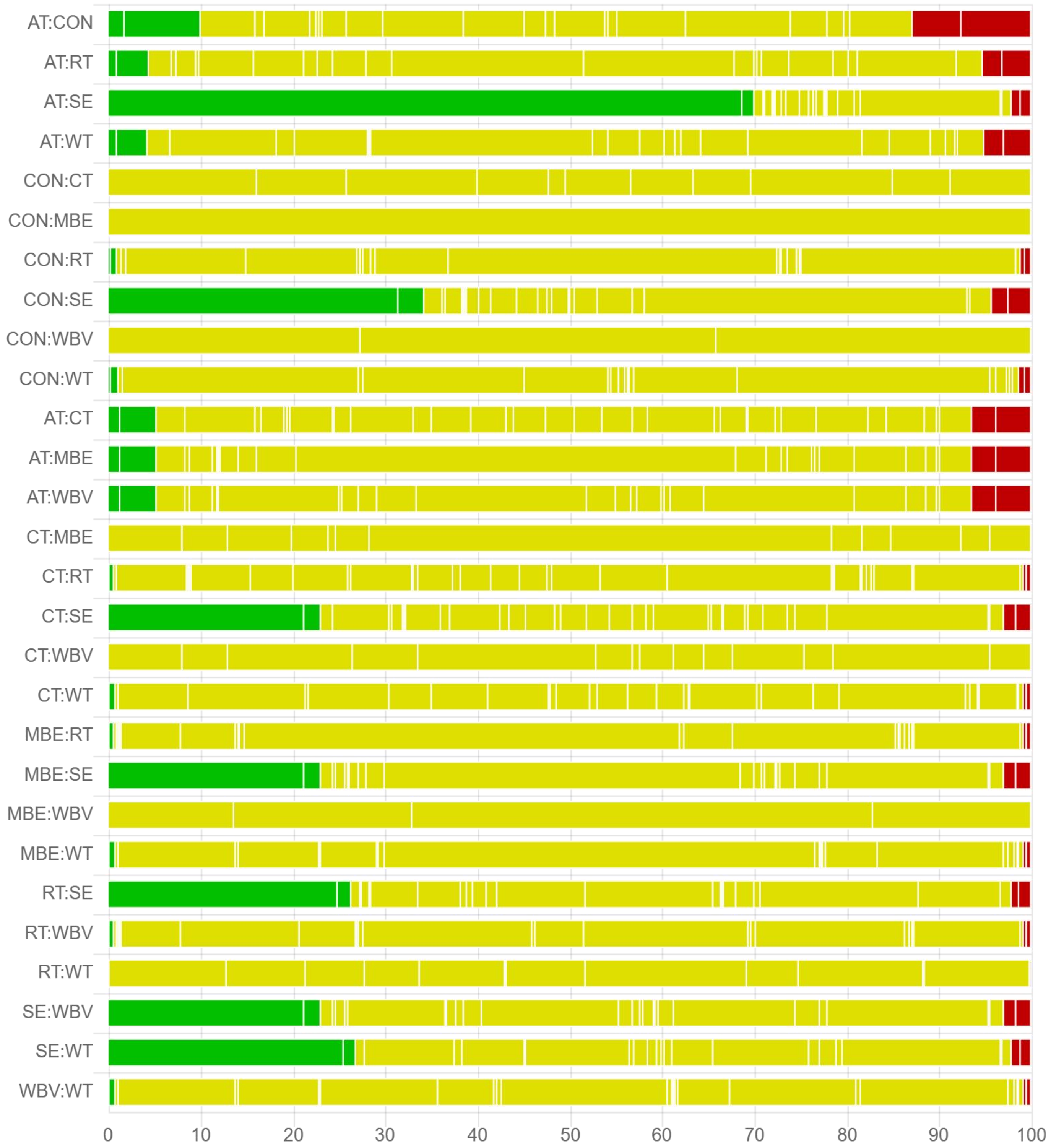

Figure S9.9: Overall risk of bias by treatment comparison in DBP.

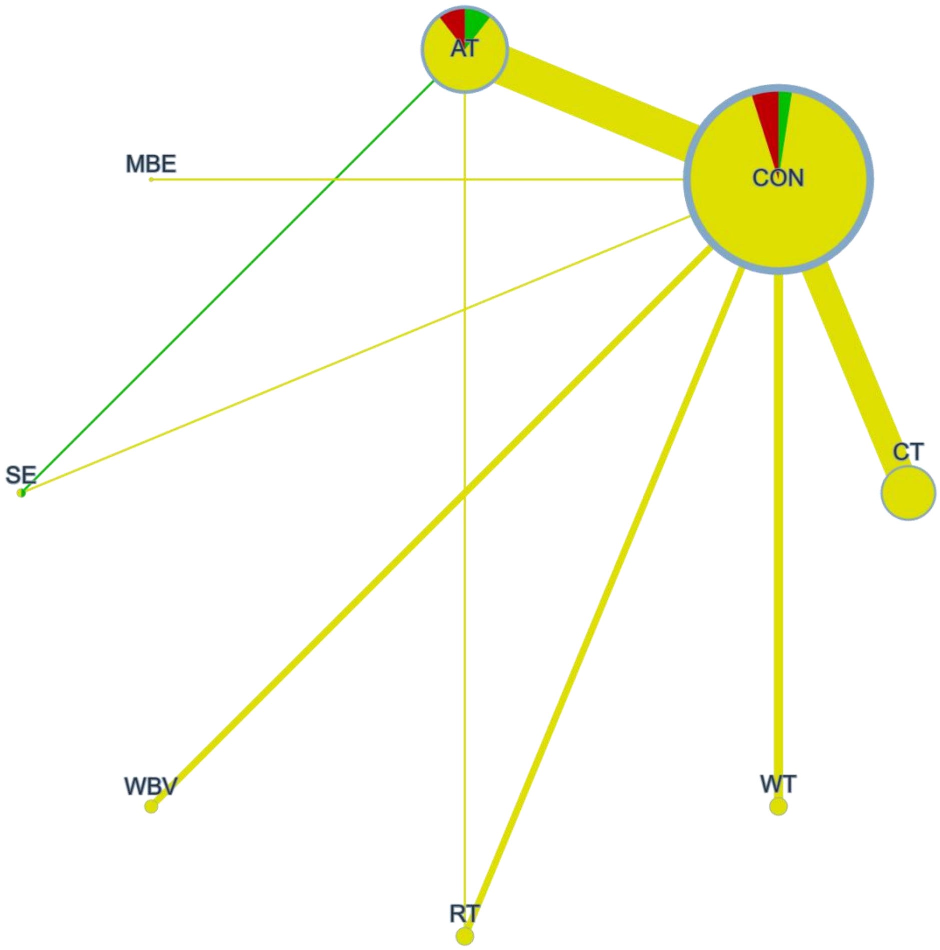

Figure S9.10: Overall risk of bias by treatment comparison in DBP

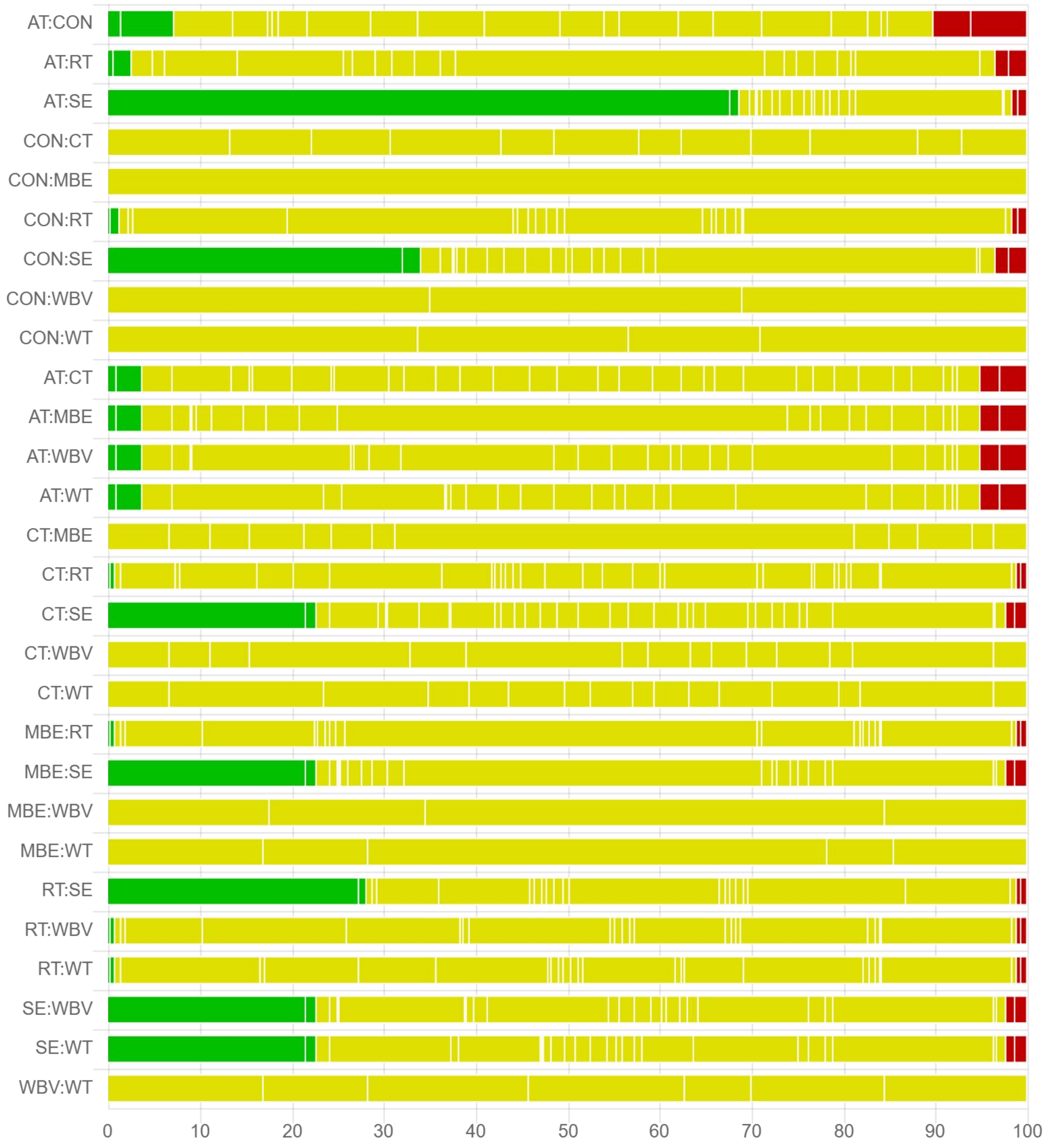

Table S9.1: Transitivity (Indirectness) Assessment

	Baseline variable (Mean ± SD)				
Intervention	Age(year)	BMI	FMD	baPWV	cfPWV
MBE	64.36±5.39	22.57±2.87	5.73±1.88	17.09±2.76	10.34±1.22
SE	63.25±4.32	26.7±3.22	5.6±1.7	17.56± 4.38	10.52±1.70
RT	67.09±3.67	23.67±2.38	6.0±3.25	15.03±1.83	10.59±2.10
WBV	60.73±3.82	26.77±3.13	5.54±1.22	14.88±2.20	11.25±1.25
CON	64.57±5.48	24.88±3.12	5.80±2.29	15.23±1.95	9.20±1.61
AT	65.02±5.17	26.92±2.89	5.02±1.59	14.39±1.39	10.11±1.84
WT	66.07±7.11	26.17±4.88	5.35±2.08	15.53±2.44	11.39± 2.08
CT	68.75±5.79	24.30±2.54	5.11±1.5	15.39±1.40	9.66±1.27

Imprecision: We use the CINeMA website to grade the accuracy of each comparison.

Heterogeneity: We assessed the degree of worry by comparing clinical reasoning based on 95% confidence intervals (CIs) while applying the same clinical

reasoning framework as for inaccuracy. In particular, we judged the consistency of our findings based on the confidence and prediction intervals associated with clinically important effect sizes. And we used the same thresholds of clinical significance as described above and followed the recommendations automatically provided by CINeMA (https://cinema.ispm.unibe.ch/).

Inconsistency: For inconsistency, we looked at the results for node splitting (Appendix 5) and we saw major problems when p<0.10, but otherwise no problems.

Table S9.2: CINeMA Results of FMD

Comparison	Within-study bias	Reporting bias	Indirectness	Imprecision	Heterogeneity	Incoherence	Confidence rating
AT:CON	Some concerns	Low risk	No concerns	No concerns	Major concerns	No concerns	Low
AT:WT	Some concerns	Low risk	No concerns	No concerns	Major concerns	Major concerns	Very low
CON:CT	Some concerns	Low risk	No concerns	Major concerns	No concerns	Some concerns	Low
CON:MBE	Some concerns	Low risk	No concerns	Major concerns	No concerns	Some concerns	Low
CON:RT	Some concerns	Low risk	No concerns	No concerns	No concerns	Some concerns	Moderate
CON:WBV	Some concerns	Low risk	No concerns	No concerns	No concerns	Some concerns	Moderate
CON:WT	Some concerns	Low risk	No concerns	No concerns	Major concerns	Major concerns	Very low
AT:CT	Some concerns	Low risk	No concerns	Major concerns	No concerns	Some concerns	Low
AT:MBE	Some concerns	Low risk	No concerns	Major concerns	No concerns	Some concerns	Low
AT:RT	Some concerns	Low risk	No concerns	No concerns	Major concerns	Some concerns	Low
AT:WBV	Some concerns	Low risk	No concerns	No concerns	Major concerns	Some concerns	Low

CT:MBE	Some concerns	Low risk	No concerns	Major concerns	No concerns	Some concerns	Low
CT:RT	Some concerns	Low risk	No concerns	No concerns	No concerns	Some concerns	Moderate
CT:WBV	Some concerns	Low risk	No concerns	No concerns	No concerns	Some concerns	Moderate
CT:WT	Some concerns	Low risk	No concerns	No concerns	Major concerns	Some concerns	Low
MBE:RT	Some concerns	Low risk	No concerns	Major concerns	No concerns	Some concerns	Low
MBE:WBV	Some concerns	Low risk	No concerns	Major concerns	No concerns	Some concerns	Low
MBE:WT	Some concerns	Low risk	No concerns	Major concerns	No concerns	Some concerns	Low
RT:WBV	Some concerns	Low risk	No concerns	Major concerns	No concerns	Some concerns	Low
RT:WT	Some concerns	Low risk	No concerns	Major concerns	No concerns	Some concerns	Low
WBV:WT	Some concerns	Low risk	No concerns	Major concerns	No concerns	Some concerns	Low

Table S9.3:CINeMA Results of cfPWV

Comparison	Within-study bias	Reporting bias	Indirectness	Imprecision	Heterogeneity	Incoherence	Confidence rating
AT:CON	Some concerns	Low risk	No concerns	No concerns	Major concerns	No concerns	Low
AT:SE	No concerns	Low risk	No concerns	Major concerns	No concerns	No concerns	Low
CON:CT	Some concerns	Low risk	No concerns	Major concerns	No concerns	No concerns	Low
CON:RT	Some concerns	Low risk	No concerns	Major concerns	No concerns	No concerns	Low
CON:SE	Some concerns	Low risk	No concerns	Major concerns	No concerns	No concerns	Low
CON:WBV	Some concerns	Low risk	No concerns	Major concerns	No concerns	No concerns	Low
CON:WT	No concerns	Low risk	No concerns	Major concerns	No concerns	No concerns	Low
AT:CT	Some concerns	Low risk	No concerns	Major concerns	No concerns	No concerns	Low
AT:RT	Some concerns	Low risk	No concerns	Major concerns	No concerns	No concerns	Low
AT:WBV	Some concerns	Low risk	No concerns	Major concerns	No concerns	No concerns	Low
AT:WT	No concerns	Low risk	No concerns	Major concerns	No concerns	No concerns	Low
CT:RT	Some concerns	Low risk	No concerns	Major concerns	No concerns	No concerns	Low
CT:SE	Some concerns	Low risk	No concerns	Major concerns	No concerns	No concerns	Low
CT:WBV	Some concerns	Low risk	No concerns	Major concerns	No concerns	No concerns	Low
CT:WT	No concerns	Low risk	No concerns	Major concerns	No concerns	No concerns	Low
RT:SE	Some concerns	Low risk	No concerns	Major concerns	No concerns	No concerns	Low
RT:WBV	Some concerns	Low risk	No concerns	Major concerns	No concerns	No concerns	Low
RT:WT	No concerns	Low risk	No concerns	Major concerns	No concerns	No concerns	Low
SE:WBV	Some concerns	Low risk	No concerns	Major concerns	No concerns	No concerns	Low
SE:WT	No concerns	Low risk	No concerns	Major concerns	No concerns	No concerns	Low
WBV:WT	No concerns	Low risk	No concerns	Major concerns	No concerns	No concerns	Low

Table S9.4:CINeMA Results of baPWV

Comparison	Within-study bias	Reporting bias	Indirectness	Imprecision	Heterogeneity	Incoherence	Confidence rating
AT:CON	Some concerns	Low risk	No concerns	No concerns	No concerns	Major concerns	Low
CON:CT	Some concerns	Low risk	No concerns	No concerns	No concerns	Major concerns	Low
CON:MBE	Some concerns	Low risk	No concerns	No concerns	No concerns	Major concerns	Low
CON:RT	Some concerns	Low risk	No concerns	Major concerns	No concerns	Major concerns	Very low
CON:WBV	Some concerns	Low risk	No concerns	Major concerns	No concerns	Major concerns	Very low
CON:WT	Some concerns	Low risk	No concerns	No concerns	Major concerns	Major concerns	Very low
AT:CT	Some concerns	Low risk	No concerns	Major concerns	No concerns	Major concerns	Very low
AT:MBE	Some concerns	Low risk	No concerns	Major concerns	No concerns	Major concerns	Very low
AT:RT	Some concerns	Low risk	No concerns	Major concerns	No concerns	Major concerns	Very low
AT:WBV	Some concerns	Low risk	No concerns	Major concerns	No concerns	Major concerns	Very low
AT:WT	Some concerns	Low risk	No concerns	Major concerns	No concerns	Major concerns	Very low
CT:MBE	Some concerns	Low risk	No concerns	Major concerns	No concerns	Major concerns	Very low
CT:RT	Some concerns	Low risk	No concerns	Major concerns	No concerns	Major concerns	Very low
CT:WBV	Some concerns	Low risk	No concerns	Major concerns	No concerns	Major concerns	Very low
CT:WT	Some concerns	Low risk	No concerns	Major concerns	No concerns	Major concerns	Very low
MBE:RT	Some concerns	Low risk	No concerns	Major concerns	No concerns	Major concerns	Very low
MBE:WBV	Some concerns	Low risk	No concerns	Major concerns	No concerns	Major concerns	Very low
MBE:WT	Some concerns	Low risk	No concerns	Major concerns	No concerns	Major concerns	Very low
RT:WBV	Some concerns	Low risk	No concerns	Major concerns	No concerns	Major concerns	Very low
RT:WT	Some concerns	Low risk	No concerns	Major concerns	No concerns	Major concerns	Very low
WBV:WT	Some concerns	Low risk	No concerns	Major concerns	No concerns	Major concerns	Very low

Table S9.5:CINeMA Results of SBP

Comparison	Within-study bias	Reporting bias	Indirectness	Imprecision	Heterogeneity	Incoherence	Confidence rating
AT:CON	Some concerns	Low risk	No concerns	No concerns	Major concerns	No concerns	Low
AT:RT	Some concerns	Low risk	No concerns	Major concerns	No concerns	No concerns	Low
AT:SE	No concerns	Low risk	No concerns	Major concerns	No concerns	Major concerns	Low
AT:WT	Some concerns	Low risk	No concerns	Major concerns	No concerns	No concerns	Low
CON:CT	Some concerns	Low risk	No concerns	No concerns	Major concerns	Major concerns	Very low
CON:MBE	Some concerns	Low risk	No concerns	Major concerns	No concerns	Major concerns	Very low
CON:RT	Some concerns	Low risk	No concerns	Major concerns	No concerns	No concerns	Low
CON:SE	Some concerns	Low risk	No concerns	No concerns	Major concerns	Major concerns	Very low
CON:WBV	Some concerns	Low risk	No concerns	Major concerns	No concerns	Major concerns	Very low
CON:WT	Some concerns	Low risk	No concerns	Major concerns	No concerns	No concerns	Low
AT:CT	Some concerns	Low risk	No concerns	Major concerns	No concerns	Major concerns	Very low
AT:MBE	Some concerns	Low risk	No concerns	Major concerns	No concerns	Major concerns	Very low
AT:WBV	Some concerns	Low risk	No concerns	Major concerns	No concerns	Major concerns	Very low
CT:MBE	Some concerns	Low risk	No concerns	Major concerns	No concerns	Major concerns	Very low
CT:RT	Some concerns	Low risk	No concerns	Major concerns	No concerns	Major concerns	Very low
CT:SE	Some concerns	Low risk	No concerns	Major concerns	No concerns	Major concerns	Very low
CT:WBV	Some concerns	Low risk	No concerns	Major concerns	No concerns	Major concerns	Very low
CT:WT	Some concerns	Low risk	No concerns	Major concerns	No concerns	Major concerns	Very low
MBE:RT	Some concerns	Low risk	No concerns	Major concerns	No concerns	Major concerns	Very low
MBE:SE	Some concerns	Low risk	No concerns	Major concerns	No concerns	Major concerns	Very low
MBE:WBV	Some concerns	Low risk	No concerns	Major concerns	No concerns	Major concerns	Very low
MBE:WT	Some concerns	Low risk	No concerns	Major concerns	No concerns	Major concerns	Very low
RT:SE	Some concerns	Low risk	No concerns	No concerns	Major concerns	Major concerns	Very low
RT:WBV	Some concerns	Low risk	No concerns	Major concerns	No concerns	Major concerns	Very low
RT:WT	Some concerns	Low risk	No concerns	Major concerns	No concerns	Major concerns	Very low
SE:WBV	Some concerns	Low risk	No concerns	Major concerns	No concerns	Major concerns	Very low

SE:WT	Some concerns	Low risk	No concerns	Major concerns	No concerns	Major concerns	Very low
WBV:WT	Some concerns	Low risk	No concerns	Major concerns	No concerns	Major concerns	Very low

Table S9.6:CINeMA Results of DBP

Comparison	Within-study bias	Reporting bias	Indirectness	Imprecision	Heterogeneity	Incoherence	Confidence rating
AT:CON	Some concerns	Low risk	No concerns	No concerns	Major concerns	No concerns	Low
AT:RT	Some concerns	Low risk	No concerns	Major concerns	No concerns	No concerns	Low
AT:SE	No concerns	Low risk	No concerns	Major concerns	No concerns	No concerns	Low
CON:CT	Some concerns	Low risk	No concerns	No concerns	Major concerns	No concerns	Low
CON:MBE	Some concerns	Low risk	No concerns	Major concerns	No concerns	No concerns	Low
CON:RT	Some concerns	Low risk	No concerns	Major concerns	No concerns	No concerns	Low
CON:SE	Some concerns	Low risk	No concerns	Major concerns	No concerns	No concerns	Low
CON:WBV	Some concerns	Low risk	No concerns	Major concerns	No concerns	No concerns	Low
CON:WT	Some concerns	Low risk	No concerns	Major concerns	No concerns	No concerns	Low
AT:CT	Some concerns	Low risk	No concerns	Major concerns	No concerns	No concerns	Low
AT:MBE	Some concerns	Low risk	No concerns	Major concerns	No concerns	No concerns	Low
AT:WBV	Some concerns	Low risk	No concerns	Major concerns	No concerns	No concerns	Low
AT:WT	Some concerns	Low risk	No concerns	Major concerns	No concerns	No concerns	Low
CT:MBE	Some concerns	Low risk	No concerns	Major concerns	No concerns	No concerns	Low
CT:RT	Some concerns	Low risk	No concerns	Major concerns	No concerns	No concerns	Low
CT:SE	Some concerns	Low risk	No concerns	Major concerns	No concerns	No concerns	Low
CT:WBV	Some concerns	Low risk	No concerns	Major concerns	No concerns	No concerns	Low
CT:WT	Some concerns	Low risk	No concerns	Major concerns	No concerns	No concerns	Low
MBE:RT	Some concerns	Low risk	No concerns	Major concerns	No concerns	No concerns	Low
MBE:SE	Some concerns	Low risk	No concerns	Major concerns	No concerns	No concerns	Low
MBE:WBV	Some concerns	Low risk	No concerns	Major concerns	No concerns	No concerns	Low
MBE:WT	Some concerns	Low risk	No concerns	Major concerns	No concerns	No concerns	Low
RT:SE	Some concerns	Low risk	No concerns	Major concerns	No concerns	No concerns	Low
RT:WBV	Some concerns	Low risk	No concerns	Major concerns	No concerns	No concerns	Low
RT:WT	Some concerns	Low risk	No concerns	Major concerns	No concerns	No concerns	Low
SE:WBV	Some concerns	Low risk	No concerns	Major concerns	No concerns	No concerns	Low

SE:WT	Some concerns	Low risk	No concerns	Major concerns	No concerns	No concerns	Low
WBV:WT	Some concerns	Low risk	No concerns	Major concerns	No concerns	No concerns	Low

Appendix 10: Funnel plots

The figures show the assessment of small study effect bias in studies on the effects of various exercise modalities on arterial stiffness and endothelial function in older adults. The comparison-adjusted funnel plots pertain to all trials comparing at least one exercise modality versus control for each of the following outcome measures:Flow-Mediated Dilation; Carotid-Femoral Pulse Wave Velocity; Brachial-Ankle Pulse Wave Velocity; Augmentation Index; Heart Rate-Corrected Augmentation Index ; Brachial Systolic Blood Pressure; Systolic Blood Pressure; Diastolic Blood Pressure; Carotid Beta-Stiffness Index (β) ; Ankle-Brachial Index;Femoral-Ankle Pulse Wave Velocity ; Body Mass Index.

Figure S10.1: FMD, Flow-Mediated Dilation

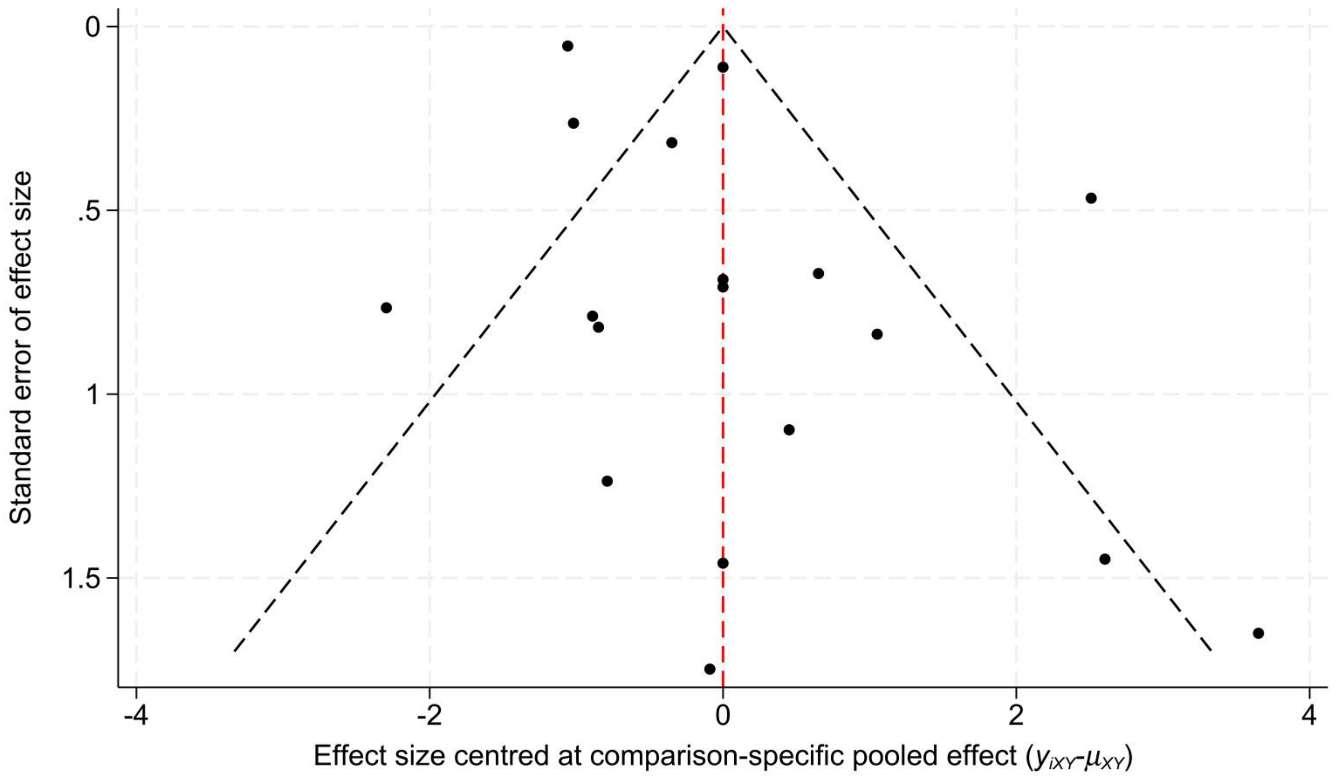

Figure S10.2：cfPWV, Carotid-Femoral Pulse Wave Velocity

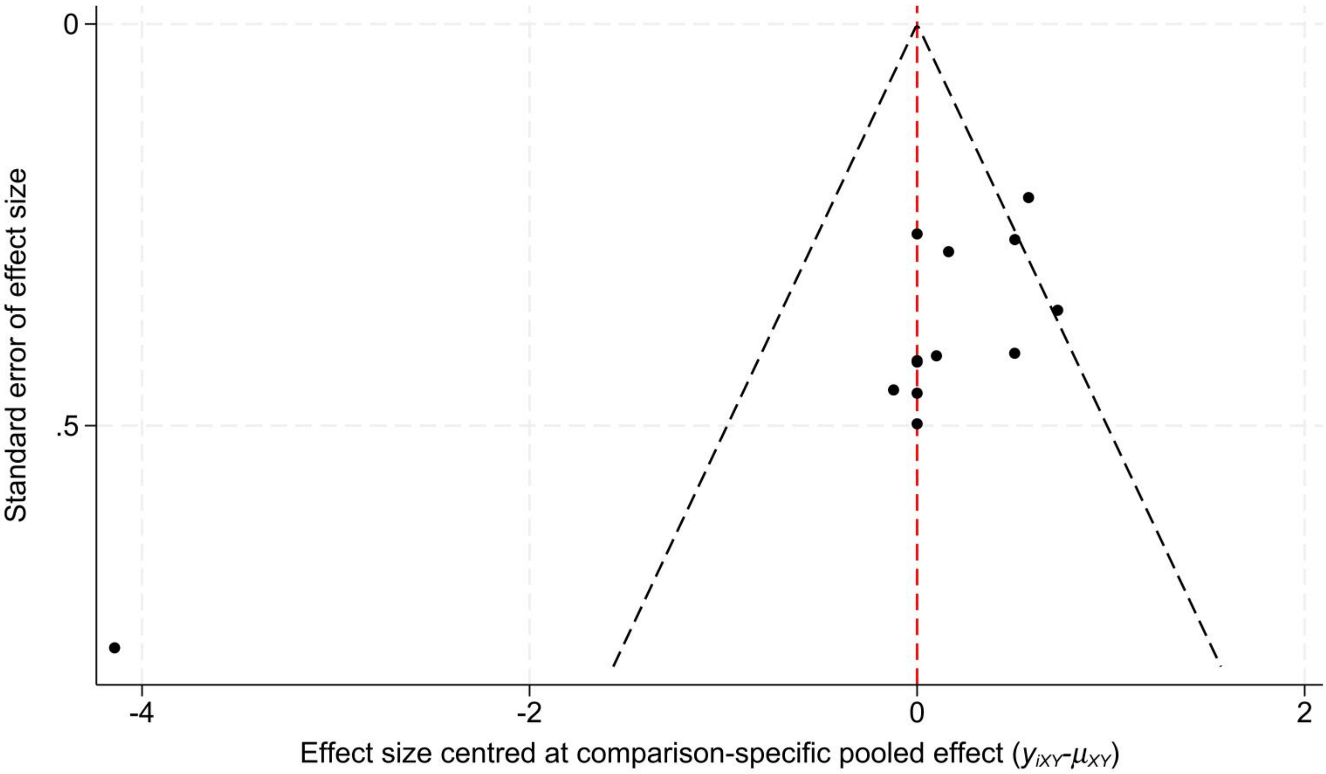

Figure S10.3: baPWV, Brachial-Ankle Pulse Wave Velocity

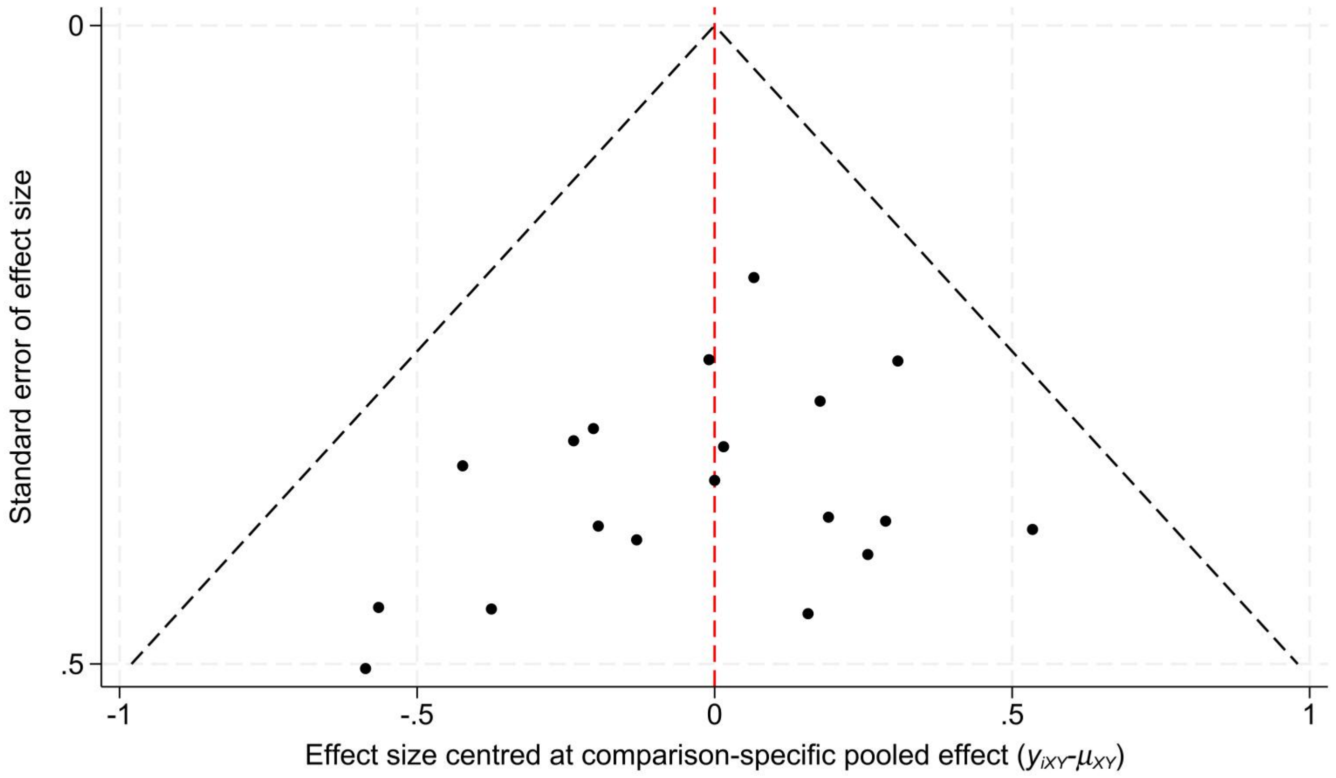

Figure S10.4: AIx, Augmentation Index

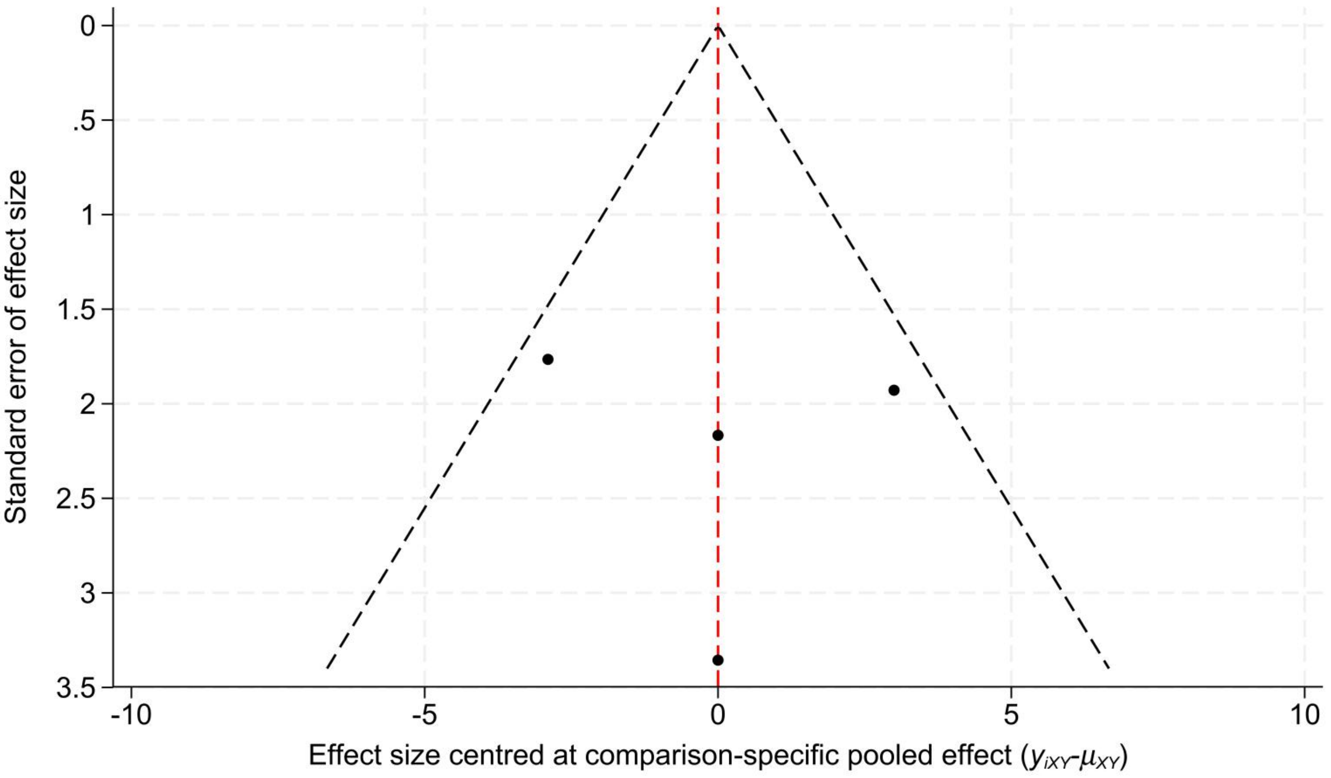

Figure S10.5: AIx@75, Heart Rate-Corrected Augmentation Index

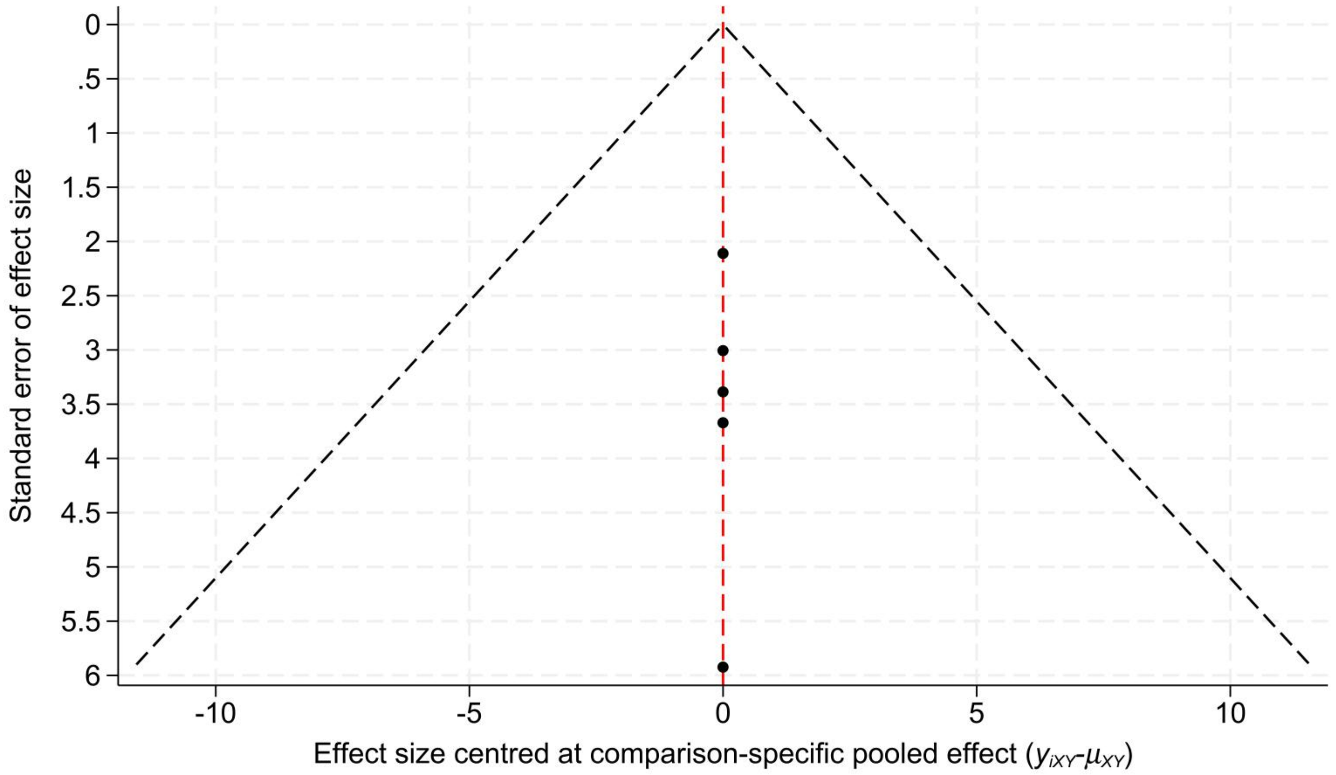

Figure S10.6: BSBP, Brachial Systolic Blood Pressure

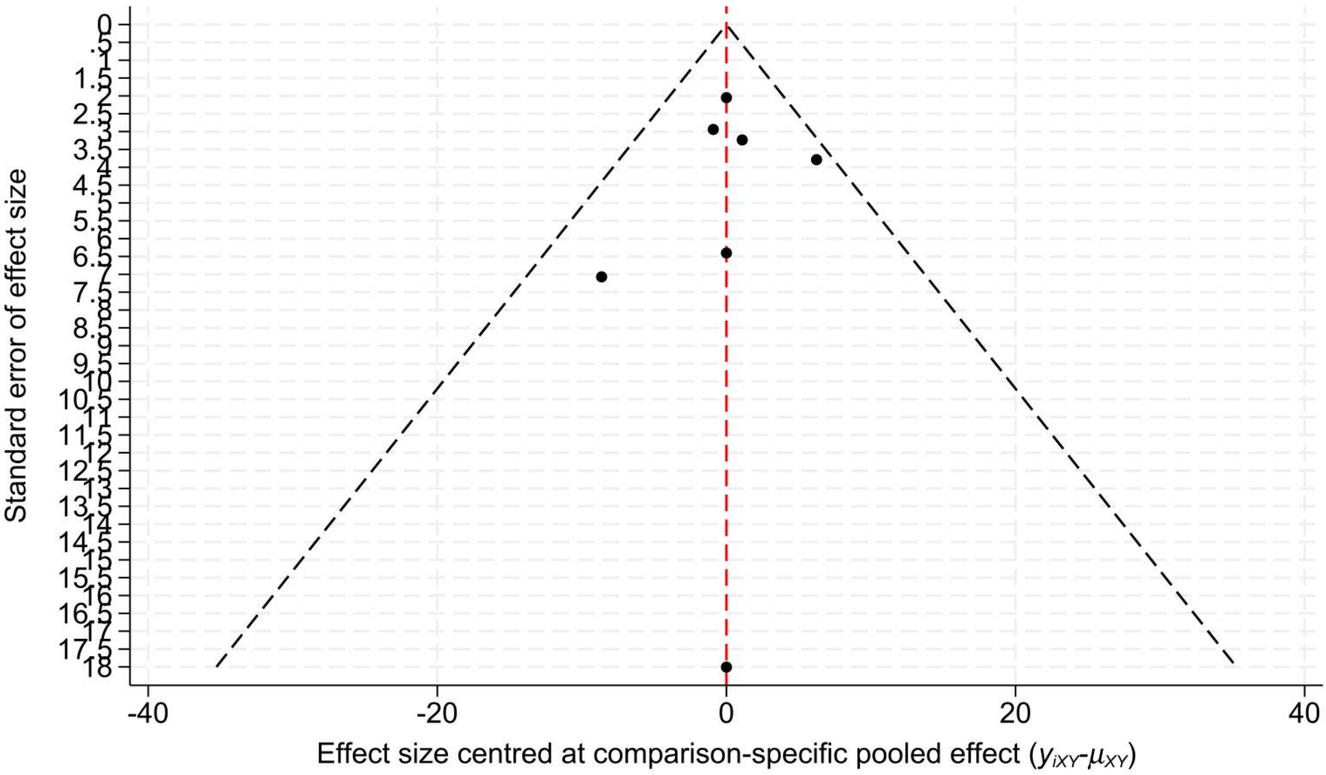

Figure S10.7: SBP, Systolic Blood Pressure

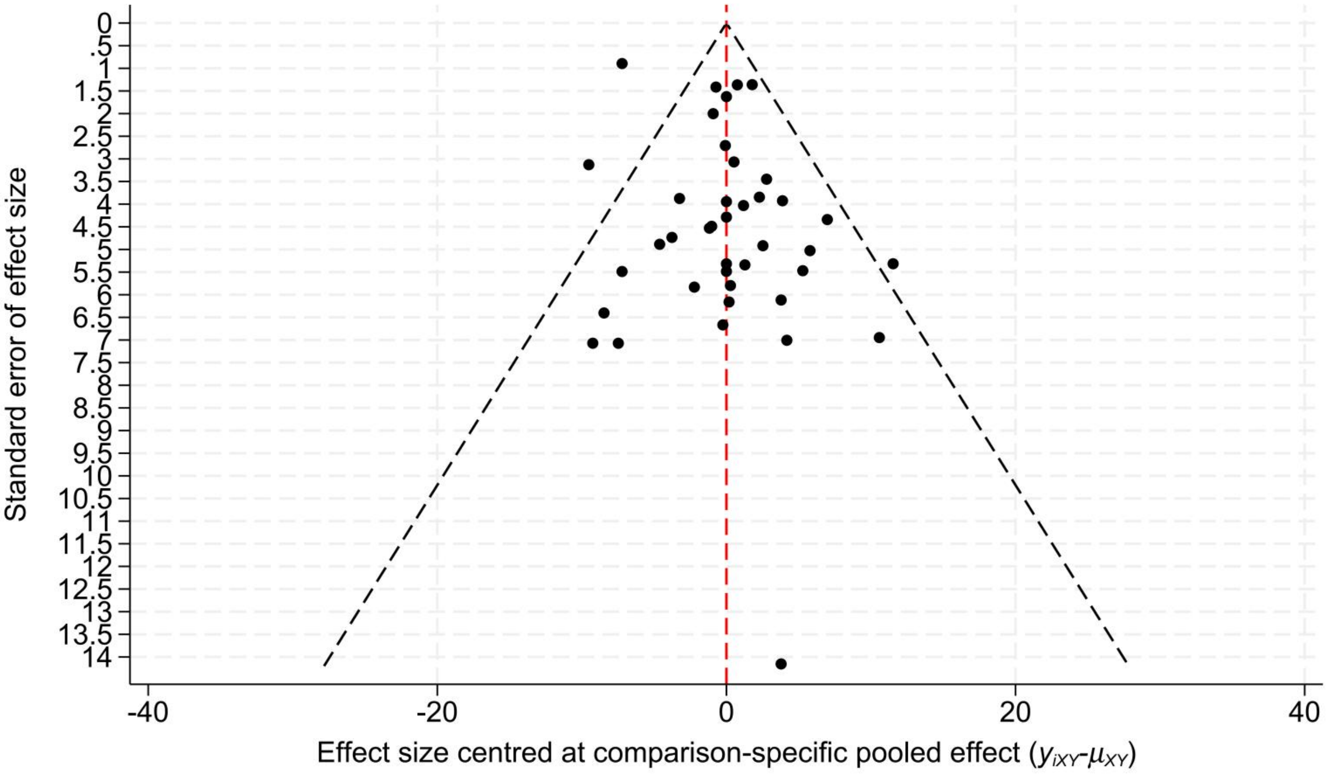

Figure S10.8: DBP, Diastolic Blood Pressure

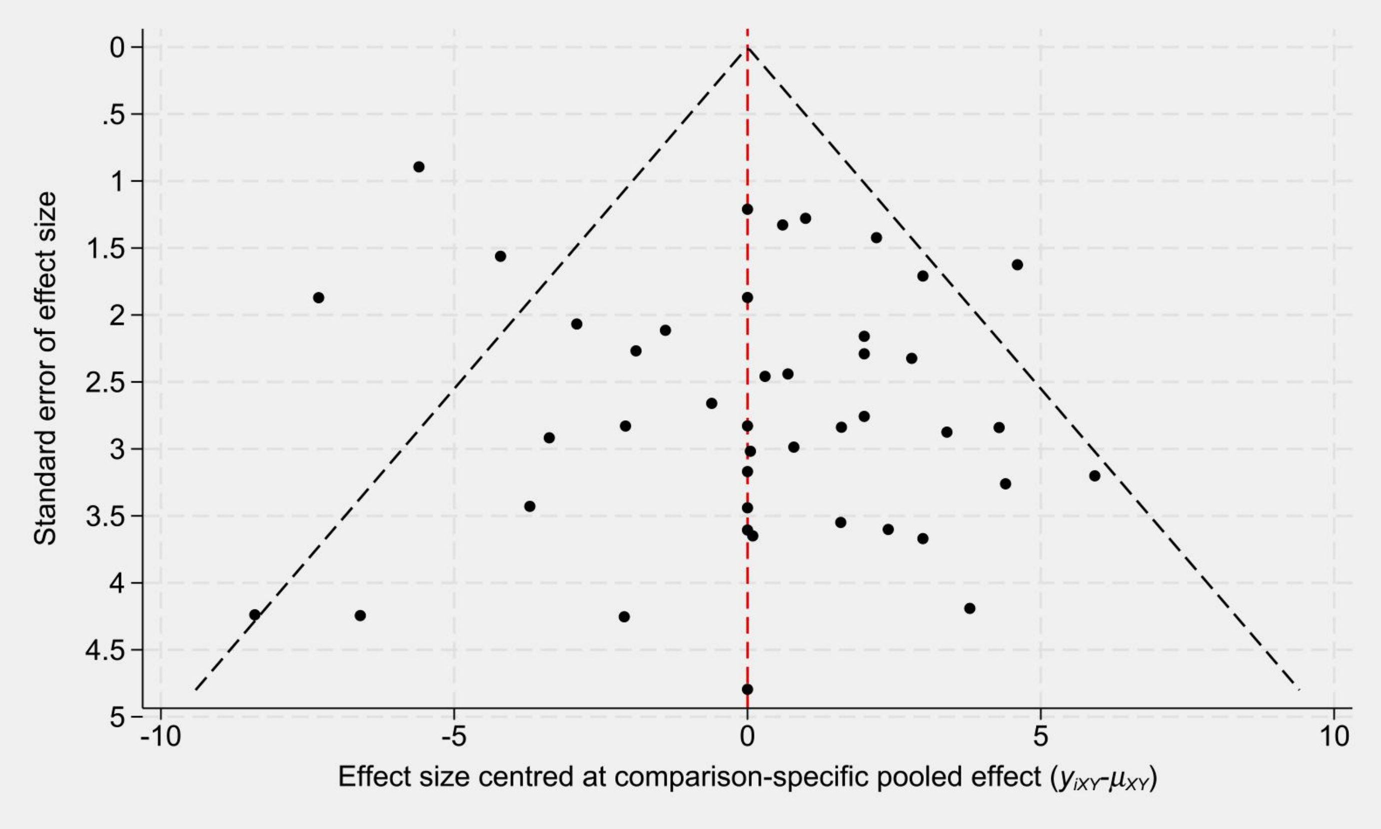

Figure S10.9:β-index,Carotid Beta-Stiffness Index (β)

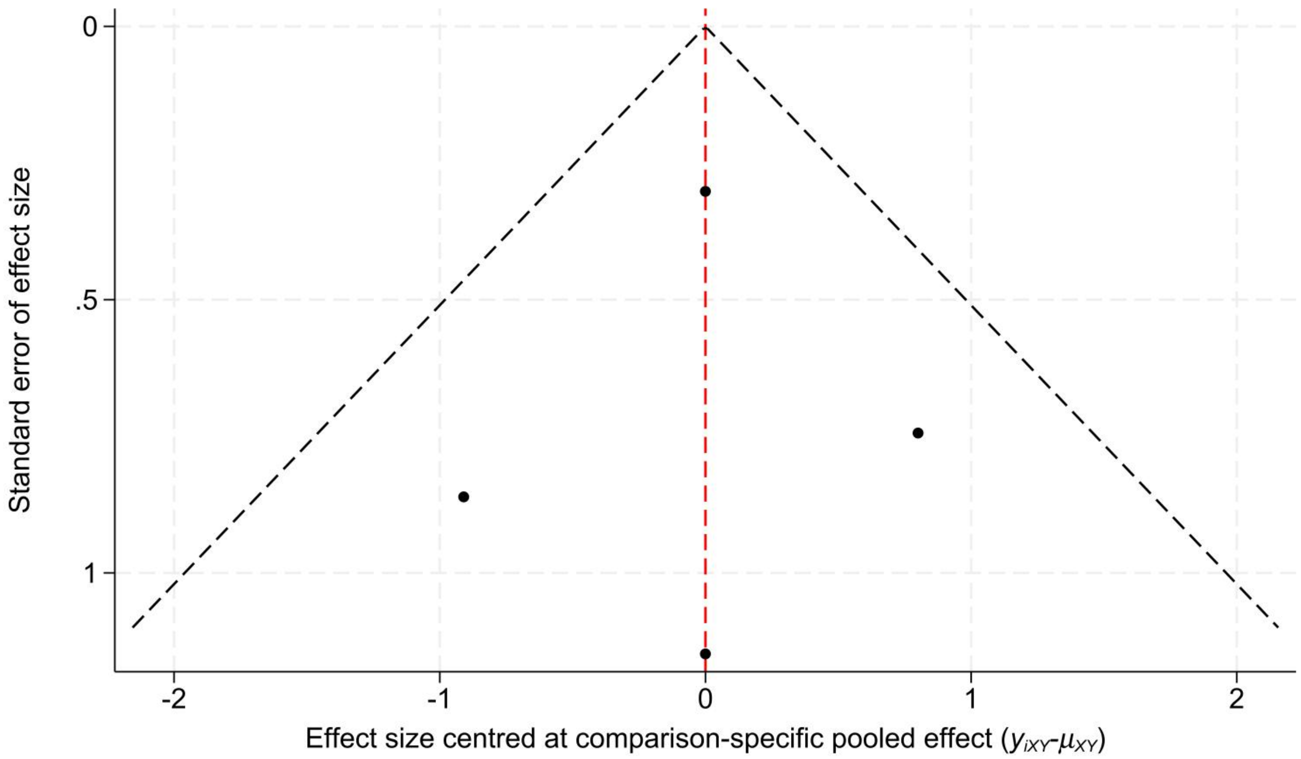

Figure S10.10: ABI, Ankle-Brachial Index

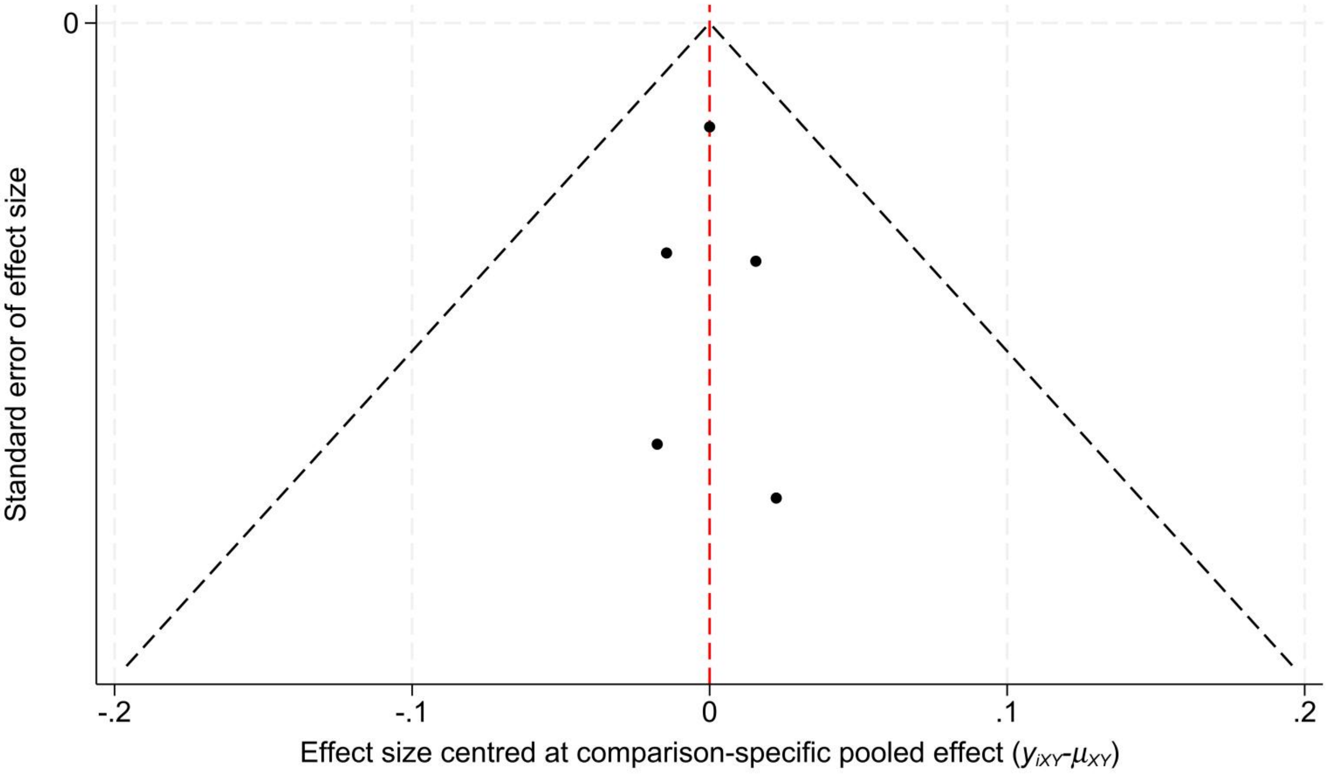

Figure S10.11: faPWV, Femoral-Ankle Pulse Wave Velocity

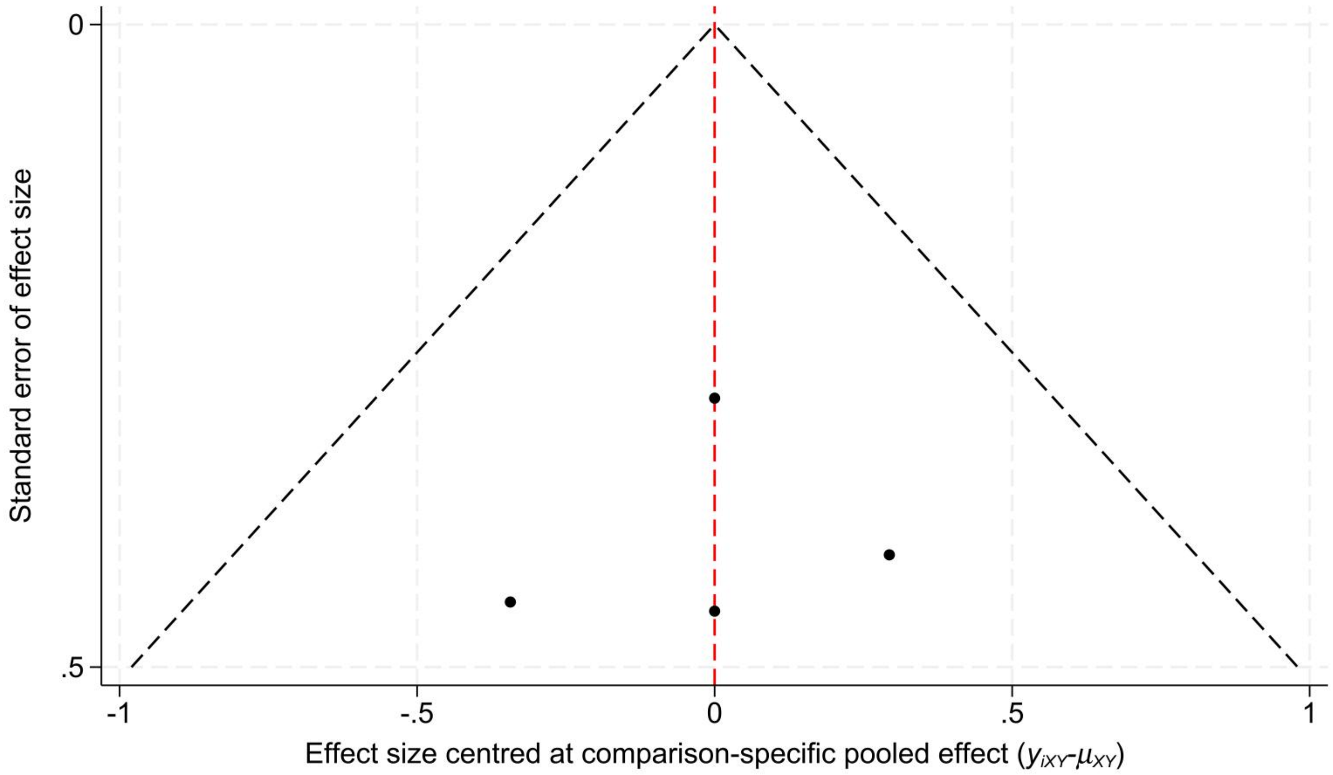

Figure S10.12: BMI, Body Mass Index

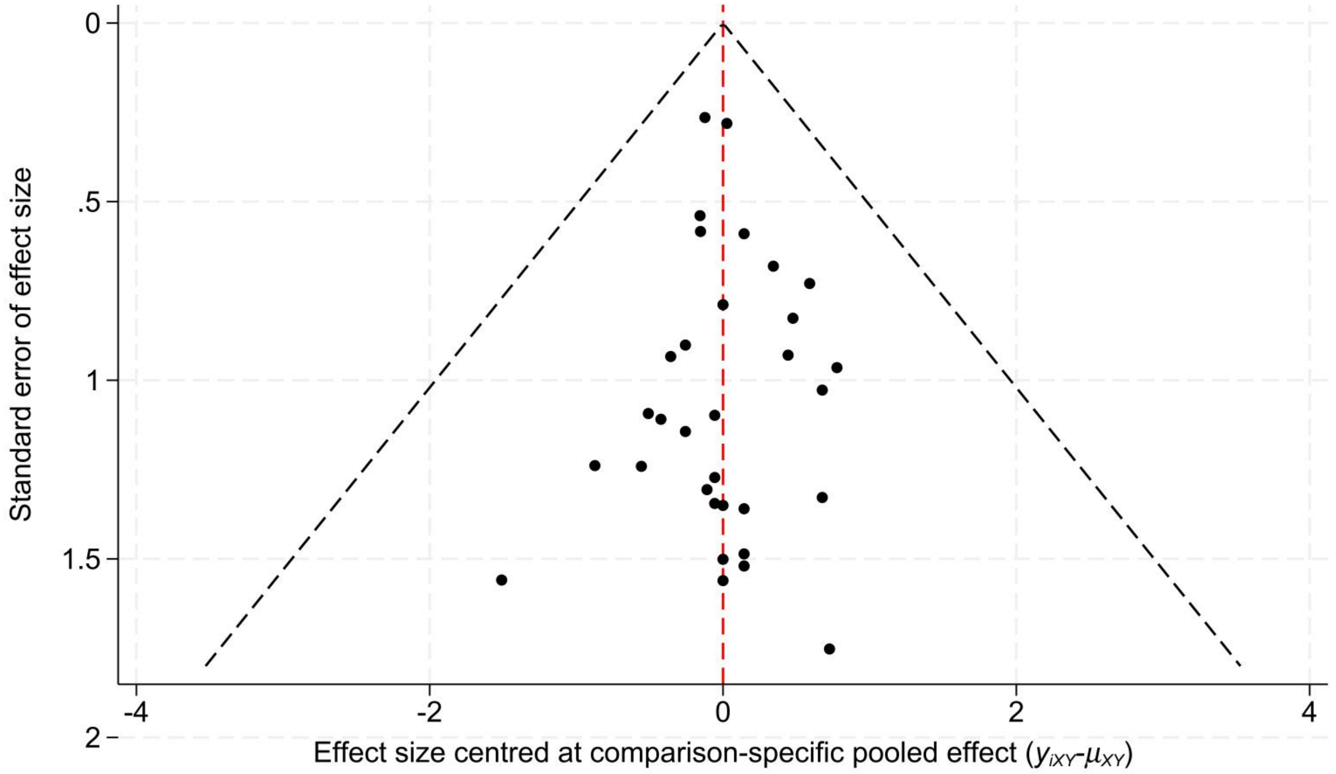

Appendix 11: Subgroup analysis

Figure S11.1: Subgroup analysis for flow-mediated dilation

(A) Forest plot of FMD within the physiological vascular ageing cohort.

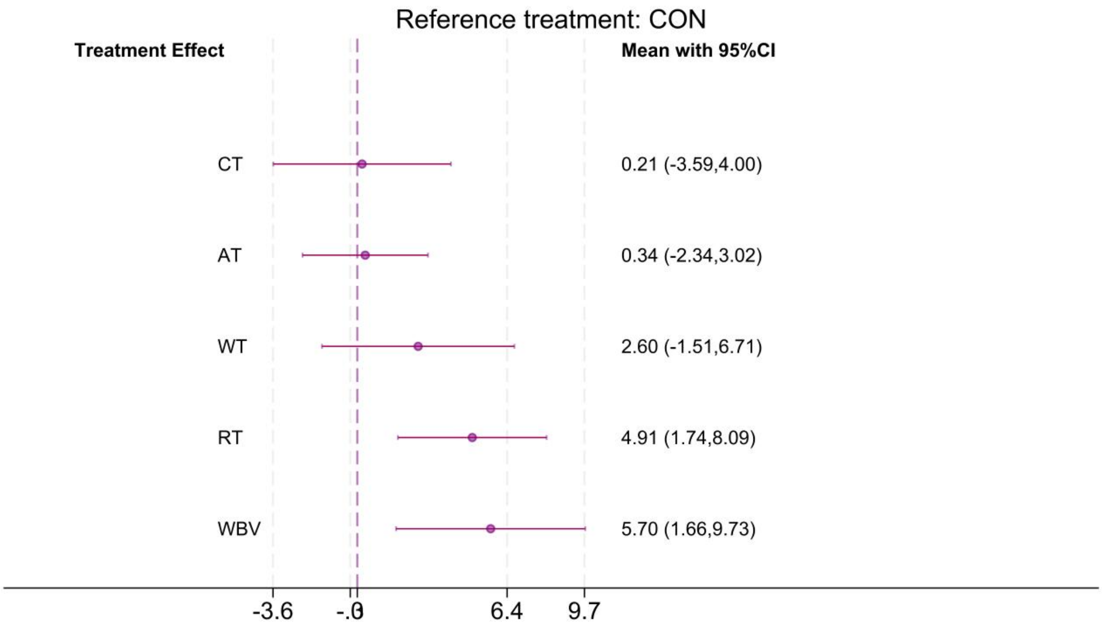

(B) Forest plot of FMD within the pathological endothelial dysfunction cohort.

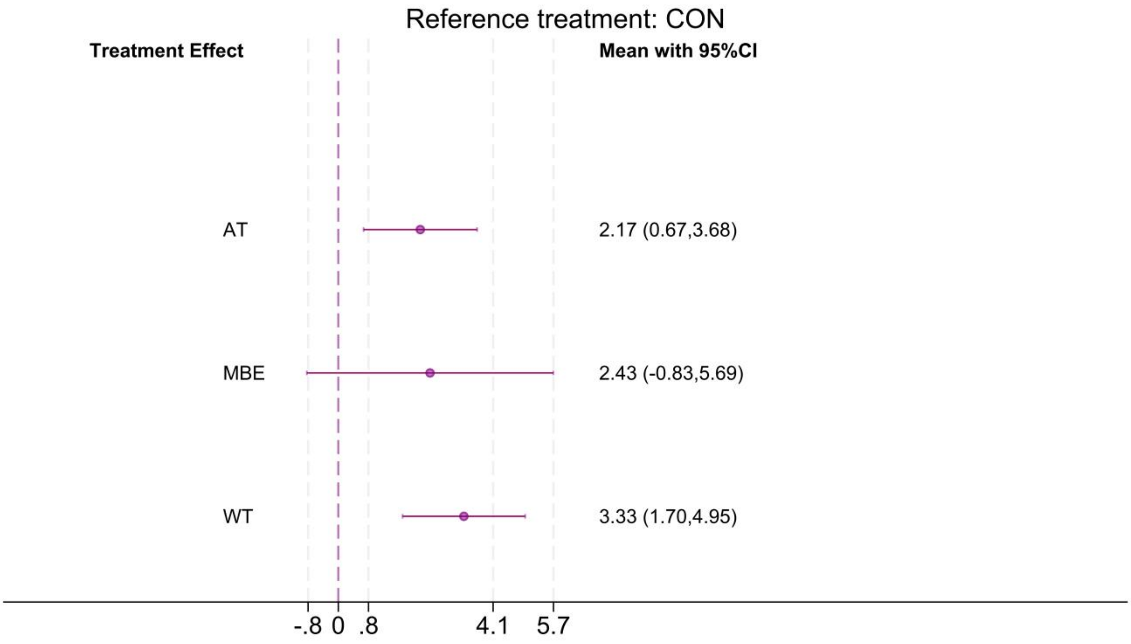

Figure S11.2:. Subgroup analysis for Carotid-Femoral Pulse Wave Velocity

(A) Forest plot of cfPWV within the physiological vascular ageing cohort.

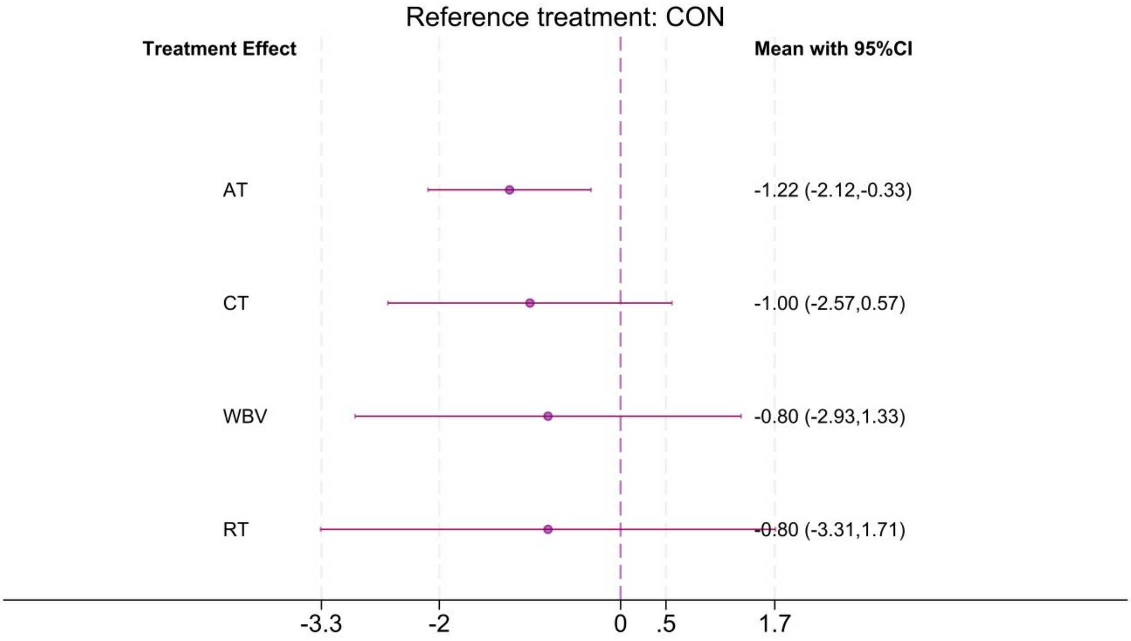

(B) Forest plot of cfPWV within the pathological endothelial dysfunction cohort.

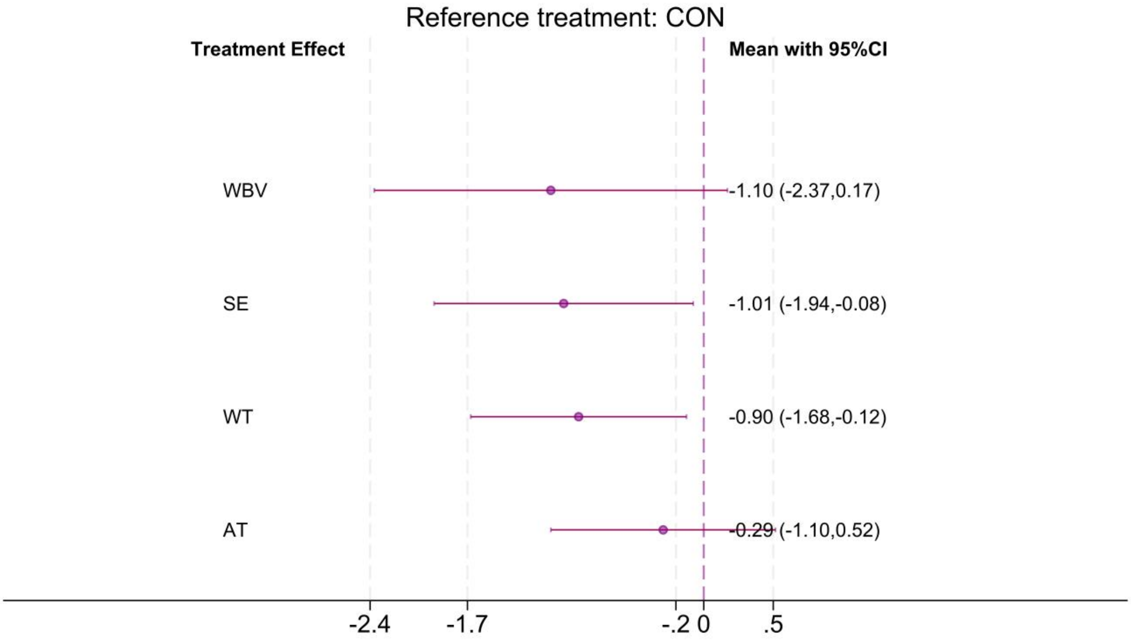

Figure S11.3: Subgroup analysis for brachial–ankle pulse wave velocity

(A) Forest plot of baPWV within the physiological vascular ageing cohort.

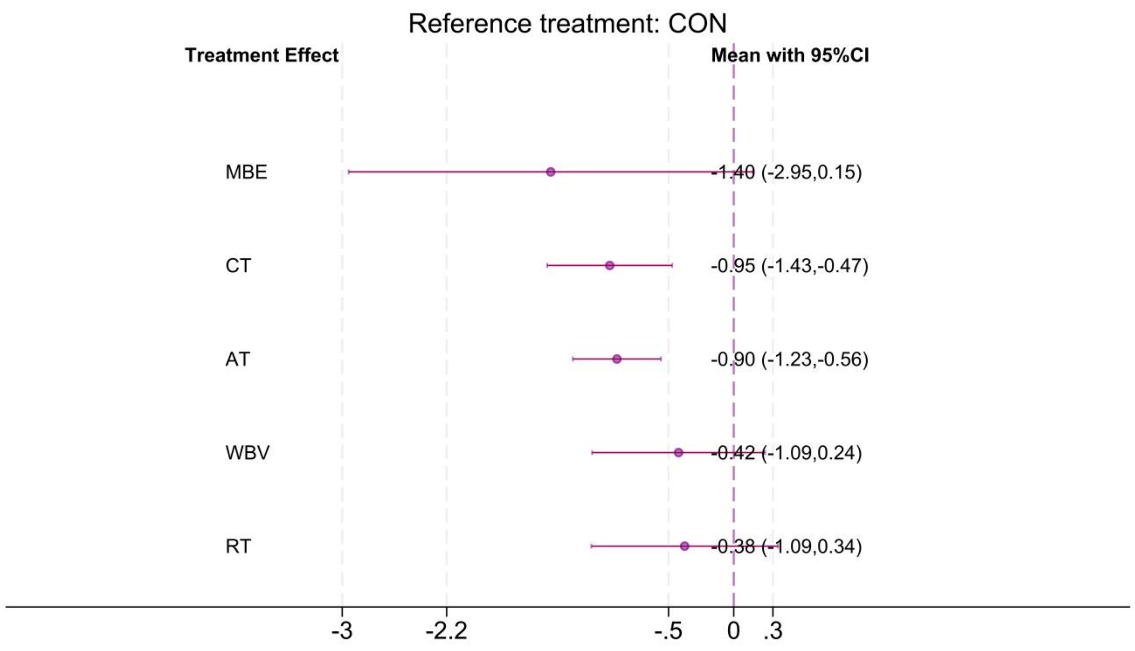

(B) Forest plot of baPWV within the pathological endothelial dysfunction cohort.

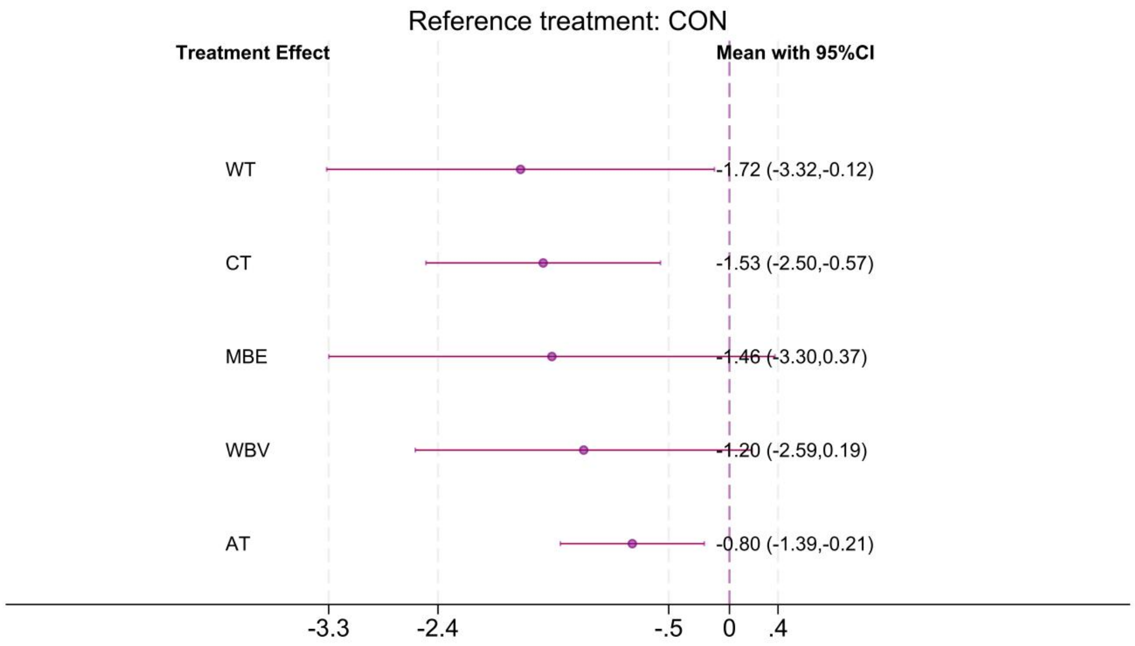

Figure S11.4: Subgroup analysis for systolic blood pressure (SBP)

(A) Forest plot of SBP within the physiological vascular ageing cohort.

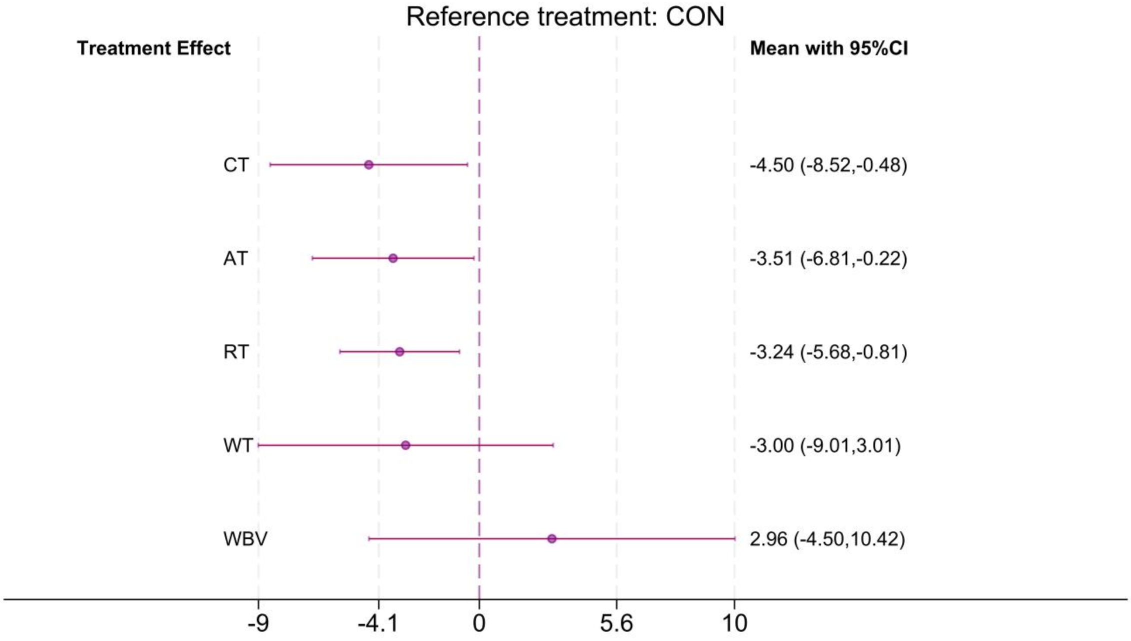

(B) Forest plot of SBP within the pathological endothelial dysfunction cohort.

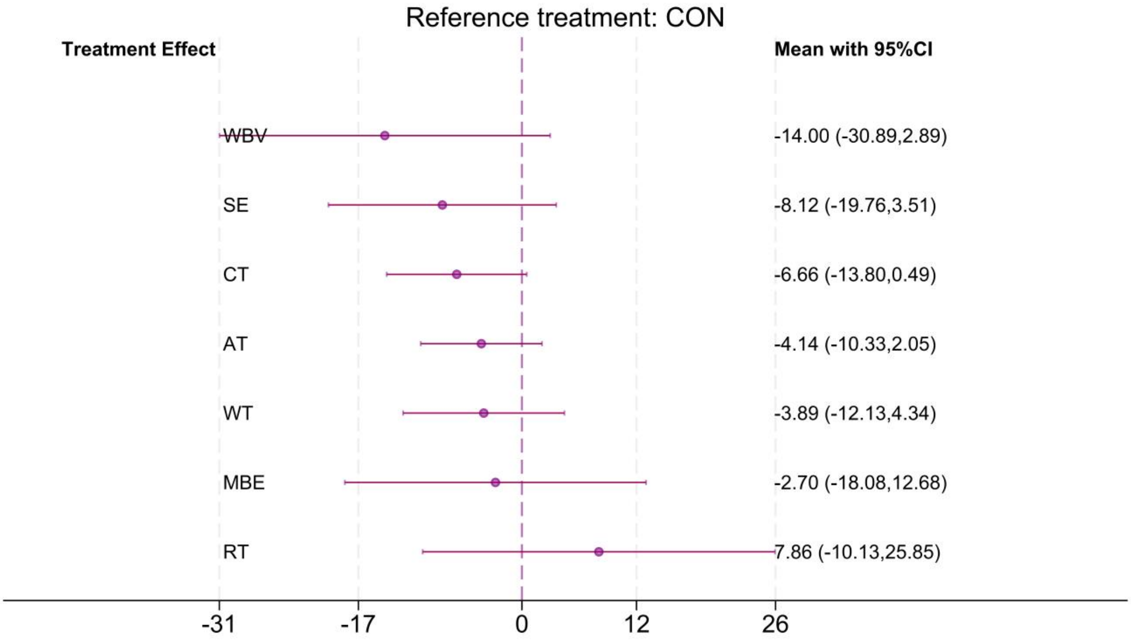

Figure S11.5: Subgroup analysis for diastolic blood pressure (DBP)

(A) Forest plot of DBP within the physiological vascular ageing cohort.

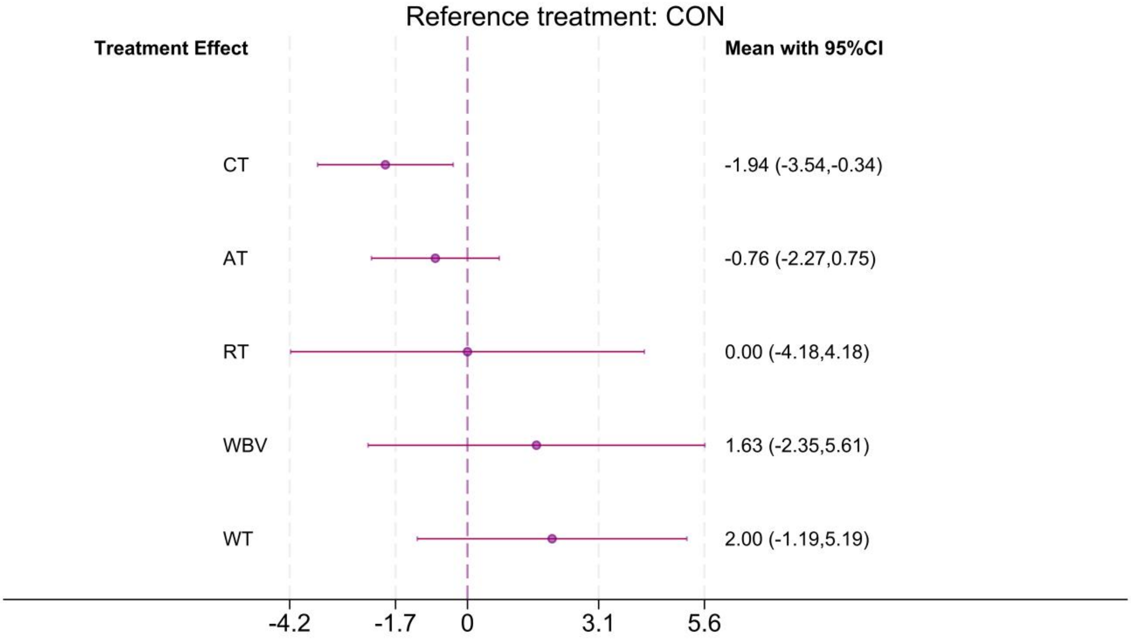

(B) Forest plot of DBP within the pathological endothelial dysfunction cohort.

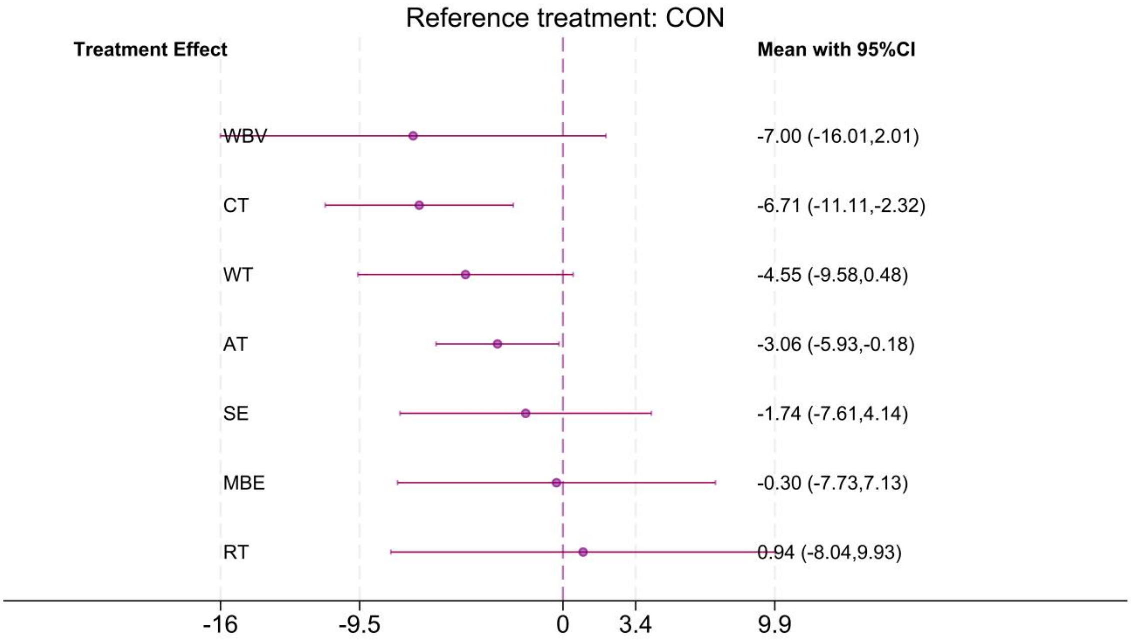

Supplement: Supplementary file 1 [file Datasheet1.pdf]
